# The deacetylation of Foxk2 by Sirt1 reduces chemosensitivity to cisplatin

**DOI:** 10.1111/jcmm.17107

**Published:** 2021-12-06

**Authors:** Xi‐wen Wang, Qi‐qiang Guo, Yang Yu, Ting‐ting Zhou, Si‐yi Zhang, Zhuo Wang, Jing‐wei Liu, Jun Tang, Xiao‐you Jiang, Shan‐shan Wang, Wen‐dong Guo, Hong‐de Xu, Hua‐yi Sun, Zi‐wei Li, Xiao‐yu Song, Jun‐gang Zhao, Liu Cao

**Affiliations:** ^1^ College of Basic Medical Science Key Laboratory of Medical Cell Biology Ministry of Education China Medical University Shenyang China; ^2^ Key Laboratory of Liaoning Province China Medical University Shenyang China; ^3^ Department of Thoracic Surgery Shengjing Hospital of China Medical University Shenyang, Liaoning China

**Keywords:** chemosensitivity, cisplatin, deacetylation, Forkhead box K2, sirtiun 1

## Abstract

In multiple types of cancer, decreased tumour cell apoptosis during chemotherapy is indicative of decreased chemosensitivity. Forkhead box K2 (FOXK2), which is essential for cell fate, regulates cancer cell apoptosis through several post‐translational modifications. However, FOXK2 acetylation has not been extensively studied. Here, we evaluated the effects of sirtiun 1 (SIRT1) on FOXK2 deacetylation. Our findings demonstrated that SIRT1 inhibition increased FOXK2‐induced chemosensitivity to cisplatin and that K223 in FOXK2 was acetylated. Furthermore, FOXK2 K223 deacetylation reduced chemosensitivity to cisplatin in vitro and in vivo. Mechanistically, FOXK2 was acetylated by the acetyltransferase cAMP response element binding protein and deacetylated by SIRT1. Furthermore, cisplatin attenuated the interaction between FOXK2 and SIRT1. Cisplatin or SIRT1 inhibition enhanced FOXK2 acetylation, thereby reducing the nuclear distribution of FOXK2. Additionally, FOXK2 K223 acetylation significantly affected the expression of cell cycle–related and apoptosis‐related genes in cisplatin‐stimulated cancer cells, and FOXK2 K223 hyperacetylation promoted mitotic catastrophe, which enhanced chemosensitivity to cisplatin. Overall, our results provided insights into the mechanisms of SIRT1‐mediated FOXK2 deacetylation, which was involved in chemosensitivity to cisplatin.

## 1 INTRODUCTION

Forkhead box K2 (FOXK2) has important regulatory functions in the proliferation, migration, invasion, metastasis, metabolism and apoptosis of cancer cells.^1^ Moreover, FOXK2 acts as a suppressor in breast cancer, renal clear cell carcinoma, non‐small cell lung cancer, glioma and gastric cancer^2^, ^3^, ^4^, ^5^, ^6^ but has oncogenic roles in leukaemia, breast cancer, prostate cancer, liver cancer, non‐small cell lung cancer and hepatocellular carcinoma.^7^, ^8^, ^9^, ^10^, ^11^, ^12^, ^13^ Chemotherapy is essential for improving outcomes (eg preventing or decreasing recurrence, progression and metastasis) in patients with cancer,^14^, ^15^, ^16^, ^17^ and FOXK2 has been shown to reduce the chemosensitivity of paclitaxel in breast cancer cells.^18^ Nevertheless, little is known about other post‐translational modifications (PTMs) involved in FOXK2‐mediated chemosensitivity.

FOXK2 regulates various cellular functions via PTM. Ser368 and Ser423 of FOXK2 are phosphorylated by the cyclin‐dependent kinase 1 (CDK1)/cyclinB kinase complex, thereby decreasing apoptosis and FOXK2 protein stability.^19^ In addition, DNA damage induces the phosphorylation of FOXK2 at Ser61 and traps FOXK2 in the cytoplasm, resulting in increased autophagy and chemotherapy resistance.^20^ However, it remains unclear whether FOXK2 is regulated by acetylation.

Lysine acetylation has been reported to be involved in tumour progression by regulating multiple functions of cancer cells. Growing evidence has indicated that non‐histone acetylation also controls multiple cellular functions.^21^, ^22^, ^23^ The transcriptional activity of numerous non‐histone proteins, such as FOXO,^24^ peroxisome proliferator‐activated receptor γ coactivator‐1α,^25^ nuclear factor‐κB,^26^ and p53,^27^ is regulated by sirtiun 1 (SIRT1). Following DNA damage, SIRT1 inhibits apoptosis by deacetylating p53 and FOXO.^24^, ^28^, ^29^ SIRT1 act as either an oncogene or a tumour suppressor depending on its corresponding downstream effectors.^30^

In this study, we characterized FOXK2 K223 deacetylation and evaluated the effects of this PTM on chemosensitivity to cisplatin in vitro and in vivo. We also discussed the effects of the acetylation status of FOXK2 on mitotic catastrophe. Overall, our findings provided insights into the molecular mechanisms regulating chemosensitivity to cisplatin.

## 2 MATERIALS AND METHODS

### 2.1 Cell culture and plasmids

The human cancer cell lines H1299, HCT116 and HeLa as well as human embryonic kidney (HEK) 293T cells were purchased from Shanghai Cell Bank (Chinese Academy of Sciences, Shanghai, China). 293T and HeLa cells were cultured in high‐glucose Dulbecco's modified Eagle's medium supplemented with 10% foetal bovine serum (FBS, inactivated at 56°C for 40 min), 100 U/ml penicillin and 100 μg/ml streptomycin. H1299 and HCT116 cells were cultured in Roswell Park Memorial Institute 1640 supplemented with 10% FBS, penicillin and streptomycin. All cells were maintained in a 37°C incubator with a humidified atmosphere containing 5% CO_2_.

Flag/HA‐tagged FOXK2 and Flag‐tagged FOXK2 (K223R) were purchased from Shanghai GeneChem Company. Myc‐general control non‐repressed protein 5 (GCN5), Flag‐P300 and Flag‐cAMP response element binding protein (CBP) were provided by Q. Lei (Shanghai Medical College, Shanghai, China). Flag‐p300/CBP‐associated factor (PCAF) was a gift from W. Zhu (Shenzhen University, Shenzhen, China). Myc/Flag‐SIRT1 wild‐type (WT) and H363Y (deacetylase‐inactive mutant) were obtained from Cao's laboratory.

### 2.2 Antibodies and reagents

Antibodies against acetylated lysine (cat. no. 9441; 1:1000), FOXK2 (cat. no. 12008; 1:1000), CBP (cat. no. 7389; 1:1000), Lamin B1 (cat. no. 13435; 1:1000), HA‐Tag (cat. no. 3742; 1:1000), phospho‐histone H3 (cat. no. 53348; 1:1000), histone H3 (cat. no. 4499; 1:1000), cyclin B1 (cat. no. 4135; 1:1000), cleaved‐poly (ADP‐ribose) polymerase (PARP; cat. no. 5625; 1:1000), cleaved‐caspase 3 (cat. no. 9661; 1:1000) and α‐tubulin (cat. no. 2144; 1:5000) were purchased from Cell Signaling Technology (MA, USA). Antibodies against Myc‐Tag (cat. no. M4439; 1:2000) and Flag‐Tag (cat. no. F1804; 1:2000) were from Sigma‐Aldrich (MO, USA). Antibodies against SIRT1 (1:1000) were from Millipore (MA, USA). Antibodies against β‐actin (cat. no. AC004; 1:10000) and anti‐mouse/rabbit IgG secondary antibodies were from ABclonal (Wuhan, China).

The SIRT1 inhibitor EX527 (cat. no. S1541), cisplatin (cat. no. S1166), doxorubicin (cat. no. S1208) and etoposide (cat. no. S1225) were from Selleck. Nicotinamide (NAM; cat. no. 72340), trichostatin A (TSA; cat. no. T8552) and 4′,6‐diamidino‐2‐phenylindole (DAPI; cat. no. D9542) were from Sigma. Protease Inhibitor Cocktail (cat. no. HY‐K0010) and Deacetylase Inhibitor Cocktail (cat. no. HY‐K0030) were from MedChemExpress. Protein‐A/G agarose beads were from Santa Cruz Biotechnology (Santa Cruz, CA, USA).

### 2.3 Immunoprecipitation and Western blotting

For immunoprecipitation, cells were lysed with IP lysis buffer (50 mM Tris pH 7.4, 0.25% sodium deoxycholate, 150 mM NaCl, 1% Triton X‐100, 0.5% NP‐40, 1 mM ethylenediaminetetraacetic acid) supplemented with protease inhibitor and deacetylase inhibitor (for acetylation analysis). Cell lysates (1–4 mg) were then mixed with a primary antibody (2 μg) at 4°C overnight and subsequently with protein‐A/G beads. Next, the complexes were washed three times with IP lysis buffer containing deacetylase inhibitor when appropriate. Protein beads were then eluted with 2× loading buffer and boiled. Finally, the samples were subjected to sodium dodecyl sulphate‐polyacrylamide gel electrophoresis and analysed by Western blotting.

### 2.4 Cytoplasmic and nuclear protein extraction

Cytoplasmic and nuclear protein extraction was performed using a Minute Cytoplasmic and Nuclear Extraction Kit (Invent Biotechnologies, Shanghai, China) according to the manufacturer's instructions.

### 2.5 Immunofluorescence

Cells were washed twice with phosphate‐buffered saline (PBS), fixed with 4% paraformaldehyde for 20 min and washed with PBS. The cells were then permeabilized with 0.25% Triton X‐100 for 10 min, blocked with 5% bovine serum albumin for 1 h at room temperature and incubated with primary antibodies overnight at 4°C. Cells were washed three times with PBS, incubated with the corresponding fluorescent secondary antibody for 1 h at room temperature and incubated with DAPI at room temperature for 5 min in the dark. Finally, the cells were imaged using an immunofluorescence microscope or confocal microscope (Olympus).

### 2.6 Lentiviral infection

Lentiviral reagents expressing control shRNA (shCtrl), shRNA against FOXK2 (shFOXK2), control overexpression (shFOXK2‐ovNC), FOXK2‐WT overexpression (shFOXK2‐ovWT) and FOXK2‐K223R overexpression (shFOXK2‐ovK223R) sequences were purchased from Shanghai GeneChem Company. After lentivirus infection, stably silenced or overexpressing cell lines were selected by treatment with puromycin (4 μg/ml) for 48 h, according to the manufacturer's instructions.

### 2.7 RNA‐Seq processing and data analysis

One microgram total RNA per sample was used as input material for RNA sample preparations. Clustering of index‐coded samples was performed using a TruSeq PE Cluster Kit v3‐cBot‐HS (Illumina, San Diego, CA, USA) according to the manufacturer's instructions. Following cluster generation, the library preparations were sequenced on an Illumina Novaseq platform.

### 2.8 Cell viability assay

Approximately 1.5 × 10^4^ cells/well were seeded in triplicate into 96‐well plates. After 24 h, cells were treated with cisplatin or doxorubicin. A mixture of 10 μl Cell Counting Kit (CCK)‐8 reagent (Bimake, USA) and 90 μl medium was added to the cells for 1–2 h, and cell viability was evaluated on 3–4 consecutive days. The absorbance at 450 nm was measured daily using an absorbance reader (TECAN, Switzerland).

### 2.9 Analysis of apoptosis by flow cytometry

In total, 3 × 10^5^ cells/well in 6‐well plates were treated with chemotherapeutic drugs for the indicated times, and the harvested cells were then washed with PBS and stained with APC and 7‐AAD. The apoptosis rate was determined using an Annexin V‐APC/7‐AAD kit (KeyGEN, BioTech; cat. no. KGA1026) according to the manufacturer's protocol. The data were analysed using BD Accuri C6 and FlowJo software.

### 2.10 Tumour xenografts

shFOXK2‐ovWT and shFOXK2‐ovK223R H1299 cells, which showed stable silencing of FOXK2 or overexpression of FOXK2‐WT/K223R, respectively, were subcutaneously inoculated in the right flanks of 20 (*n* = 10, each group) 6‐week‐old female BALB/C nude mice (Huafukang Biosciences, China). Each mouse received 150 μl of a mixture (medium:Matrigel = 1:1) of 4 × 10^6^ cells with Matrigel (BD Biosciences). When the volume of tumours reached 50–100 mm^3^, mice were randomly assigned into two groups again, that is the vehicle control group (0.9% saline) and the cisplatin group (2.5 mg/kg). All mice were intraperitoneally injected three times per week. Tumour dimensions were measured every 3 days with callipers and the volume calculated using the following formula: volume (mm^3^) = L × W^2^. Mice were sacrificed, and tumours were removed on day 28 after injection. Data analysis was performed using unpaired Student's t tests.

### 2.11 Statistics

For cell viability, flow cytometric apoptosis evaluation and protein quantification, data were described as means ± standard deviations from three independent experiments. For xenograft assays, data were described as means ± standard errors of the means from five mice. All statistical analyses were conducted using Student's t tests (two groups) or one‐way analysis of variance (three or more groups) using GraphPad Prism (8.0).

## 3 RESULTS

### 3.1 SIRT1 inhibition enhanced FOXK2‐induced chemosensitivity to cisplatin via acetylation at K223 of FOXK2

**FIGURE 1:**
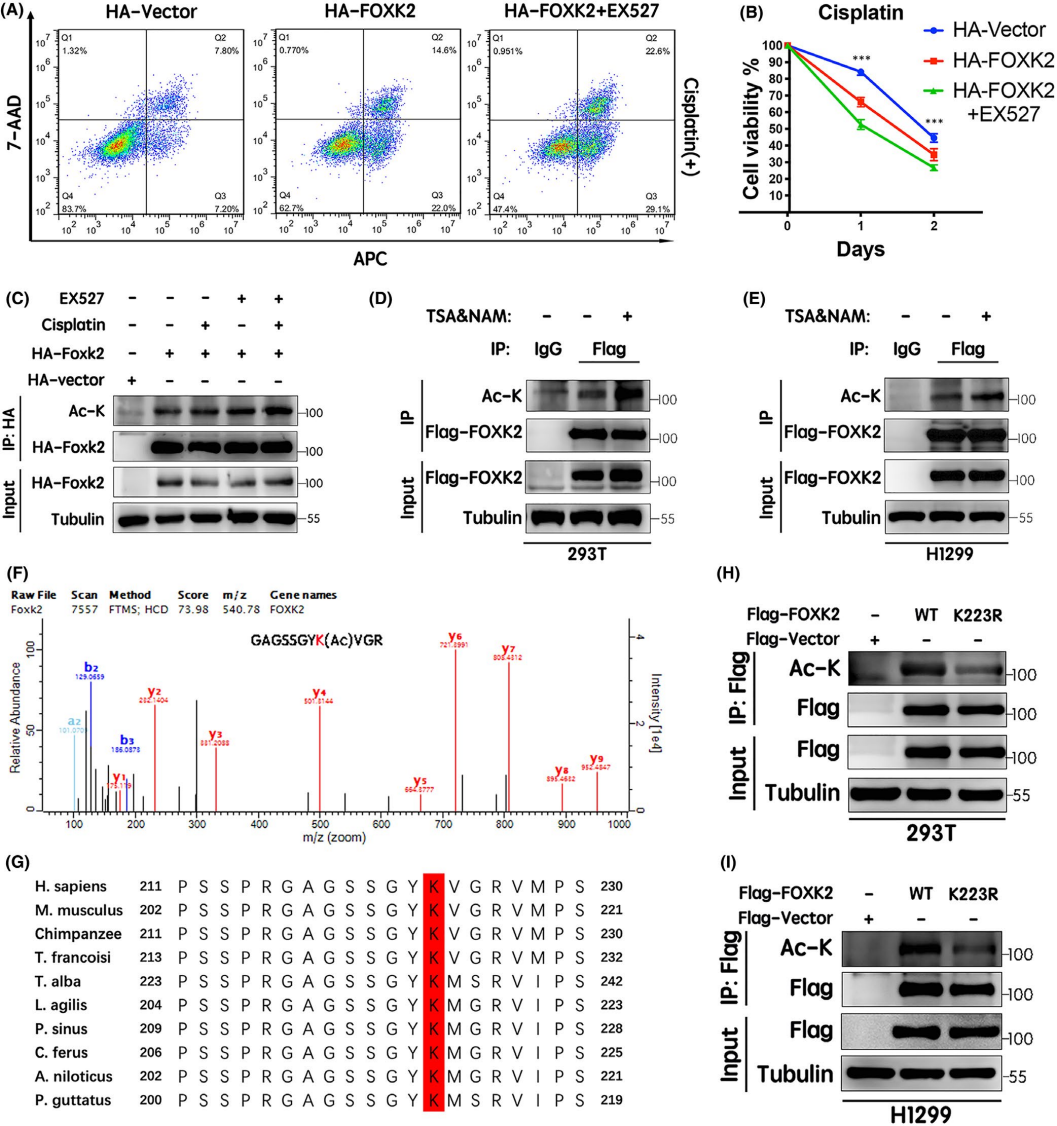
SIRT1 inhibition increased FOXK2‐induced chemosensitivity to cisplatin via acetylation at K223. (A) H1299 cells ectopically expressing HA‐tagged FOXK2 were treated with EX527 (0.5 μM, 9 h) and cisplatin (15 μM, 18 h). Flow cytometry was performed to assess apoptosis. (B) CCK‐8 cytotoxicity assays were performed to evaluate the effects on cell viability. *p < 0.05, **p < 0.01, ***p < 0.001. (C) 293T cells ectopically expressing HA‐tagged FOXK2 were treated with EX527 (0.5 μM, 9 h), and cisplatin (15 μM, 1 h). FOXK2 acetylation levels were detected by immunoprecipitation and Western blotting using anti‐lysine antibodies. (D and E) After overexpressing Flag‐FOXK2, 293T and H1299 cells were treated with or without TSA (1 mM) and NAM (5 mM) for 6 h. Cell lysates were then immunoprecipitated with anti‐Flag antibodies, and acetylation levels were determined by immunoprecipitation and Western blotting. (F) Flag‐tagged FOXK2 was transfected into 293T cells. After 24 h, the cells were treated with the deacetylase inhibitors TSA (1 mM) and NAM (5 mM) for another 6 h. FOXK2 was immunoprecipitated with an anti‐Flag antibody, and the acetylation site was analysed by mass spectrometry. (G) The protein sequences of FOXK2 homologs in different species were aligned. K223 (red) in FOXK2 was conserved. (H and I) The indicated Flag‐FOXK2‐WT and Flag‐FOXK2‐K223R plasmids were transfected into 293T and H1299 cells. Immunoprecipitation and Western blotting were performed to assess acetylation levels

To investigate whether the PTM of FOXK2 affected chemosensitivity to cisplatin in cancer cells, we treated H1299 cells with the SIRT1 selective inhibitor EX527. Our results showed that this inhibitor further increased chemosensitivity to cisplatin in H1299 cells compared with FOXK2 overexpression‐induced chemosensitivity to cisplatin (Figure 1A,B). Moreover, EX527 further enhanced the acetylation of FOXK2, which was induced by cisplatin (Figure 1C). Therefore, we hypothesized that FOXK2 hyperacetylation increased chemosensitivity to cisplatin.

To confirm whether FOXK2 was acetylated, Flag‐tagged FOXK2 was ectopically expressed in 293T and H1299 cells, and the cells were then treated with TSA, an inhibitor of histone deacetylase I and II, and simultaneously with NAM, an inhibitor of the SIRT family of deacetylases. The acetylation level of FOXK2 was detected using anti‐acetylated lysine antibodies. Immunoprecipitation and Western blotting showed that FOXK2 acetylation was significantly enhanced after treatment with TSA and NAM (Figure 1D,E).

Mass spectrometry was then performed using Flag‐tagged FOXK2 in the presence or absence of TSA and NAM to identify the acetylation site on FOXK2. Results showed that K223 of FOXK2 was acetylated (Figure 1F). Protein sequence alignment of FOXK2 homologs showed that K223 is evolutionarily conserved in different species from *Pantherophis guttatus* to mammals (Figure 1G). To confirm this result, we mutated the K223 to arginine (K223R) and ectopically expressed Flag‐FOXK2‐WT and Flag‐FOXK2‐K223R in 293T and H1299 cells. Immunoprecipitation and Western blotting showed that mutation from K to R resulted in a dramatic reduction in FOXK2 acetylation, verifying that Lys223 was indeed the acetylation site for FOXK2 (Figure 1H,I). These results suggested that FOXK2 hyperacetylation at K223 increased chemosensitivity to cisplatin.

### 3.2 FOXK2 K223 deacetylation reduced the chemosensitivity of cancer cells to cisplatin in vitro and in vivo

**FIGURE 2:**
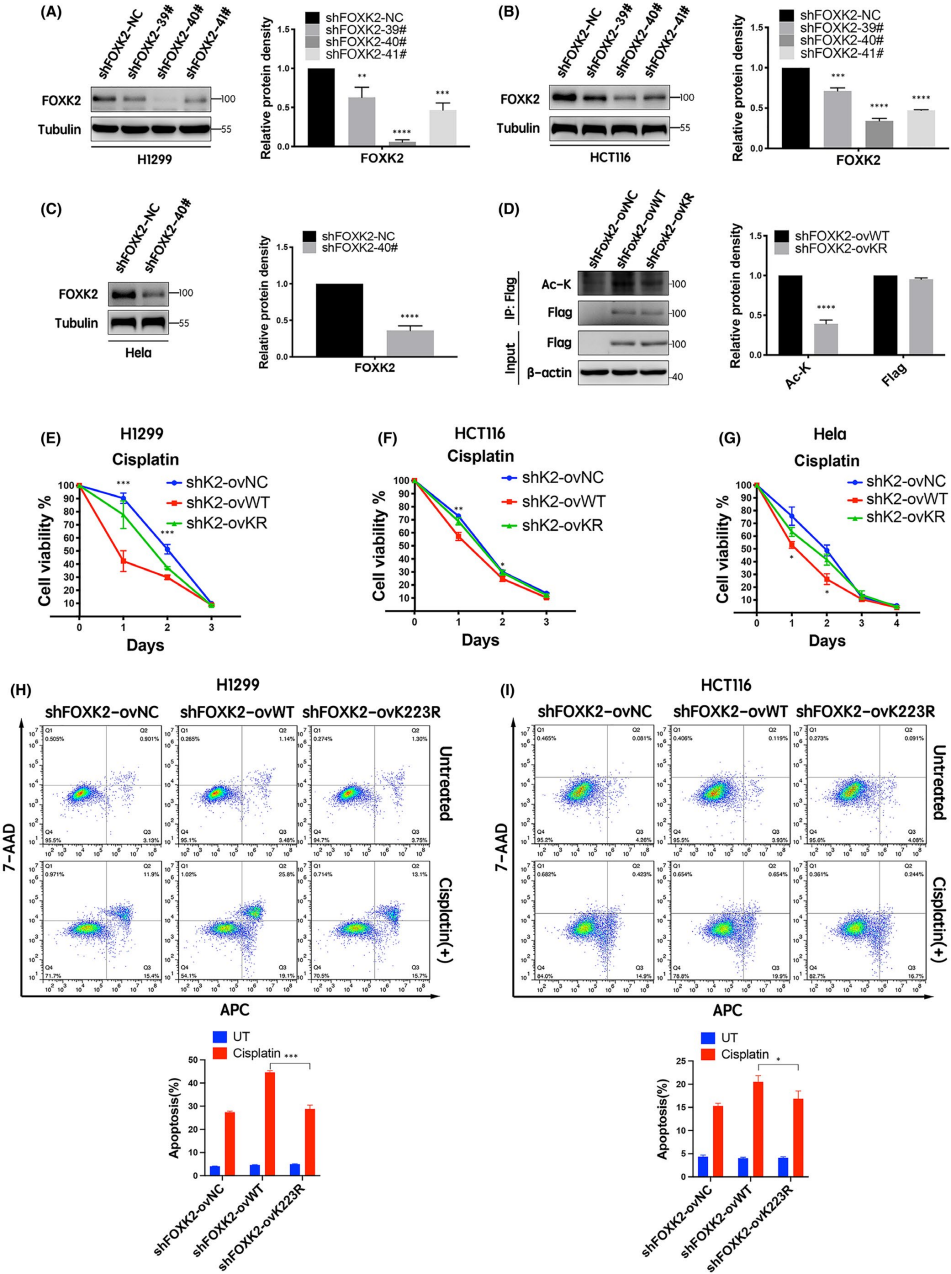
FOXK2 K223 deacetylation reduced the chemosensitivity of cancer cells in vitro. (A–C) NC, #39, #40 and #41 FOXK2‐RNAi were transfected into H1299 (A) and HCT116 (B) cells. NC and #40 FOXK2‐RNAi were transfected into HeLa cells (C). Western blotting was used to determine the knockdown efficiency of FOXK2. (D) ShFOXK2‐ovNC, shFOXK2‐ovWT and shFOXK2‐ovK223R H1299 cells were constructed. Immunoprecipitation and Western blotting were used to validate the acetylation level of FOXK2 in shFOXK2‐ovNC, shFOXK2‐ovWT and shFOXK2‐ovK223R cells. (E) ShFOXK2‐ovNC, shFOXK2‐ovWT and shFOXK2‐ovK223R H1299 cells were treated with cisplatin (15 μM, 3 days). (F) ShFOXK2‐ovNC, shFOXK2‐ovWT and shFOXK2‐ovK223R HCT116 cells were treated with cisplatin (2.5 μM, 3 days). (G) ShFOXK2‐ovNC, shFOXK2‐ovWT and shFOXK2‐ovK223R HeLa cells were treated with cisplatin (4 μM, 4 days). CCK‐8 assays were used to assess cytotoxicity. *p < 0.05, **p < 0.01, ***p < 0.001. (H) ShFOXK2‐ovNC, shFOXK2‐ovWT and shFOXK2‐ovK223R H1299 cells were cultured in normal medium or in the presence of cisplatin (15 μM) for 18 h. (I) ShFOXK2‐ovNC, shFOXK2‐ovWT and shFOXK2‐ovK223R HCT116 cells were cultured in normal medium or in the presence of cisplatin (20 μM) for 24 h. Three independent experiments were performed, and the data are presented as means ±standard errors of the means in a histogram format. Statistical analysis was performed using Mann‐Whitney tests. *p < 0.05, **p < 0.01, ***p < 0.001, ****p < 0.0001

Next, we further explored the effects of FOXK2 K223 acetylation on the chemosensitivity of cancer cells using stable transfection with shFOXK2‐ovNC, shFOXK2‐ovWT and shFOXK2‐ovK223R. The silencing effects of the constructs were confirmed, with shFOXK2‐40# showing the strongest FOXK2 knockdown in H1299, HCT116 and HeLa cells (Figure 2A–C). Cells were then transfected with Flag‐tagged vector, FOXK2‐WT or FOXK2‐K223R, yielding shFOXK2‐ovNC, shFOXK2‐ovWT and shFOXK2‐ovK223R cells. Immunoprecipitation and Western blotting showed that FOXK2 acetylation was significantly reduced in shFOXK2‐ovK223R cells compared with that in shFOXK2‐ovWT cells (Figure 2D).

To explore whether the FOXK2 acetylation status affected the chemosensitivity of cancer cells, shFOXK2‐ovNC, shFOXK2‐ovWT and shFOXK2‐ovK223R cells were treated with cisplatin or doxorubicin for 3–4 days. CCK‐8 assays then revealed that the viability of cells in the shFOXK2‐ovWT group was significantly lower than that in the shFOXK2‐ovNC and shFOXK2‐ovK223R groups following treatment with cisplatin (Figure 2E–G). Similar results were obtained following stimulation with doxorubicin (Figure S1B). All these differences were statistically significant.

In addition, we confirmed the effects of the three constructs on apoptosis induced by chemotherapeutic drugs (cisplatin, doxorubicin and etoposide) using flow cytometry analysis. The results revealed that the number of apoptotic cells was higher in the shFOXK2‐ovWT group than in the shFOXK2‐ovNC and shFOXK2‐ovK223R groups following treatment with cisplatin. There were no significant differences in the absence of cisplatin (Figure 2H,I). Similar results were obtained following treatment with doxorubicin and etoposide (Figure S1C,D).

**FIGURE 3:**
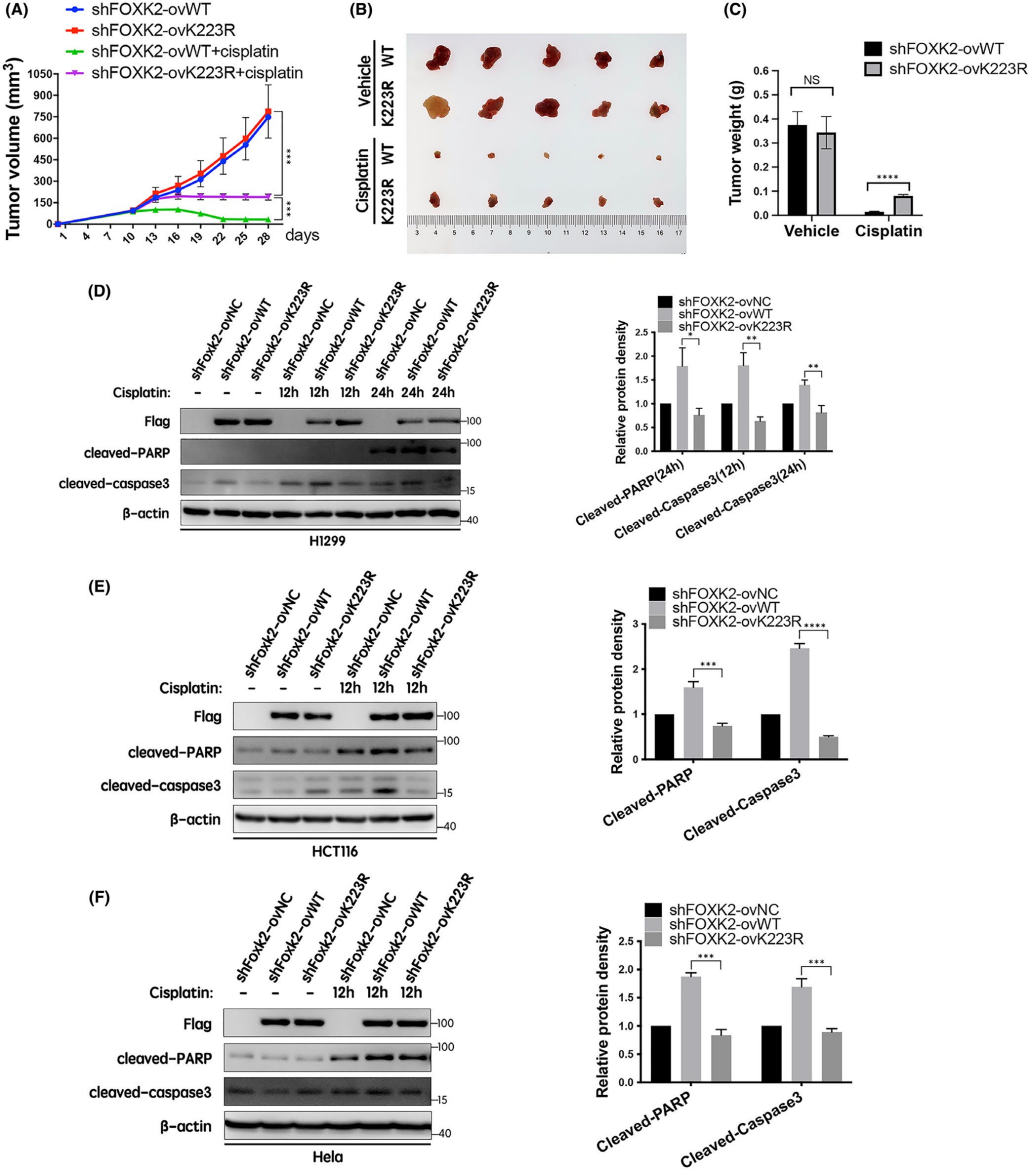
FOXK2 K223 deacetylation reduced the chemosensitivity of cancer cells in vivo by inhibiting cleaved‐PARP and cleaved‐caspase 3 expression. (A) Tumour growth curves for shFOXK2‐ovWT and shFOXK2‐ovK223R H1299 cells treated with vehicle or cisplatin. The formula L × W^2^ was used to calculate tumour volume. (B) The tumours were harvested on day 28; images from that day are shown. (C) Tumour weight (*n* = 5). Data points are means ±standard errors of the means. Statistical analyses were performed using unpaired Student's t tests. ***p < 0.001, ****p < 0.0001, NS: no significant difference. (D) ShFOXK2‐ovNC, shFOXK2‐ovWT and shFOXK2‐ovK223R H1299 cells were treated with cisplatin (15 μM) for 0, 12 or 24 h. (E) ShFOXK2‐ovNC, shFOXK2‐ovWT and shFOXK2‐ovK223R HCT116 cells were treated with cisplatin (10 μM) for 0 or 12 h. (F) ShFOXK2‐ovNC, shFOXK2‐ovWT and shFOXK2‐ovK223R HeLa cells were treated with cisplatin (7.5 μM) for 0 or 12 h. Expression levels of cleaved‐PARP and cleaved‐caspase 3 proteins were examined by Western blotting with the indicated antibodies (*n* = 3, each group). *p < 0.05, **p < 0.01, ***p < 0.001, ****p < 0.0001

Next, we evaluated the effects of FOXK2 acetylation on chemosensitivity in vivo by inoculation of shFOXK2‐ovWT and shFOXK2‐ovK223R H1299 cells into nude mice. As shown in Figure 3A, FOXK2‐K223R mutation did not affect tumour cell growth in the absence of cisplatin treatment compared with that in mice inoculated with FOXK2‐WT cells. However, shFOXK2‐ovK223R cells were less sensitive in the presence of cisplatin treatment in a xenograft model compared with shFOXK2‐ovWT cells. Furthermore, tumours in the shFOXK2‐ovWT group were smaller and weighed less than those in the FOXK2‐K223R group following cisplatin treatment (Figure 3B,C and Figure S2C).

Finally, to further verify the above results, the expression levels of specific apoptosis‐related proteins, including cleaved‐PARP and cleaved‐caspase3, were detected by Western blotting. The results showed that cleaved‐PARP and cleaved‐caspase 3 levels were increased in shFOXK2‐ovWT H1299, HCT116 and HeLa cells following cisplatin treatment. Furthermore, these two proteins were downregulated in shFOXK2‐ovNC and shFOXK2‐ovK223R cells compared with that in shFOXK2‐ovWT cells (Figure 3D–F). Consistent with this, similar results were also found in shFOXK2‐ovNC, shFOXK2‐ovWT and shFOXK2‐ovK223R H1299 and HCT116 cells treated with doxorubicin (Figure S2A,B). Collectively, these results indicated that FOXK2 K223 deacetylation reduced cancer cell chemosensitivity to cisplatin by inhibiting cleaved‐PARP and cleaved‐caspase 3 expression and suggested that the FOXK2 acetylation status may be a prognostic biomarker in cancer chemotherapy.

### 3.3 Acetylation of FOXK2 at K223 was determined by CBP and SIRT1

**FIGURE 4:**
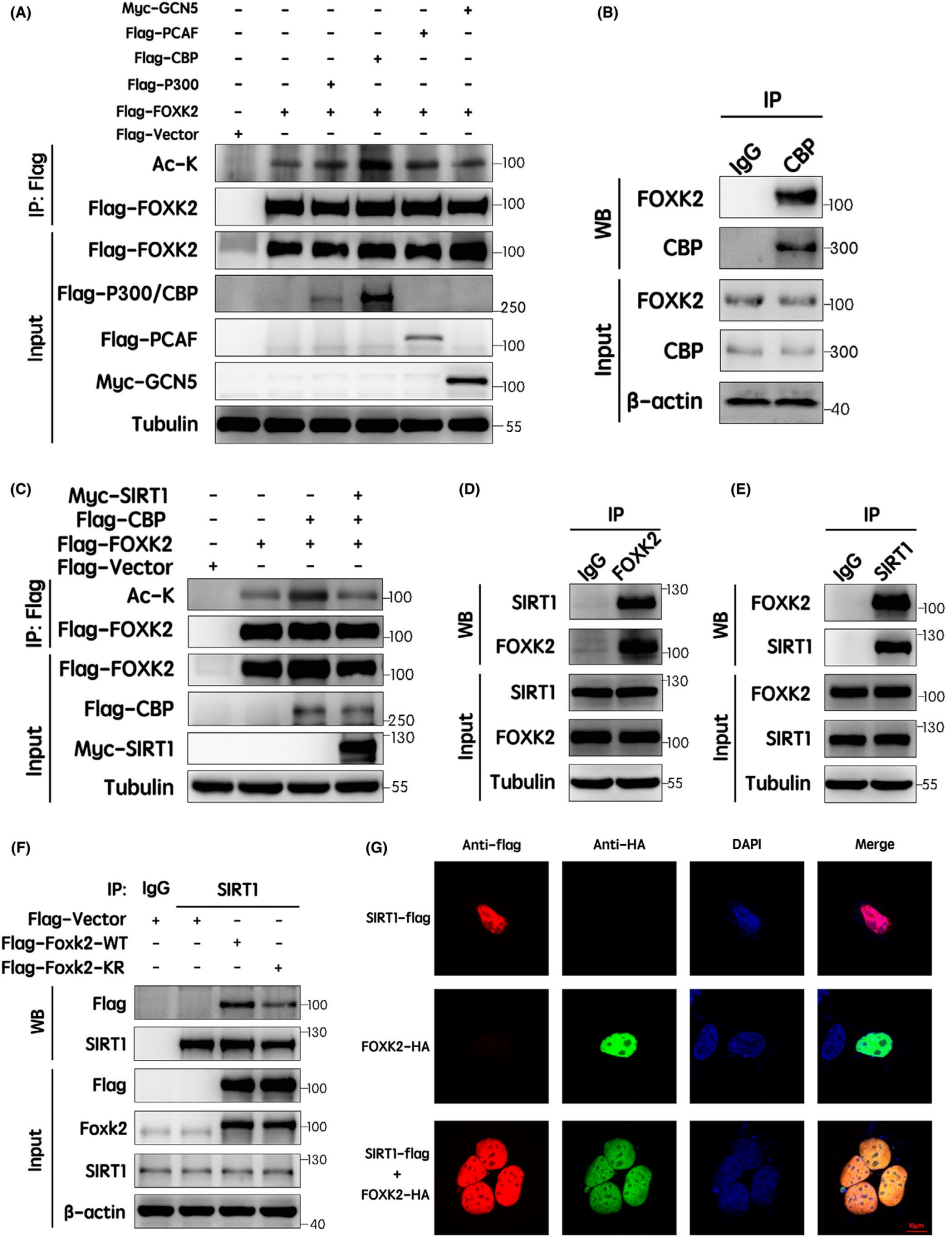
Acetylation of FOXK2 at K223 was determined by CBP and SIRT1. (A) Flag‐tagged FOXK2 was cotransfected with Flag‐tagged p300, Flag‐tagged CBP, Flag‐tagged PCAF or Myc‐tagged GCN5 into 293T cells. Flag‐FOXK2 was immunoprecipitated with an anti‐Flag antibody, and the acetylation level of FOXK2 was detected using an anti‐acetylated lysine antibody. (B) FOXK2 interacted with CBP in vivo. H1299 cell lysates were immunoprecipitated with control IgG or anti‐CBP antibodies, and Western blotting was performed using anti‐FOXK2 antibodies. After stripping the membrane, immunoprecipitated proteins were detected using anti‐CBP antibodies. (C) Flag‐tagged FOXK2, Flag‐tagged CBP and Myc‐tagged SIRT1 were cotransfected into 293T cells, and Flag‐FOXK2 was then immunoprecipitated with an anti‐Flag antibody. FOXK2 acetylation levels were detected using an anti‐acetylated lysine antibody. (D and E) FOXK2 interacted with SIRT1 in vivo. H1299 cell lysates were immunoprecipitated with control IgG, anti‐FOXK2 antibodies (D) or anti‐SIRT1 antibodies (E). The immunoprecipitated proteins were individually blotted with anti‐SIRT1 or anti‐FOXK2 antibodies, and anti‐FOXK2 or anti‐SIRT1 antibodies were used to detect the immunoprecipitated protein after stripping. (F) Flag‐tagged FOXK2 WT and K223R mutant were transfected into 293T cells, and immunoprecipitation was performed using IgG control or anti‐SIRT1 antibodies. Changes in interactions were detected by co‐immunoprecipitation and Western blotting. (G) Flag‐SIRT1, HA‐FOXK2, Flag‐SIRT1 and HA‐FOXK2 were transfected into HEK293 cells. Representative images of the colocalization of FOXK2 and SIRT1 were obtained using confocal laser scanning microscopy. Scale bar: 50 μm

To identify the acetyltransferase responsible for FOXK2, we cotransfected 293T cells with FOXK2 and each of the four acetyltransferases, that is E1A‐binding protein of 300 kDa (p300), CBP, PCAF and GCN5. Immunoprecipitation and Western blotting assays indicated that only the ectopic expression of CBP could dramatically increase the acetylation level of FOXK2 in 293T cells (Figure 4A). In HCT116 cells, CBP also significantly increased FOXK2 acetylation (Figure S1A). Therefore, we concluded that CBP was the acetyltransferase responsible for FOXK2 acetylation. Next, to confirm whether FOXK2 interacted with CBP in vivo, co‐immunoprecipitation and Western blotting were performed in H1299 cells. As shown in Figure 4B, endogenous FOXK2 co‐immunoprecipitated with endogenous CBP using anti‐CBP antibodies.

We then evaluated whether SIRT1 was the deacetylase responsible for FOXK2. To address this question, FOXK2 was cotransfected with CBP and SIRT1 in 293T cells. Immunoprecipitation and Western blotting assays indicated that overexpression of SIRT1 significantly attenuated FOXK2 acetylation, although CBP enhanced the acetylation level of FOXK2 (Figure 4C). Moreover, the interaction between endogenous FOXK2 and SIRT1 was confirmed by co‐immunoprecipitation in H1299 cells. Reciprocal analyses showed that FOXK2 interacted with SIRT1, and both could be endogenously combined into a protein complex in vivo (Figure 4D and E). To confirm that the acetylation modification was indeed removed by SIRT1, co‐immunoprecipitation between SIRT1 and FOXK2 WT or K223R mutant was performed. Consistent with our above findings, the results suggested that the interaction between SIRT1 and FOXK2 WT was stronger than that between SIRT1 and FOXK2 K223R mutant (Figure 4F). Subsequently, confocal microscopy was performed to verify the FOXK2/SIRT1 interaction and determine the localization of this interaction. HA‐tagged FOXK2 and Flag‐tagged SIRT1 were ectopically expressed in HEK293 cells. The results revealed that exogenous FOXK2 and SIRT1 colocalized in the nucleus (Figure 4G). Taken together, these results suggested that the acetylation of FOXK2 at K223 was determined by CBP and SIRT1.

### 3.4 Cisplatin and SIRT1 activity modulated the interactions, acetylation and nuclear distribution of FOXK2

In the context of DNA damage, SIRT1 deacetylates and activates FOXO proteins. The deacetylation state of FOXO proteins inhibits DNA damage‐induced apoptosis.^24^, ^28^ Our results confirmed that FOXK2 was deacetylated by SIRT1. Therefore, we hypothesized that SIRT1 may play important roles in regulating the interactions, acetylation levels and nuclear plasma distribution of FOXK2 to induce apoptosis in the context of DNA damage. To further characterize FOXK2 acetylation following cisplatin stimulation, several cellular biological mechanical assays were designed and performed.

**FIGURE 5:**
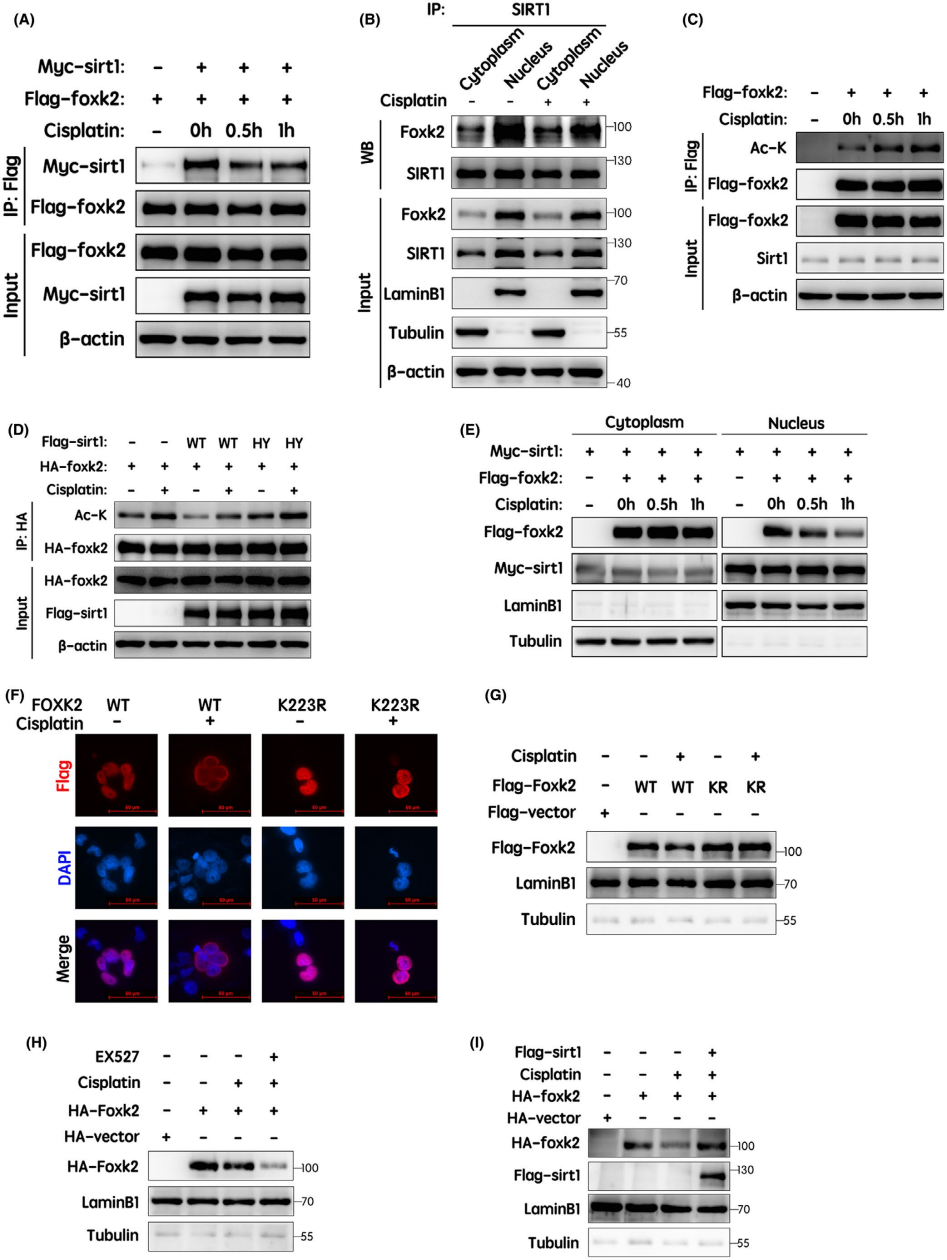
The interactions, acetylation levels and nuclear distribution of FOXK2 were regulated by cisplatin and SIRT1 enzymatic activity. (A) Flag‐tagged FOXK2 and Myc‐tagged SIRT1 were transfected into 293T cells. Flag‐tagged FOXK2 was immunoprecipitated using anti‐Flag antibodies in the absence or presence of cisplatin (15 μM) for 0.5 or 1 h. Changes in the interaction between FOXK2 and SIRT1 were evaluated by Western blotting. (B) After treatment with cisplatin (15 μM) for 1 h, nuclear and cytoplasmic proteins were extracted separately. Endogenous co‐immunoprecipitation assays were performed using anti‐SIRT1 antibodies. Changes in interactions in the nucleus and cytoplasm were identified using Western blotting. LaminB1, nuclear control; tubulin, cytoplasmic control. (C) 293T cells ectopically expressing Flag‐tagged FOXK2 were treated with cisplatin (15 μM) for 0.5 or 1 h. The acetylation level of exogenous FOXK2 was detected by immunoprecipitation and Western blotting. (D) HA‐tagged FOXK2 and Flag‐tagged SIRT1‐WT/H363Y (catalytically inactive mutant) were transfected into 293T cells with or without cisplatin treatment (15 μM) for 1 h. Changes in FOXK2 acetylation levels were determined by immunoprecipitation and Western blotting. (E) Flag‐tagged FOXK2 and Myc‐tagged SIRT1 were cotransfected into 293T cells. After 24 h, 293T cells were treated with cisplatin (15 μM) for 0.5 or 1 h, and the nuclear and cytoplasmic distributions of the indicated proteins were detected by Western blotting. LaminB1, nuclear control; tubulin, cytoplasmic control. (F) H1299 cells were transfected with Flag‐FOXK2 WT or K223R mutant. After 24 h, cells were treated with cisplatin (15 μM) for 1 h. Representative images are shown. Scale bar: 50 μm. (G) 293T cells were transfected with Flag‐FOXK2 WT or K223R mutant. After 24 h, 293T cells were treated with or without cisplatin (15 μM) for 1 h. Nuclear protein was then extracted, and the nuclear distribution of Flag‐tagged FOXK2 was detected by Western blotting. (H) 293T cells ectopically expressing HA‐tagged FOXK2 were treated with EX527 (0.5 μM, 9 h) in the presence of cisplatin (15 μM, 1 h). Nuclear protein was then extracted, and the nuclear distribution of HA‐tagged FOXK2 was detected by Western blotting. (I) HA‐tagged FOXK2 and Flag‐tagged SIRT1 were transfected into 293T cells with or without cisplatin treatment (15 μM) for 1 h. Nuclear protein was then extracted, and the nuclear distribution of HA‐tagged FOXK2 was detected by Western blotting. LaminB1, nuclear control; tubulin, cytoplasmic control

First, we evaluated whether the interaction between FOXK2 and SIRT1 was regulated by cisplatin stimulation. Interestingly, co‐immunoprecipitation and Western blotting revealed that the interaction between FOXK2 and SIRT1 was obviously attenuated by cisplatin stimulation (Figure 5A) and that this effect was observed in the nucleus, not the cytoplasm (Figure 5B).

Next, we hypothesized that FOXK2 acetylation may be enhanced by cisplatin stimulation. To test this hypothesis, we evaluated the acetylation levels of ectopically expressed FOXK2 following cisplatin stimulation. The results indicated that FOXK2 acetylation was significantly increased (Figure 5C), consistent with our hypothesis. In addition, to further establish whether the acetylation level of FOXK2 depended on the catalytic ability of SIRT1, 293T cells ectopically expressing HA‐tagged FOXK2 and Flag‐tagged SIRT1‐WT/H363Y (catalytically inactive mutant) were treated with or without cisplatin. The results showed that cisplatin increased FOXK2 acetylation levels, which could be reduced by wild‐type SIRT1, but not the H363Y mutants (Figure 5D). Thus, these findings suggested that SIRT1‐mediated FOXK2 deacetylation was dependent on SIRT1 enzymatic activity and that cisplatin treatment or SIRT1 inhibition enhanced FOXK2 acetylation levels.

To further explore the nuclear distribution of FOXK2 following cisplatin treatment, protein nucleoplasm separation assays and Western blotting were performed. As shown in Figure 5E, the nuclear distribution of ectopically expressed FOXK2 was dramatically reduced following stimulation with cisplatin. However, the distribution of ectopically expressed SIRT1 within the cytoplasm was almost constant. These findings suggested that the nuclear‐cytoplasmic distribution of FOXK2 may be regulated by cisplatin. To further identify whether the nuclear‐cytoplasmic distribution of FOXK2 was dependent on cisplatin‐mediated acetylation, the subcellular localization of wild‐type and K223R mutant FOXK2 following cisplatin treatment was examined by immunofluorescence analysis. Notably, the distribution of wild‐type FOXK2 in the nucleus was significantly reduced by cisplatin treatment, whereas that of the FOXK2‐K223R mutant was not (Figure 5F,G), and the reduced distribution of FOXK2‐K223D/E mutant (negatively charged residue) in the nucleus was not significant (Figure S2F). Furthermore, cisplatin treatment induced an obvious reduction in the nuclear distribution of HA‐tagged FOXK2, and the combination of cisplatin and EX527 further decreased nuclear HA‐tagged FOXK2 (Figure 5H). Moreover, ectopic expression of SIRT1 rescued the decreased nuclear distribution of FOXK2, which was induced by cisplatin stimulation (Figure 5I). Collectively, these results suggested that cisplatin treatment and SIRT1 inhibition reduced the nuclear distribution of FOXK2, which was induced by FOXK2 hyperacetylation. Thus, these findings established the molecular mechanisms through which SIRT1 deacetylated FOXK2 in the presence of cisplatin.

### 3.5 Role of FOXK2 K223 acetylation in the cell cycle and apoptosis in the presence of cisplatin

**FIGURE 6:**
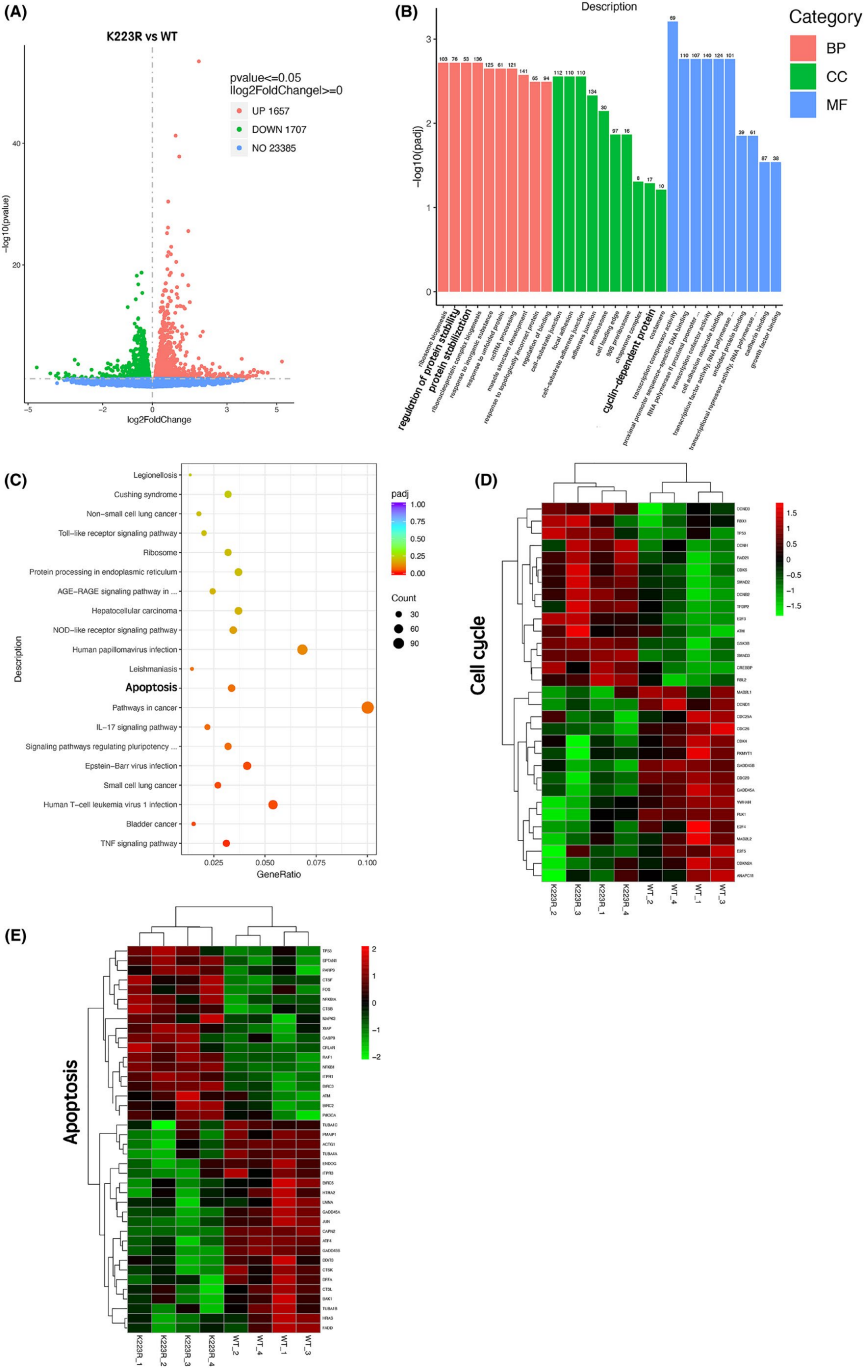
Role of FOXK2 K223 acetylation in the cell cycle and apoptosis in the presence of cisplatin. (A) Using RNA sequencing, a volcano graph was generated to show differentially expressed mRNAs in stable shFOXK2‐ovWT and shFOXK2‐ovK223R HeLa cells treated with cisplatin (15 μM) for 2 h. |log_2_ fold change| >0, *p* < 0.05. (B) GO analysis of differentially expressed mRNAs from stable shFOXK2‐ovWT and shFOXK2‐ovK223R HeLa cells treated with cisplatin (15 μM, 2 h). Adjusted *p* < 0.05. (C) KEGG pathways analysis of differentially expressed mRNAs in stable shFOXK2‐ovWT and shFOXK2‐ovK223R HeLa cells treated with cisplatin (15 μM, 2 h). Adjusted *p* < 0.05. (D) Heatmap of 31 representative cell cycle–related mRNAs. (E) Heatmap of 40 representative apoptosis‐related mRNAs

Next, we assessed the effects of cisplatin‐induced FOXK2 acetylation on downstream gene expression using RNA‐Seq. Biological duplicates were found to be similar using principal component analysis (Figure S2E). Volcano plots revealed 1657 upregulated mRNAs and 1707 downregulated mRNAs after screening with |log_2_ fold change| greater than 0 and *P* value less than 0.05 as the cut‐off criteria (Figure 6A and Figure S2D). A representative heat map (cut‐off criterion of |log_2_ fold change| > 1) is shown in Figure S1E.

**FIGURE 7:**
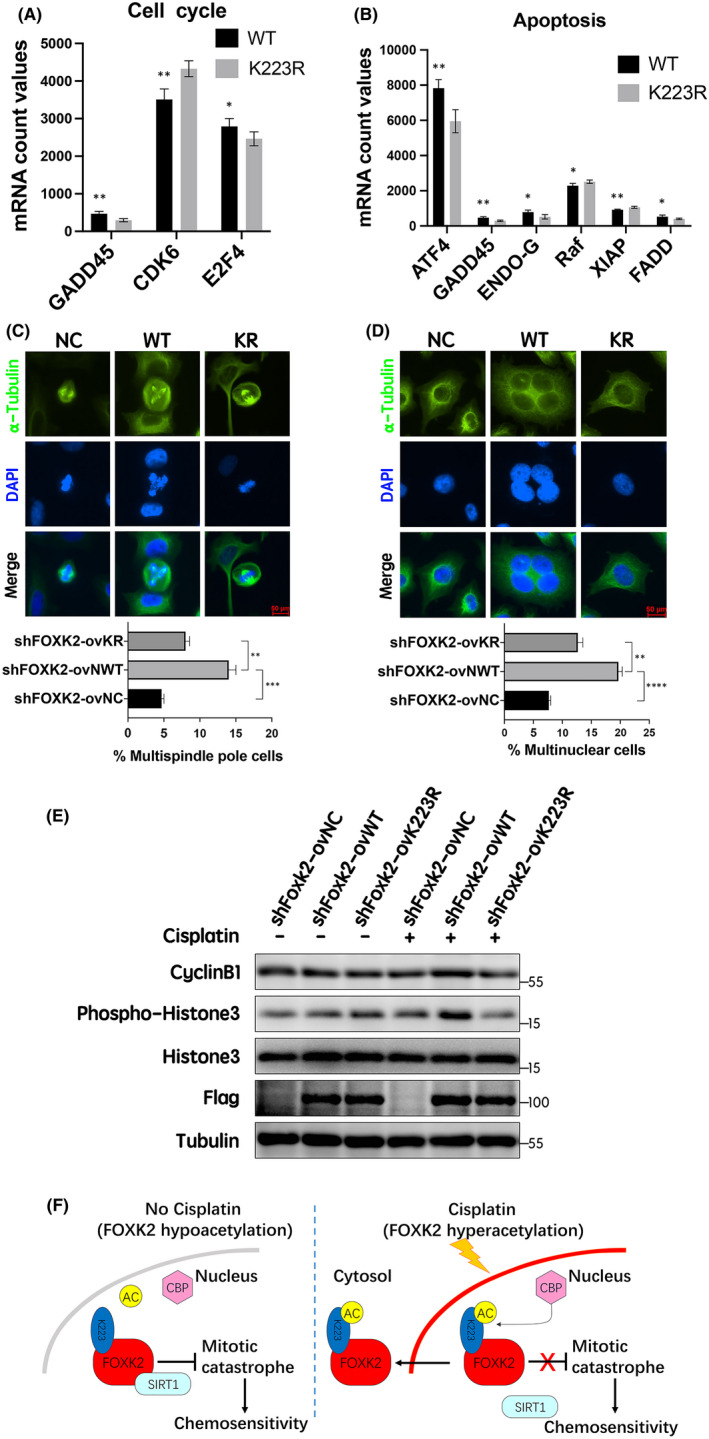
Role of FOXK2 K223 acetylation level in cell cycle and apoptosis in the presence of cisplatin. (A) Histogram of the expression of three indicated cell cycle–related mRNAs. (B) Histogram of the expression of six indicated apoptosis‐related mRNAs. (C) Representative spindle morphologies in metaphase HeLa cells after cisplatin treatment (15 μM, 2 h). α‐Tubulin is shown in green, and DAPI is shown in blue. (D) Representative nuclear morphologies in HeLa cells after cisplatin treatment (15 μM, 2 h). α‐Tubulin is shown in green, and DAPI is shown in blue. Scale bar: 50 μm. Data are presented as means ±standard errors of the means. **p < 0.01, ***p < 0.001, ****p < 0.0001. (E) Mitotic markers cyclin B1 and phospho‐H3 (Ser 10), as evaluated by Western blotting in shFOXK2‐ovNC, shFOXK2‐ovWT and shFOXK2‐ovK223R HeLa cells with or without cisplatin treatment (15 μM, 2 h). (F) A working model illustrating the mechanism of SIRT1‐mediated FOXK2 deacetylation and chemosensitivity to cisplatin

Additionally, Gene Ontology (GO) enrichment and Kyoto Encyclopedia of Genes and Genomes (KEGG) pathway analyses were performed to assess the potential biological functions affected by cisplatin‐induced FOXK2 acetylation. The top 10 biological process, cellular component and molecular function terms from GO analysis are presented in Figure 6B. GO terms of regulation of protein stability, protein stabilization and cyclin‐dependent protein were significantly enriched, possibly because of their relationship with apoptosis and the cell cycle. Furthermore, KEGG pathway analysis revealed enriched pathways for the differentially expressed mRNAs (File S1); 31 cell cycle–related mRNAs were significantly enriched in the cell cycle pathway (File S2), and 40 apoptosis‐related mRNAs were enriched in the apoptosis pathway (Figure 6C, File S3). To visualize these differentially expressed mRNAs affected by cisplatin‐induced FOXK2 acetylation, we depicted the expression levels of the 31 cell cycle–related mRNAs and 40 apoptosis‐related mRNAs using heatmaps (Figure 6D,E). In particular, FOXK2 hyperacetylation significantly increased the expression of *GADD45* and *E2F4* and decreased the expression of *CDK6*, all of which are involved in cell cycle arrest (Figure 7A and Figure S3A). In addition, FOXK2 hyperacetylation significantly increased the expression of *ATF4*, *GADD45*, *ENDO*‐*G* and FADD and decreased the expression of *Raf* and *XIAP*, which were generally thought to promote or inhibit apoptosis (Figure 7B and Figure S3B). Collectively, these results suggested that FOXK2 K223 acetylation levels obviously affected the expression of cell cycle–related and apoptosis‐related mRNAs in cancer cells following cisplatin stimulation. These findings provided insights into the mechanisms through which FOXK2 K223 deacetylation reduced chemosensitivity to cisplatin.

Mitotic catastrophe is a form of cell death that results from aberrant or failed mitosis. Cells undergoing mitotic catastrophe may be driven to an irreversible antiproliferative fate, leading to apoptosis.^31^ In our study, we found that hyperacetylation of FOXK2 at K223 following cisplatin treatment led to defective mitotic characteristics based on morphology, including multiple spindle poles (Figure 7C) and multiple nuclei (Figure 7D). Furthermore, FOXK2 hyperacetylation induced by cisplatin treatment obviously increased the levels of cyclinB1 and phospho‐histone H3 compared with FOXK2 hypoacetylation (Figure 7E). These results further suggested that cisplatin‐induced FOXK2 hyperacetylation at K223 promoted mitotic arrest and catastrophe. We speculate that differences in mitotic catastrophe may lead to the differences in apoptosis rates in cancer cells.

## 4 DISCUSSION

FOXK2 is involved in apoptosis in cancer cells. For example, FOXK2 promotes apoptosis in clear‐cell renal cell carcinoma through inhibition of epidermal growth factor receptor^2^ and plays critical roles in the control of apoptosis, metabolism and mitochondrial function following insulin stimulation.^32^ Furthermore, increasing evidence has shown that FOXK2 itself and PTM of FOXK2 are associated with apoptosis in cancer cells. FOXK2 is phosphorylated at Ser61 by checkpoint kinase 2 in the context of DNA damage; this traps FOXK2 in the cytoplasm, resulting in inhibition of apoptosis in cancer cells.^20^ Additionally, phosphorylation of FOXK2 at Ser368 and Ser423 by the CDK1/cyclinB complex inhibits apoptosis by regulating the stability of FOXK2 protein.^19^ SUMOylation of FOXK2 has also been shown to result in FOXK2‐induced apoptosis in breast cancer cells.^18^ However, no studies had previously evaluated the mechanisms and roles of FOXK2 acetylation. In this study, we identified FOXK2 as an acetylated protein and showed that SIRT1 mediated the deacetylation of FOXK2. We also demonstrated the molecular mechanisms through which FOXK2 deacetylation affected apoptosis in cancer cells treated with cisplatin.

It has been previously reported that in lung, prostate and colorectal cancer, the additive growth‐inhibitory effects of growth hormone‐releasing hormone (GHRH) antagonists plus cisplatin in vitro and in vivo are most likely due to the suppression by antagonist of PI3K/Akt activity. Induction of AKT is known to confer resistance to chemotherapeutic agents. Therefore, combined treatment with GHRH antagonists might increase chemosensitivity and hence the efficacy of cytotoxic substances, such as cisplatin.^33^, ^34^ Previous studies have also suggested that the chemosensitivity of cancer cells may be altered by PTM of proteins.^35^ In this study, we found that FOXK2 K223 hypoacetylation attenuated chemosensitivity to cisplatin. Moreover, following cisplatin treatment, the interaction between FOXK2 and SIRT1 was attenuated, thereby enhancing FOXK2 acetylation. We also showed that the nuclear distribution of FOXK2 was dependent on its acetylation status; cisplatin‐mediated FOXK2 hyperacetylation reduced its nuclear distribution. As a result, FOXK2 did not inhibit apoptosis in cancer cells in the nucleus following cisplatin treatment, consistent with previous studies.^20^ Furthermore, we demonstrated that FOXK2 K223 hyperacetylation promoted mitotic catastrophe. However, the nuclear distribution of the FOXK2 K223R mutant was not reduced following cisplatin treatment. This may be related to the inability of cisplatin to induce acetylation of the FOXK2 K223R mutant. Accordingly, hypoacetylated FOXK2 still localized in the nucleus and inhibited apoptosis in cancer cells, suggesting that FOXK2 K223 hypoacetylation may be involved in attenuation of chemosensitivity to cisplatin.

The function of SIRT1 in cancer development is complex and still not completely understood. Under stress conditions, SIRT1 blocks the activity of the tumour suppressor p53 and FOXO family proteins.^36^ In the context of DNA damage, FOXO3 deacetylation‐mediated apoptosis is also attenuated,^24^, ^37^ suggesting that SIRT1‐mediated deacetylation of FOXO proteins significantly reduces apoptosis in cancer cells. Consistent with previous studies, our results suggested that SIRT1‐mediated deacetylation of FOXK2 reduced apoptosis in cancer cells. Mechanistically, we demonstrated that the SIRT1 inhibitor EX527 enhanced FOXK2 acetylation in the presence or absence of cisplatin, thereby decreasing the nuclear distribution of FOXK2. Thus, EX527‐mediated FOXK2 hyperacetylation promoted mitotic catastrophe^38^ and chemosensitivity to cisplatin.

Multiple FOX family proteins are deacetylated by SIRT1, including FOXO1, FOXO3, FOXO4 and FOXO6.^37^, ^39^ In particular, the interaction between SIRT1 and FOXO3 is enhanced in the presence of oxidative stress.^24^ Our results revealed that cisplatin treatment may also suppress the interaction between SIRT1 and FOXK2, thereby enhancing the acetylation of FOXK2. Additionally, FOXO3 has previously been reported to translocate from the cytoplasm to the nucleus following stimulation with multiple stressors.^24^ We found that the nuclear distribution of FOXK2 was significantly reduced after cisplatin treatment. Taken together, these findings suggested that FOXK2 and FOXO3 may be affected by opposing SIRT1‐associated molecular mechanisms under stress conditions, although the effects on cancer cell apoptosis were similar.

Overall, our findings identified, for the first time, an acetylation‐dependent regulatory mechanism governing the role of FOXK2 in chemosensitivity to cisplatin. FOXK2 K223 hyperacetylation promoted apoptosis and enhanced chemosensitivity to cisplatin, whereas SIRT1‐mediated FOXK2 K223 deacetylation attenuated chemosensitivity to cisplatin. Moreover, our findings suggested that FOXK2 K223 acetylation status may have applications as a potential biomarker for chemotherapy outcomes.

## CONFLICT OF INTEREST

The authors declare that they have no conflicts of interest.

## AUTHOR CONTRIBUTIONS

**Xi‐wen Wang:** Investigation (lead); Methodology (lead); Resources (lead); Writing – original draft (lead); Writing – review & editing (lead). **Qi‐qiang Guo:** Methodology (equal). **Yang Yu:** Investigation (supporting). **Ting‐ting Zhou:** Investigation (supporting). **Si‐yi Zhang:** Investigation (supporting). **Zhuo Wang:** Methodology (equal). **Jing‐wei Liu:** Investigation (supporting). **Jun Tang:** Investigation (supporting). **Xiao‐you Jiang:** Investigation (supporting); Methodology (supporting). **Shan‐shan Wang:** Investigation (supporting). **Wen‐dong Guo:** Resources (supporting). **Hong‐de Xu:** Methodology (supporting). **Hua‐yi Sun:** Resources (supporting). **Zi‐wei Li:** Investigation (supporting). **Xiao‐yu Song:** Investigation (supporting); Methodology (supporting). **Jun‐gang Zhao:** Investigation (supporting); Resources (supporting). **Liu Cao:** Supervision (lead); Writing – original draft (supporting).

## Supporting information

Fig S1

Fig S2

Fig S3

Supinfo S1

Supinfo S2

Supinfo S3

## Data Availability

All data generated in this study are included within the article and its supplementary materials.
