# Supplementary figures and images for: The deacetylation of Foxk2 by Sirt1 reduces chemosensitivity to cisplatin
Source: J Cell Mol Med. 2021 Dec 6;26(2):491–506. doi: 10.1111/jcmm.17107 (PMC8743664; doi:10.1111/jcmm.17107)

**A**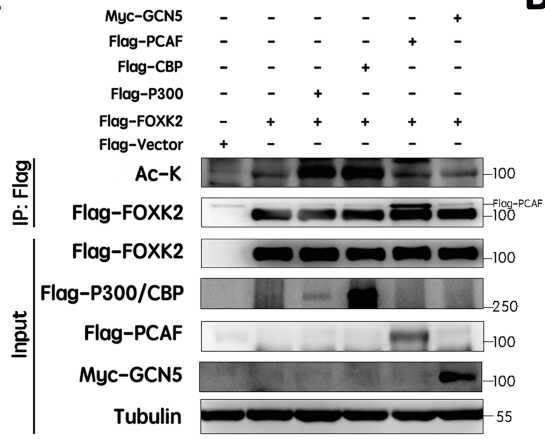**B**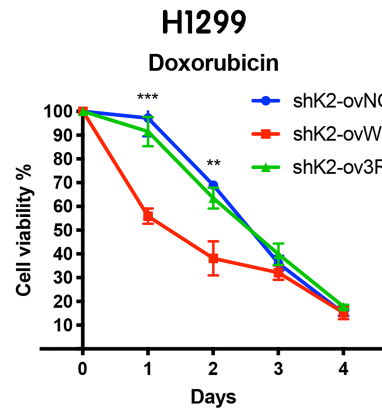**C**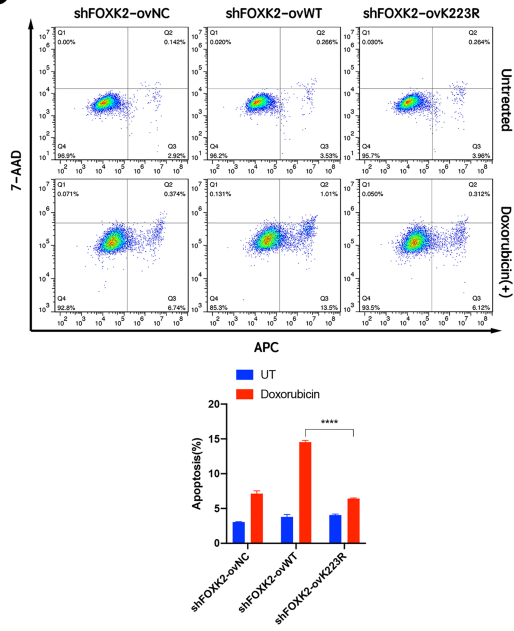**D**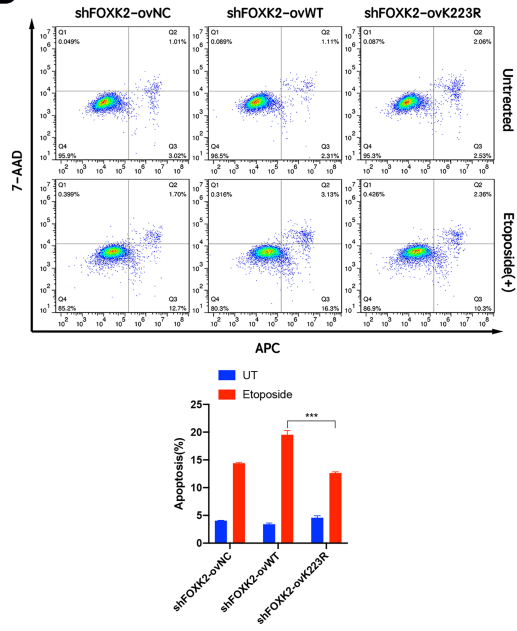**E**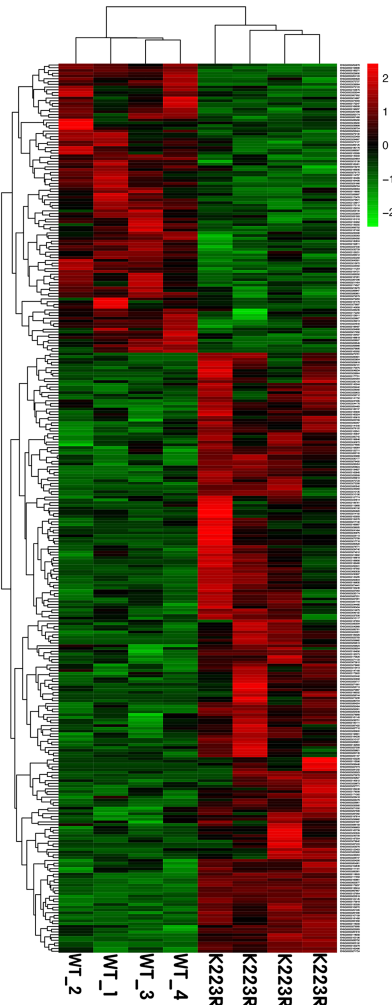

Supplement: Supplementary file 1 — Fig S1 [file JCMM-26-491-s003.pdf]

**A**

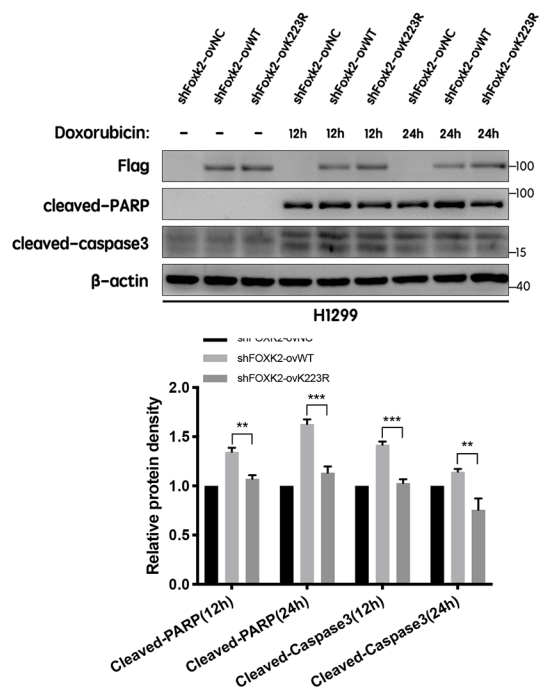

**B**

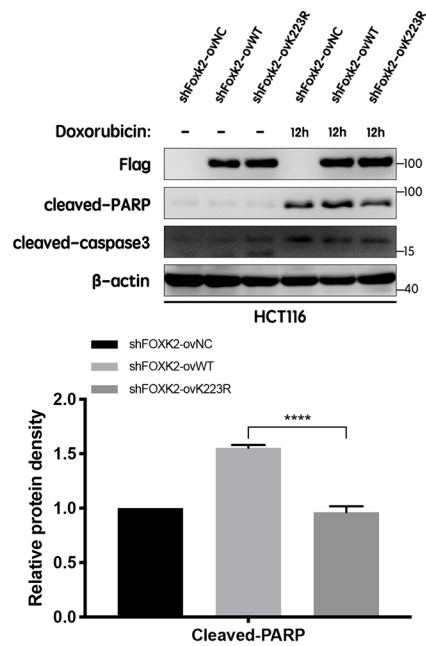

**C**

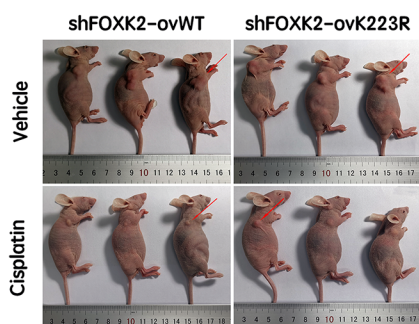

**D**

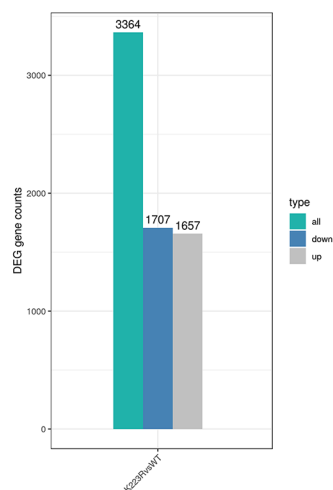

**E**

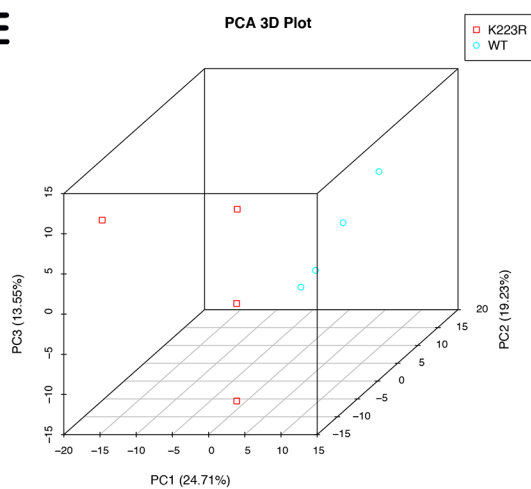

**F**

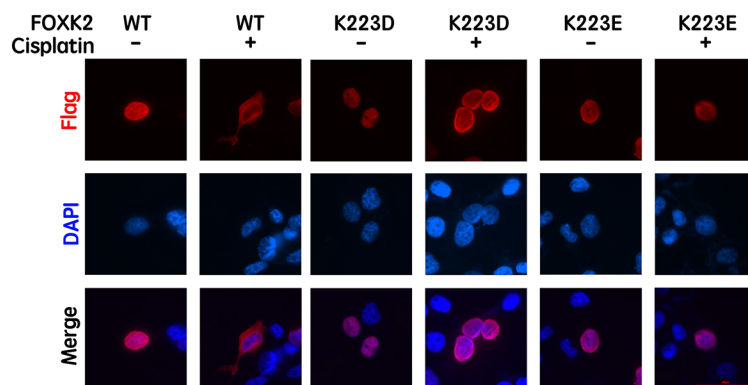

Supplement: Supplementary file 2 — Fig S2 [file JCMM-26-491-s006.pdf]
