# Supplementary material for: The deacetylation of Foxk2 by Sirt1 reduces chemosensitivity to cisplatin
Source: J Cell Mol Med. 2021 Dec 6;26(2):491–506. doi: 10.1111/jcmm.17107 (PMC8743664; doi:10.1111/jcmm.17107)
Supplement: Supplementary file 3 — Fig S3 [file JCMM-26-491-s001.pdf]

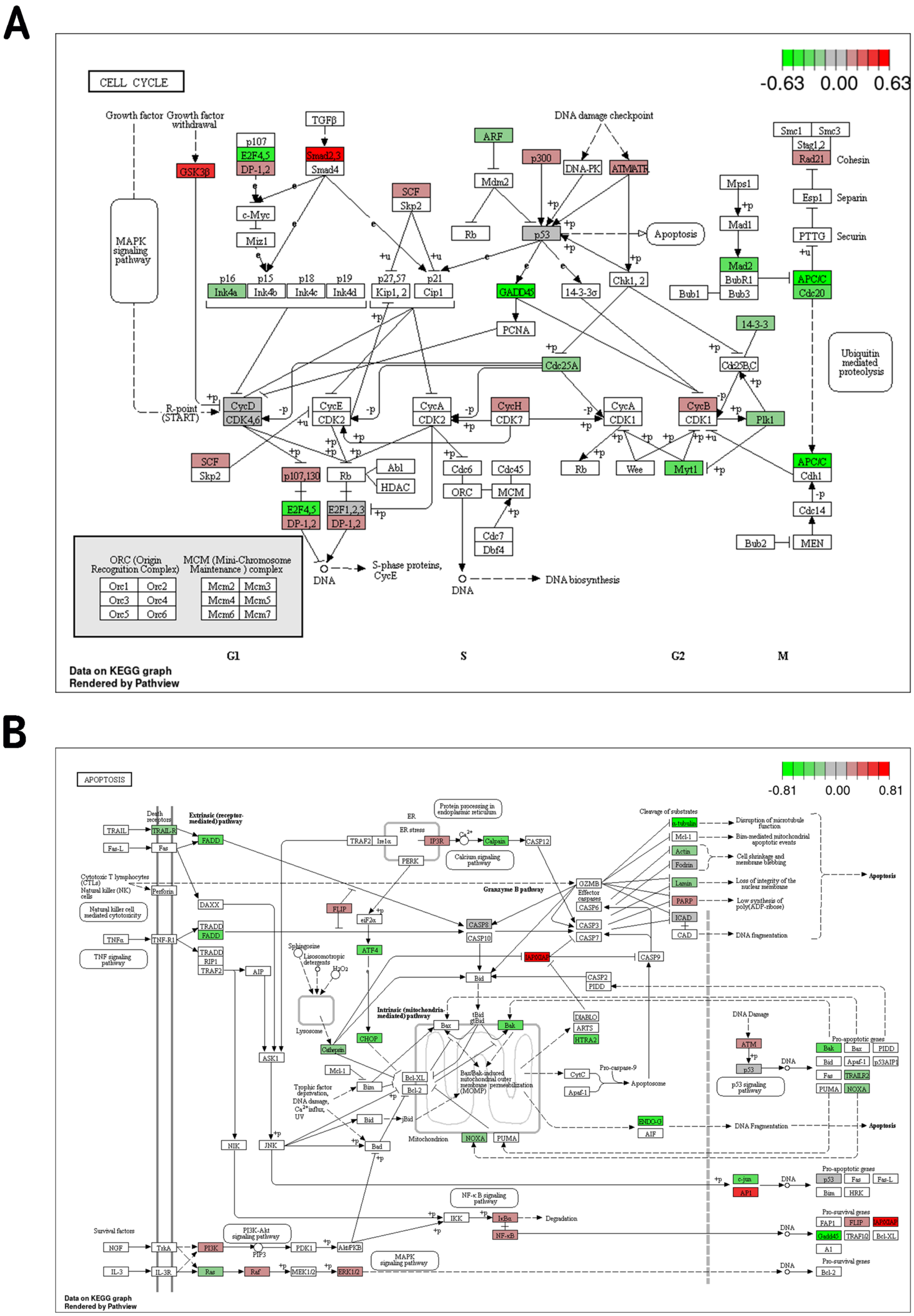

A
CELL CYCLE
-0.63 0.00 0.63
Growth factor
Growth factor withdrawal
TGFβ
Smad2,3
Smad4
p107
E2F4,5
DP-1,2
GSK3β
c-Myc
Miz1
MAPK signaling pathway
SCF
Skp2
ARF
Mdm2
p300
DNA damage checkpoint
DNA-PK
ATM/ATR
Rb
p53
Apoptosis
Smc1
Smc3
Stag1,2
Rad21
Cohesin
Esp1
Separin
PTTG
Securin
Mps1
Mad1
Mad2
BubR1
Bub3
Bub1
APC/C
Cdc20
p16
p15
p18
p19
Ink4a
Ink4b
Ink4c
Ink4d
p27,57
p21
Kip1, 2
Cip1
GADD45
14-3-3σ
Chk1, 2
14-3-3
PCNA
Ubiquitin mediated proteolysis
Cdc25A
Cdc25B,C
R-point (START)
CycD
CDK4,6
CycE
CDK2
CycA
CDK2
CycH
CDK7
CycA
CDK1
CycB
CDK1
Plk1
SCF
Skp2
p107,130
Rb
Abl
HDAC
Cdc6
Cdc45
Rb
Wee
Myt1
APC/C
Cdh1
E2F4,5
DP-1,2
E2F1,2,3
DP-1,2
ORC
MCM
Cdc14
Cdc7
Dbf4
Bub2
MEN
ORC (Origin Recognition Complex)
MCM (Mini-Chromosome Maintenance) complex
Orc1
Orc2
Orc3
Orc4
Orc5
Orc6
Mcm2
Mcm3
Mcm4
Mcm5
Mcm6
Mcm7
DNA
S-phase proteins, CycE
DNA
DNA biosynthesis
G1
S
G2
M
Data on KEGG graph
Rendered by Pathview
B
APOPTOSIS
-0.81 0.00 0.81
Death receptors
Extrinsic (receptor-mediated) pathway
TRAIL
TRAIL-R
Fas-L
Fas
FADD
Cytotoxic T lymphocytes (CTLs)
Natural killer (NK) cells
Perforin
Natural killer cell mediated cytotoxicity
DAXX
TNFα
TNF-R1
TRADD
FADD
TNF signaling pathway
TRADD
RIP1
TRAF2
AIP
ER
ER stress
Protein processing in endoplasmic reticulum
TRAF2
Ire1α
IP3R
Calpain
CASP12
PERK
Calcium signaling pathway
FLIP
eIF2α
ATF4
CHOP
Sphingosine
Lysosomotropic detergents
Lysosome
Cathepsin
GZMB
Granzyme B pathway
Effector caspases
CASP6
CASP3
CASP7
CASP8
CASP10
IAP/XIAP
CASP9
Bid
CASP2
PIDD
Cleavage of substrates
α-tubulin
Mcl-1
Actin
Fodrin
Lamin
PARP
ICAD
CAD
Disruption of microtubule function
Bim-mediated mitochondrial apoptotic events
Cell shrinkage and membrane blebbing
Loss of integrity of the nuclear membrane
Low synthesis of poly(ADP-ribose)
DNA fragmentation
Apoptosis
Intrinsic (mitochondria-mediated) pathway
Bax
tBid
Bak
DIABLO
ARTS
HTRA2
Bax/Bak-induced mitochondrial outer membrane permeabilization (MOMP)
CytC
Apaf-1
Pro-caspase-9
Apoptosome
Mcl-1
Bim
Bcl-XL
Bcl-2
Bid
jBid
Bad
Trophic factor deprivation, DNA damage, Ca2+ influx, UV
Mitochondrion
NOXA
PUMA
ENDO-G
AIF
DNA Fragmentation
ASK1
NIK
JNK
DNA Damage
ATM
p53
p53 signaling pathway
DNA
Pro-apoptotic genes
Bak
Bax
PIDD
Bid
Apaf-1
p53AIP1
Fas
TRAILR2
PUMA
NOXA
c-jun
AP1
p53
Fas
Fas-L
Bim
HRK
NF-κB signaling pathway
IKK
IκBα
NF-κB
Degradation
Pro-survival genes
FAP1
FLIP
IAP/XIAP
Gadd45
TRAF1/2
Bcl-XL
A1
Survival factors
NGF
TrkA
PI3K
PIP3
PI3K-Akt signaling pathway
PDK1
Akt/PKB
IL-3
IL-3R
Ras
Raf
MEK1/2
ERK1/2
MAPK signaling pathway
Bcl-2
Data on KEGG graph
Rendered by Pathview
