# Supplementary material for: The deacetylation of Foxk2 by Sirt1 reduces chemosensitivity to cisplatin
Source: J Cell Mol Med. 2021 Dec 6;26(2):491–506. doi: 10.1111/jcmm.17107 (PMC8743664; doi:10.1111/jcmm.17107)
Supplement: Supplementary file 4 — Supinfo S1 [file JCMM-26-491-s004.pdf]

The most enriched pathway terms

Statistic method: hypergeometric test

FDR correction method: Benjamini and Hochberg

Term	Description	Sample number	Background number	P-value	Corrected P-value	Gene_id	Gene_nan
hsa04668	TNF signaling pathway	37/1188	94/5573	4.67047556586705e-05	0.0143383599872118	ENSG00000136244 ENSG00000118503 ENSG00000108984 ENSG00000023445 ENSG00000128272 ENSG00000115009 ENSG00000069399 ENSG00000162302 ENSG00000172216 ENSG00000109320 ENSG00000182158 ENSG00000177606 ENSG00000073756 ENSG00000110330 ENSG00000168404 ENSG00000090339 ENSG00000100906 ENSG00000131323 ENSG00000163739 ENSG00000168040 ENSG00000184557 ENSG00000100784 ENSG00000157625 ENSG00000185386 ENSG00000128342 ENSG00000102882 ENSG00000188130 ENSG00000003402 ENSG00000170345 ENSG00000184371 ENSG00000247077 ENSG00000164136 ENSG00000121879 ENSG00000078747 ENSG00000065559 ENSG00000100324 ENSG00000064012	IL6 TNFAIP MAP2K6 BIRC3 ATF CCL20 BCL RPS6KA4 CEBPB NFKI CREB3L2 JU PTGS2 BIRC MLKL ICAM NFKBIA TRAF3 CXCI FADD SOCS RPS6KA5 TAB3 MAPK LIF MAPK3 MAPK12 CFLAR FOS CSF1 PGAM IL15 PIK3C ITCH MAP2I TAB1 CASP
hsa05219	Bladder cancer	18/1188	38/5573	0.00030111143160324	0.0365222017434792	ENSG00000137801 ENSG00000169429 ENSG00000196730 ENSG00000068028 ENSG00000110092 ENSG00000132155 ENSG00000135446 ENSG00000174775 ENSG00000068078 ENSG00000100784 ENSG00000147889 ENSG00000197122 ENSG00000102882 ENSG00000025708 ENSG00000112715 ENSG00000112242 ENSG00000157764 ENSG00000141510	THBS1 CXCI DAPK1 RASSF1 CCND1 RAF CDK4 HRA FGFR3 RPS6KA5 CDKN2A SR MAPK3 TYM VEGFA E2F BRAF TP5
hsa05166	Human T-cell leukemia virus 1 infection	64/1188	205/5573	0.000475214435277415	0.0365222017434792	ENSG00000136244 ENSG00000140577 ENSG00000077150 ENSG00000162772 ENSG00000082701 ENSG00000128272 ENSG00000114251 ENSG00000104856 ENSG00000117399	IL6 CRTC3 NFKB2 ATF GSK3B ATF WNT5A REL CDC20 SMA KAT2A DVL NFKB1 JAK CCND1 WNT7B

						ENSG00000166949<br>ENSG00000108773<br>ENSG00000107404<br>ENSG00000109320<br>ENSG00000162434<br>ENSG00000110092<br>ENSG00000188064<br>ENSG00000112576<br>ENSG00000177606<br>ENSG00000115350<br>ENSG00000175592<br>ENSG00000175387<br>ENSG00000155760<br>ENSG00000090339<br>ENSG00000179218<br>ENSG00000100906<br>ENSG00000148229<br>ENSG00000005339<br>ENSG00000132341<br>ENSG00000176386<br>ENSG00000157557<br>ENSG00000164362<br>ENSG00000101966<br>ENSG00000135446<br>ENSG00000174775<br>ENSG00000174233<br>ENSG00000147889<br>ENSG00000180340<br>ENSG00000071564<br>ENSG00000163132<br>ENSG00000075711<br>ENSG00000157456<br>ENSG00000162104<br>ENSG00000169884<br>ENSG00000170345<br>ENSG00000095794<br>ENSG00000100385<br>ENSG00000115596<br>ENSG00000163251<br>ENSG00000149311<br>ENSG00000106628<br>ENSG00000117394<br>ENSG00000141552<br>ENSG00000164136<br>ENSG00000121879<br>ENSG00000157240<br>ENSG00000121281<br>ENSG00000123268<br>ENSG00000065559<br>ENSG00000164109<br>ENSG00000112242<br>ENSG00000126561<br>ENSG00000062822<br>ENSG00000147168<br>ENSG00000141510	CCND3 JU<br>POLE4 FOS<br>SMAD2 FZD<br>ICAM1 CAL<br>NFKBIA<br>POLE3<br>CREBBP RA<br>CDC26 ETS<br>TERT XIA<br>CDK4 HRA<br>ADCY6<br>CDKN2A FZ<br>TCF3 MSX<br>DLG1 CCN<br>ADCY9<br>WNT10B F<br>CREM IL2R<br>WNT6 FZD<br>ATM POLD<br>SLC2A1<br>ANAPC11<br>IL15 PIK3C<br>FZD1 ADCY<br>ATF1 MAP2<br>MAD2L1 E2<br>STAT5A<br>POLD1 IL2R<br>TP53
hsa05222	Small cell lung cancer	32/1188	86/5573	0.000497862951348489	0.0365222017434792	ENSG00000023445<br>ENSG00000196878<br>ENSG00000116717<br>ENSG00000109320<br>ENSG00000172037<br>ENSG00000105810<br>ENSG00000110092<br>ENSG00000173207<br>ENSG00000099860<br>ENSG00000073756<br>ENSG00000110330<br>ENSG00000134574<br>ENSG00000115414<br>ENSG00000130702<br>ENSG00000100906<br>ENSG00000131323<br>ENSG00000101966<br>ENSG00000188153<br>ENSG00000135446<br>ENSG00000030110<br>ENSG00000076604<br>ENSG00000123975<br>ENSG00000164171<br>ENSG00000197565<br>ENSG00000101680<br>ENSG00000171862<br>ENSG00000121879	BIRC3 LAM<br>GADD45A<br>NFKB1 LAM<br>CDK6 CCN<br>CKS1B<br>GADD45B<br>PTGS2 BIR<br>DDB2 FN1<br>LAMA5<br>NFKBIA<br>TRAF3 XIA<br>COL4A5 CD<br>BAK1 TRAF<br>CKS2 ITGA<br>COL4A6<br>LAMA1 PTE<br>PIK3CA<br>LAMC1 E2F<br>ITGA3 LAM<br>TP53

						ENSG00000135862<br>ENSG00000112242<br>ENSG00000005884<br>ENSG00000050555<br>ENSG00000141510	
hsa05169	Epstein-Barr virus infection	49/1188	149/5573	0.000594824132629954	0.0365222017434792	ENSG00000136244<br>ENSG00000118503<br>ENSG00000077150<br>ENSG00000108984<br>ENSG00000116717<br>ENSG00000104856<br>ENSG00000109320<br>ENSG00000105810<br>ENSG00000020633<br>ENSG00000162434<br>ENSG00000110092<br>ENSG00000112576<br>ENSG00000177606<br>ENSG00000146232<br>ENSG00000099860<br>ENSG00000134574<br>ENSG00000169375<br>ENSG00000142166<br>ENSG00000090339<br>ENSG00000179218<br>ENSG00000100906<br>ENSG00000167004<br>ENSG00000184216<br>ENSG00000131323<br>ENSG00000135446<br>ENSG00000168040<br>ENSG00000030110<br>ENSG00000108344<br>ENSG00000185386<br>ENSG00000188130<br>ENSG00000187608<br>ENSG00000115415<br>ENSG00000111335<br>ENSG00000100519<br>ENSG00000197943<br>ENSG00000175166<br>ENSG00000121879<br>ENSG00000013275<br>ENSG00000065559<br>ENSG00000104825<br>ENSG00000263528<br>ENSG00000112242<br>ENSG00000164105<br>ENSG00000100324<br>ENSG00000138185<br>ENSG00000026025<br>ENSG00000138433<br>ENSG00000064012<br>ENSG00000141510	IL6 TNFAIP<br>NFKB2<br>MAP2K6<br>GADD45A<br>RELB NFKB<br>CDK6 RUNX<br>JAK1 CCND<br>CCND3 JUN<br>NFKBIE<br>GADD45B<br>DDB2 SIN3<br>IFNAR1<br>ICAM1 CAL<br>NFKBIA PDI<br>IRAK1 TRAF<br>CDK4 FADD<br>BAK1 PSMD<br>MAPK11<br>MAPK12<br>ISG15 STAT<br>OAS2 PSMC<br>PLCG2 PSMD<br>PIK3CA<br>PSMC4<br>MAP2K4<br>NFKBIB IKBK<br>E2F3 SAP3<br>TAB1 ENTPD<br>VIM CIR1<br>CASP8 TP5
hsa04550	Signaling pathways regulating pluripotency of stem cells	38/1188	113/5573	0.00149734778478924	0.0736741054270069	ENSG00000135111<br>ENSG00000082701<br>ENSG00000114251<br>ENSG00000166949<br>ENSG00000139269<br>ENSG00000107404<br>ENSG00000162434<br>ENSG00000188064<br>ENSG00000140836<br>ENSG00000175387<br>ENSG00000155760<br>ENSG00000008083<br>ENSG00000160867<br>ENSG00000132155<br>ENSG00000174775<br>ENSG00000117318<br>ENSG00000068078<br>ENSG00000115507<br>ENSG00000180340<br>ENSG00000071564<br>ENSG00000185386<br>ENSG00000128342<br>ENSG00000168283<br>ENSG00000102882<br>ENSG00000188130<br>ENSG00000103126<br>ENSG00000169884<br>ENSG00000115596	TBX3 GSK3<br>WNT5A<br>SMAD3 INHB<br>DVL1 JAK1<br>WNT7B<br>ZFHX3 SMAD<br>FZD7 JARID<br>FGFR4 RAF<br>HRAS ID3<br>FGFR3 OTX<br>FZD2 TCF7<br>MAPK11 LI<br>BMI1 MAPK<br>MAPK12<br>AXIN1<br>WNT10B<br>WNT6 DUSP<br>FZD5 ID2<br>KAT6A<br>BMPR1A<br>PIK3CA FZD<br>ID1 ACVR1<br>PCGF6

						ENSG00000130829 ENSG00000163251 ENSG00000115738 ENSG00000083168 ENSG00000107779 ENSG00000121879 ENSG00000157240 ENSG00000125968 ENSG00000135503 ENSG00000156374	
[hsa04657]()	IL-17 signaling pathway	26/1188	70/5573	0.00167986559605553	0.0736741054270069	ENSG00000136244 ENSG00000118503 ENSG00000169429 ENSG00000082701 ENSG00000115009 ENSG00000080824 ENSG00000172216 ENSG00000109320 ENSG00000166598 ENSG00000177606 ENSG00000175592 ENSG00000073756 ENSG00000100906 ENSG00000131323 ENSG00000163739 ENSG00000168040 ENSG00000076604 ENSG00000096384 ENSG00000157625 ENSG00000177663 ENSG00000185386 ENSG00000102882 ENSG00000188130 ENSG00000170345 ENSG00000263528 ENSG00000064012	IL6 TNFAIP CXCL8 GSK CCL20 HSP90AA1 CEBPB NFK HSP90B1 JU FOSL1 PTG NFKBIA TRAF3 CXC FADD TRAF HSP90AB1 TAB3 IL17R MAPK11 MAPK3 MAPK12 FC IKBKE CAS
[hsa05200]()	Pathways in cancer	119/1188	442/5573	0.00203231897069997	0.0779902405006112	ENSG00000136244 ENSG00000077150 ENSG00000023445 ENSG00000196878 ENSG00000169429 ENSG00000082701 ENSG00000196730 ENSG00000114251 ENSG00000116717 ENSG00000173801 ENSG00000080824 ENSG00000184916 ENSG00000166949 ENSG00000107404 ENSG00000109320 ENSG00000172037 ENSG00000134318 ENSG00000105976 ENSG00000105810 ENSG00000162434 ENSG00000068028 ENSG00000110092 ENSG00000149782 ENSG00000188064 ENSG00000112576 ENSG00000166598 ENSG00000177606 ENSG00000173207 ENSG00000099860 ENSG00000137193 ENSG00000073756 ENSG00000110330 ENSG00000134574 ENSG00000175387 ENSG00000155760 ENSG00000142166 ENSG00000116016 ENSG00000168811 ENSG00000115414 ENSG00000027697 ENSG00000130702 ENSG00000100906 ENSG00000128917 ENSG00000148400 ENSG00000143198 ENSG00000005339	IL6 NFKB2 BIRC3 LAM CXCL8 GSK DAPK1 WNT5A GADD45A JU HSP90AA1 JAG2 SMAD DVL1 NFKB LAMB2 ROCK2 ME CDK6 JAK RASSF1 CCND1 PLC WNT7B CCND3 HSP90B1 JU CKS1B GADD45B PIM1 PTGS BIRC2 DDB SMAD2 FZD IFNAR1 EPAS1 IL12 FN1 IFNGR LAMA5 NFKBIA DL NOTCH1 MGST3 CREBBP FGFR4 RUN RAF1 TRAF TERT XIA COL4A5 RA CDK4 BMP HRAS FAD BAK1 TRAF SOS2 FGFR ADCY6 HSP90AB1 RPS6KA5 CDKN2A ST TCF7L2 CK MGST2 FZD MITF EGLN PAX8 LRP

						ENSG00000160867<br>ENSG00000159216<br>ENSG00000132155<br>ENSG00000131323<br>ENSG00000164362<br>ENSG00000101966<br>ENSG00000188153<br>ENSG00000144118<br>ENSG00000135446<br>ENSG00000125845<br>ENSG00000174775<br>ENSG00000168040<br>ENSG00000030110<br>ENSG00000076604<br>ENSG00000100485<br>ENSG00000068078<br>ENSG00000174233<br>ENSG00000096384<br>ENSG00000100784<br>ENSG00000147889<br>ENSG00000101109<br>ENSG00000148737<br>ENSG00000123975<br>ENSG00000085871<br>ENSG00000180340<br>ENSG00000187098<br>ENSG00000135766<br>ENSG00000125618<br>ENSG00000162337<br>ENSG00000172354<br>ENSG00000102882<br>ENSG00000141682<br>ENSG00000103126<br>ENSG00000173511<br>ENSG00000162104<br>ENSG00000116044<br>ENSG00000171680<br>ENSG00000169884<br>ENSG00000112715<br>ENSG00000170345<br>ENSG00000100644<br>ENSG00000115415<br>ENSG00000100385<br>ENSG00000115596<br>ENSG00000197943<br>ENSG00000163251<br>ENSG00000134184<br>ENSG00000164171<br>ENSG00000117394<br>ENSG00000089685<br>ENSG00000163235<br>ENSG00000196914<br>ENSG00000197565<br>ENSG00000101680<br>ENSG00000124151<br>ENSG00000171862<br>ENSG00000164136<br>ENSG00000121879<br>ENSG00000157240<br>ENSG00000121281<br>ENSG00000100387<br>ENSG00000135862<br>ENSG00000112242<br>ENSG00000126561<br>ENSG00000157764<br>ENSG00000005884<br>ENSG00000164683<br>ENSG00000198431<br>ENSG00000147168<br>ENSG00000050555<br>ENSG00000102096<br>ENSG00000064012<br>ENSG00000141510	GNB2 MAP<br>PMAIP1 AXI<br>VEGFB ADC<br>NFE2L2<br>PLEKHG5<br>WNT10B<br>VEGFA FO<br>HIF1A STA<br>IL2RB WNT<br>PLCG2 FZD<br>GSTM1 ITG<br>SLC2A1 BIR<br>TGFA<br>ARHGEF1<br>COL4A6<br>LAMA1<br>NCOA3 PTE<br>IL15 PIK3C<br>FZD1 ADCY<br>RBX1 LAMC<br>E2F3 STAT<br>BRAF ITGA<br>HEY1 TXNR<br>IL2RG LAM<br>PIM2 CASP<br>TP53
[hsa04210]()	Apoptosis	40/1188	123/5573	0.00231653754838153	0.0790196697059033	ENSG00000162909<br>ENSG00000150995<br>ENSG00000023445<br>ENSG00000128272<br>ENSG00000116717<br>ENSG00000109320<br>ENSG00000167136<br>ENSG00000177606<br>ENSG00000127824	CAPN2 ITP<br>BIRC3 ATF<br>GADD45A<br>NFKB1<br>ENDOG JU<br>TUBA4A<br>ACTG1<br>GADD45B<br>BIRC2 CTS

						ENSG00000184009<br>ENSG00000099860<br>ENSG00000110330<br>ENSG00000174080<br>ENSG00000164733<br>ENSG00000100906<br>ENSG00000132155<br>ENSG00000101966<br>ENSG00000115317<br>ENSG00000174775<br>ENSG00000168040<br>ENSG00000030110<br>ENSG00000197694<br>ENSG00000123416<br>ENSG00000175197<br>ENSG00000096433<br>ENSG00000102882<br>ENSG00000141682<br>ENSG00000003402<br>ENSG00000170345<br>ENSG00000160049<br>ENSG00000149311<br>ENSG00000135047<br>ENSG00000167553<br>ENSG00000089685<br>ENSG00000160789<br>ENSG00000121879<br>ENSG00000041880<br>ENSG00000143387<br>ENSG00000064012<br>ENSG00000141510	CTSB NFKB<br>RAF1 XIAF<br>HTRA2 HRA<br>FADD BAK<br>SPTAN1<br>TUBA1B<br>DDIT3 ITPR<br>MAPK3<br>PMAIP1<br>CFLAR FOS<br>DFFA ATM<br>CTSL TUBA1<br>BIRC5 LMN<br>PIK3CA<br>PARP3 CTS<br>CASP8 TP5
hsa05140	Leishmaniasis	17/1188	41/5573	0.00274679630476603	0.0843266465563171	ENSG00000125730<br>ENSG00000109320<br>ENSG00000162434<br>ENSG00000177606<br>ENSG00000073756<br>ENSG00000168811<br>ENSG00000027697<br>ENSG00000100906<br>ENSG00000184216<br>ENSG00000185386<br>ENSG00000102882<br>ENSG00000188130<br>ENSG00000170345<br>ENSG00000115415<br>ENSG00000116701<br>ENSG00000104825<br>ENSG00000100324	C3 NFKB1<br>JAK1 JUN<br>PTGS2 IL12<br>IFNGR1<br>NFKBIA IRA<br>MAPK11<br>MAPK3<br>MAPK12 FC<br>STAT1 NCF<br>NFKBIB TAB
hsa05165	Human papillomavirus infection	81/1188	291/5573	0.00407589831362631	0.113754616571207	ENSG00000105855<br>ENSG00000196878<br>ENSG00000137801<br>ENSG00000082701<br>ENSG00000114251<br>ENSG00000109072<br>ENSG00000135114<br>ENSG00000107404<br>ENSG00000108821<br>ENSG00000109320<br>ENSG00000172037<br>ENSG00000117410<br>ENSG00000182158<br>ENSG00000105810<br>ENSG00000162434<br>ENSG00000110092<br>ENSG00000188064<br>ENSG00000082781<br>ENSG00000112576<br>ENSG00000213949<br>ENSG00000073756<br>ENSG00000155760<br>ENSG00000142166<br>ENSG00000115414<br>ENSG00000179111<br>ENSG00000130702<br>ENSG00000148400<br>ENSG00000005339<br>ENSG00000106003<br>ENSG00000132155<br>ENSG00000131323<br>ENSG00000164362<br>ENSG00000188153<br>ENSG00000080815	ITGB8 LAMB<br>THBS1 GSK3<br>WNT5A VT<br>OASL DVL<br>COL1A1<br>NFKB1 LAMI<br>ATP6V0B<br>CREB3L2<br>CDK6 JAK<br>CCND1<br>WNT7B ITG<br>CCND3 ITGA<br>PTGS2 FZD<br>IFNAR1 FN<br>HES7 LAMA<br>NOTCH1<br>CREBBP LFN<br>RAF1 TRAF<br>TERT COL4A<br>PSEN1 CDK<br>HRAS FADD<br>BAK1 HES4<br>SOS2 COL9A<br>TCF7L2 CHI<br>FZD2 DLG<br>MAPK3 AXIN<br>WNT10B<br>MAGI1 PAT<br>HDAC7<br>VEGFA ISG<br>STAT1 WNT<br>FZD5 ATM<br>TCIRG1<br>ITGA2

						ENSG00000135446<br>ENSG00000174775<br>ENSG00000168040<br>ENSG00000030110<br>ENSG00000188290<br>ENSG00000100485<br>ENSG00000092758<br>ENSG00000148737<br>ENSG00000111642<br>ENSG00000180340<br>ENSG00000075711<br>ENSG00000102882<br>ENSG00000103126<br>ENSG00000169884<br>ENSG00000151276<br>ENSG00000132849<br>ENSG00000061273<br>ENSG00000112715<br>ENSG00000187608<br>ENSG00000115415<br>ENSG00000115596<br>ENSG00000163251<br>ENSG00000149311<br>ENSG00000110719<br>ENSG00000164171<br>ENSG00000116039<br>ENSG00000197565<br>ENSG00000101680<br>ENSG00000171862<br>ENSG00000121879<br>ENSG00000147099<br>ENSG00000157240<br>ENSG00000114573<br>ENSG00000135862<br>ENSG00000142156<br>ENSG00000263528<br>ENSG00000185825<br>ENSG00000103479<br>ENSG00000184384<br>ENSG00000067606<br>ENSG00000005884<br>ENSG00000164683<br>ENSG00000187840<br>ENSG00000050555<br>ENSG00000064012<br>ENSG00000141510<br>ENSG00000169733	ATP6V1B1<br>COL4A6<br>LAMA1 PTE<br>PIK3CA<br>HDAC8 FZD<br>ATP6V1A<br>LAMC1<br>COL6A1<br>IKBKE<br>BCAP31 RBI<br>MAML2<br>PRKCZ ITG<br>HEY1<br>EIF4EBP1<br>LAMC3 CASI<br>TP53 RFN
hsa04621	NOD-like receptor signaling pathway	41/1188	133/5573	0.00601627891467234	0.153916468900367	ENSG00000136244<br>ENSG00000118503<br>ENSG00000150995<br>ENSG00000023445<br>ENSG00000169429<br>ENSG00000162645<br>ENSG00000080824<br>ENSG00000109320<br>ENSG00000162434<br>ENSG00000149782<br>ENSG00000177606<br>ENSG00000110330<br>ENSG00000142166<br>ENSG00000164733<br>ENSG00000112851<br>ENSG00000100906<br>ENSG00000139112<br>ENSG00000131323<br>ENSG00000163739<br>ENSG00000087077<br>ENSG00000101966<br>ENSG00000110218<br>ENSG00000168040<br>ENSG00000096384<br>ENSG00000157625<br>ENSG00000142185<br>ENSG00000185386<br>ENSG00000169604<br>ENSG00000096433<br>ENSG00000102882<br>ENSG00000188130<br>ENSG00000136560<br>ENSG00000115415<br>ENSG00000111335<br>ENSG00000116688	IL6 TNFAIP<br>ITPR1 BIRC<br>CXCL8 GBP<br>HSP90AA1<br>NFKB1 JAK<br>PLCB3 JUN<br>BIRC2 IFNA<br>CTSB ERBI<br>NFKBIA<br>GABARAPL<br>TRAF3 CXC<br>TRIP6 XIA<br>PANX1 FAD<br>HSP90AB1<br>TAB3 TRPM<br>MAPK11<br>ANTXR1<br>ITPR3 MAP<br>MAPK12<br>TANK STAT<br>OAS2 MFN<br>NOD1 NFKB<br>IKBKE TAB<br>TMEM173<br>CASP8

						ENSG00000106100 ENSG00000104825 ENSG00000263528 ENSG00000100324 ENSG00000184584 ENSG00000064012	
hsa05225	Hepatocellular carcinoma	44/1188	147/5573	0.00812242509374697	0.186267049645831	ENSG00000082701 ENSG00000114251 ENSG00000116717 ENSG00000166949 ENSG00000107404 ENSG00000105976 ENSG00000105810 ENSG00000110092 ENSG00000188064 ENSG00000184009 ENSG00000099860 ENSG00000134574 ENSG00000175387 ENSG00000155760 ENSG00000143198 ENSG00000132155 ENSG00000164362 ENSG00000135446 ENSG00000174775 ENSG00000030110 ENSG00000100485 ENSG00000147889 ENSG00000148737 ENSG00000085871 ENSG00000180340 ENSG00000189079 ENSG00000162337 ENSG00000102882 ENSG00000103126 ENSG00000116044 ENSG00000169884 ENSG00000115596 ENSG00000197943 ENSG00000163251 ENSG00000134184 ENSG00000163235 ENSG00000171862 ENSG00000121879 ENSG00000157240 ENSG00000108604 ENSG00000112242 ENSG00000157764 ENSG00000198431 ENSG00000141510	GSK3B WNT5A GADD45A SMAD3 DVL MET CDK6 CCND1 WNT7B ACTG1 GADD45B DDB2 SMAD FZD7 MGST RAF1 TERT CDK4 HRA BAK1 SOS2 CDKN2A TCF7L2 MGST2 FZD ARID2 LRP MAPK3 AXIN NFE2L2 WNT10B WNT6 PLCG FZD5 GSTM TGFA PTEN PIK3CA FZD SMARCD2 E2F3 BRAF TXNRD1 TP
hsa04933	AGE-RAGE signaling pathway in diabetic complications	29/1188	89/5573	0.0084942628502985	0.186267049645831	ENSG00000136244 ENSG00000169429 ENSG00000166949 ENSG00000108821 ENSG00000109320 ENSG00000168542 ENSG00000110092 ENSG00000149782 ENSG00000177606 ENSG00000137193 ENSG00000175387 ENSG00000090339 ENSG00000115414 ENSG00000171132 ENSG00000188153 ENSG00000135446 ENSG00000174775 ENSG00000185386 ENSG00000102882 ENSG00000188130 ENSG00000173511 ENSG00000112715 ENSG00000115415 ENSG00000197943 ENSG00000197565 ENSG00000121879 ENSG00000126561 ENSG00000007952 ENSG00000067606	IL6 CXCL8 SMAD3 COL1A1 NFKB1 COL3A1 CCND1 PLC JUN PIM1 SMAD2 ICAM FN1 PRKC COL4A5 CD HRAS MAPK MAPK3 MAPK12 VEGFB VEG STAT1 PLCG COL4A6 PIK3CA STAT5A NOX PRKCZ
hsa04141	Protein processing in endoplasmic reticulum	44/1188	149/5573	0.0104895329620317	0.20626522579005	ENSG00000162909 ENSG00000128272	CAPN2 ATF HSP90AA1

						ENSG00000080824<br>ENSG00000135924<br>ENSG00000150961<br>ENSG00000166598<br>ENSG00000140943<br>ENSG00000116661<br>ENSG00000133265<br>ENSG00000112208<br>ENSG00000132002<br>ENSG00000179218<br>ENSG00000102595<br>ENSG00000167004<br>ENSG00000138073<br>ENSG00000120694<br>ENSG00000173110<br>ENSG00000103266<br>ENSG00000160087<br>ENSG00000030110<br>ENSG00000180667<br>ENSG00000102580<br>ENSG00000136731<br>ENSG00000096384<br>ENSG00000155660<br>ENSG00000198162<br>ENSG00000072849<br>ENSG00000113615<br>ENSG00000175197<br>ENSG00000132432<br>ENSG00000179262<br>ENSG00000116044<br>ENSG00000115275<br>ENSG00000185624<br>ENSG00000118705<br>ENSG00000109846<br>ENSG00000087074<br>ENSG00000160803<br>ENSG00000067167<br>ENSG00000180879<br>ENSG00000118217<br>ENSG00000131871<br>ENSG00000100387<br>ENSG00000185825	DNAJB2<br>SEC24D<br>HSP90B1<br>MBTPS1<br>FBXO2<br>HSPBP1 BA<br>DNAJB1 CA<br>UGGT2 PDI<br>PREB HSP<br>HSPA6 STU<br>UBE2J2 BA<br>YOD1 DNAJ<br>UGGT1<br>HSP90AB1<br>PDIA4<br>MAN1A2<br>DERL2<br>SEC24A<br>DDIT3<br>SEC61G<br>RAD23A<br>NFE2L2 MO<br>P4HB RPN<br>CRYAB<br>PPP1R15A<br>UBQLN4<br>TRAM1 SSR<br>ATF6<br>SELENOS<br>RBX1 BCAP
[hsa03010]()	Ribosome	38/1188	126/5573	0.0116999295191911	0.20626522579005	ENSG00000143436<br>ENSG00000185088<br>ENSG00000128626<br>ENSG00000112651<br>ENSG00000109475<br>ENSG00000177954<br>ENSG00000163319<br>ENSG00000122140<br>ENSG00000186468<br>ENSG00000137818<br>ENSG00000105364<br>ENSG00000243147<br>ENSG00000063177<br>ENSG00000112306<br>ENSG00000137876<br>ENSG00000130312<br>ENSG00000168028<br>ENSG00000167526<br>ENSG00000114391<br>ENSG00000221983<br>ENSG00000108107<br>ENSG00000125445<br>ENSG00000159111<br>ENSG00000008988<br>ENSG00000180992<br>ENSG00000105640<br>ENSG00000116898<br>ENSG00000198242<br>ENSG00000198755<br>ENSG00000106591<br>ENSG00000116251<br>ENSG00000213741<br>ENSG00000147403<br>ENSG00000156482<br>ENSG00000161016<br>ENSG00000197756<br>ENSG00000188846<br>ENSG00000262814	MRPL9<br>RPS27L<br>MRPS12<br>MRPL2 RPL<br>RPS27<br>MRPS18C<br>MRPS2 RPS<br>RPLP1 MRP<br>MRPL33<br>RPL18 RPS<br>RSL24D1<br>MRPL34 RP<br>RPL13 RPL<br>UBA52 RPL<br>MRPS7<br>MRPL10<br>RPS20<br>MRPL14<br>RPL18A<br>MRPS15<br>RPL23A<br>RPL10A<br>MRPL32<br>RPL22 RPS<br>RPL10 RPL<br>RPL8 RPL3<br>RPL14<br>MRPL12
[hsa04620]()	Toll-like receptor signaling pathway	24/1188	72/5573	0.0117631092866874	0.20626522579005	ENSG00000136244<br>ENSG00000108984	IL6 MAP2K<br>CXCL8 NFK

						ENSG00000169429<br>ENSG00000109320<br>ENSG00000177606<br>ENSG00000142166<br>ENSG00000168811<br>ENSG00000100906<br>ENSG00000184216<br>ENSG00000131323<br>ENSG00000168040<br>ENSG00000185386<br>ENSG00000102882<br>ENSG00000188130<br>ENSG00000170345<br>ENSG00000115415<br>ENSG00000187554<br>ENSG00000121879<br>ENSG00000065559<br>ENSG00000263528<br>ENSG00000100324<br>ENSG00000143387<br>ENSG00000169248<br>ENSG00000064012	JUN IFNAR<br>IL12A NFKB<br>IRAK1 TRAF<br>FADD MAPK<br>MAPK3<br>MAPK12 FC<br>STAT1 TLR<br>PIK3CA<br>MAP2K4<br>IKBKE TAB<br>CTSK CXCL<br>CASP8
hsa05223	Non-small cell lung cancer	21/1188	61/5573	0.0120937265935534	0.20626522579005	ENSG00000116717<br>ENSG00000105810<br>ENSG00000068028<br>ENSG00000110092<br>ENSG00000099860<br>ENSG00000134574<br>ENSG00000132155<br>ENSG00000135446<br>ENSG00000174775<br>ENSG00000030110<br>ENSG00000100485<br>ENSG00000147889<br>ENSG00000101109<br>ENSG00000102882<br>ENSG00000197943<br>ENSG00000163235<br>ENSG00000121879<br>ENSG00000112242<br>ENSG00000126561<br>ENSG00000157764<br>ENSG00000141510	GADD45A<br>CDK6 RASSI<br>CCND1<br>GADD45B<br>DDB2 RAF<br>CDK4 HRA<br>BAK1 SOS<br>CDKN2A STI<br>MAPK3 PLC<br>TGFA PIK3<br>E2F3 STAT<br>BRAF TP5
hsa04934	Cushing syndrome	38/1188	128/5573	0.0152212732694476	0.226735260095891	ENSG00000130164<br>ENSG00000150995<br>ENSG00000082701<br>ENSG00000128272<br>ENSG00000114251<br>ENSG00000107404<br>ENSG00000133895<br>ENSG00000182158<br>ENSG00000105810<br>ENSG00000110092<br>ENSG00000149782<br>ENSG00000188064<br>ENSG00000185630<br>ENSG00000171303<br>ENSG00000155760<br>ENSG00000276045<br>ENSG00000135446<br>ENSG00000174233<br>ENSG00000147889<br>ENSG00000148737<br>ENSG00000073417<br>ENSG00000180340<br>ENSG00000144959<br>ENSG00000096433<br>ENSG00000102882<br>ENSG00000103126<br>ENSG00000162104<br>ENSG00000196363<br>ENSG00000169884<br>ENSG00000115596<br>ENSG00000163251<br>ENSG00000140459<br>ENSG00000157240<br>ENSG00000121281<br>ENSG00000112242<br>ENSG00000157764<br>ENSG00000196981<br>ENSG00000123358	LDLR ITPR<br>GSK3B ATF<br>WNT5A DVI<br>MEN1<br>CREB3L2<br>CDK6 CCND<br>PLCB3<br>WNT7B PB<br>KCNK3 FZD<br>ORAI1 CDK<br>ADCY6<br>CDKN2A<br>TCF7L2<br>PDE8A FZD<br>NCEH1 ITP<br>MAPK3 AXI<br>ADCY9 WD<br>WNT10B<br>WNT6 FZD<br>CYP11A1<br>FZD1 ADCY<br>E2F3 BRA<br>WDR5B<br>NR4A1

hsa05134	Legionellosis	16/1188	44/5573	0.0155429428558278	0.226735260095891	ENSG00000125730<br>ENSG00000136244<br>ENSG00000176171<br>ENSG00000077150<br>ENSG00000169429<br>ENSG00000109320<br>ENSG00000144381<br>ENSG00000168811<br>ENSG00000100906<br>ENSG00000163739<br>ENSG00000173110<br>ENSG00000187554<br>ENSG00000013441<br>ENSG00000143761<br>ENSG00000185122<br>ENSG00000064012	C3 IL6 BNIF<br>NFKB2 CXCI<br>NFKB1 HSPI<br>IL12A NFKB<br>CXCL1 HSPA<br>TLR5 CLK1<br>ARF1 HSF1<br>CASP8
hsa00533	Glycosaminoglycan biosynthesis - keratan sulfate	6/1188	11/5573	0.0158848063957023	0.226735260095891	ENSG00000086062<br>ENSG00000008513<br>ENSG00000175040<br>ENSG00000117411<br>ENSG00000158850<br>ENSG00000157350	B4GALT1<br>ST3GAL1<br>CHST2<br>B4GALT2<br>B4GALT3<br>ST3GAL2
hsa05224	Breast cancer	37/1188	125/5573	0.0172702821335468	0.226735260095891	ENSG00000077150<br>ENSG00000082701<br>ENSG00000114251<br>ENSG00000116717<br>ENSG00000184916<br>ENSG00000107404<br>ENSG00000105810<br>ENSG00000110092<br>ENSG00000188064<br>ENSG00000177606<br>ENSG00000099860<br>ENSG00000134574<br>ENSG00000155760<br>ENSG00000128917<br>ENSG00000148400<br>ENSG00000132155<br>ENSG00000135446<br>ENSG00000174775<br>ENSG00000030110<br>ENSG00000100485<br>ENSG00000148737<br>ENSG00000180340<br>ENSG00000162337<br>ENSG00000102882<br>ENSG00000103126<br>ENSG00000169884<br>ENSG00000170345<br>ENSG00000115596<br>ENSG00000163251<br>ENSG00000124151<br>ENSG00000171862<br>ENSG00000121879<br>ENSG00000157240<br>ENSG00000112242<br>ENSG00000157764<br>ENSG00000164683<br>ENSG00000141510	NFKB2 GSK3<br>WNT5A<br>GADD45A<br>JAG2 DVL1<br>CDK6 CCND<br>WNT7B JU<br>GADD45B<br>DDB2 FZD7<br>DLL4 NOTCI<br>RAF1 CDK4<br>HRAS BAK<br>SOS2 TCF7I<br>FZD2 LRP5<br>MAPK3 AXIN<br>WNT10B FC<br>WNT6 FZD<br>NCOA3 PTE<br>PIK3CA FZD<br>E2F3 BRAF<br>HEY1 TP53
hsa05145	Toxoplasmosis	25/1188	78/5573	0.0172760507155868	0.226735260095891	ENSG00000130164<br>ENSG00000108984<br>ENSG00000023445<br>ENSG00000196878<br>ENSG00000109320<br>ENSG00000172037<br>ENSG00000162434<br>ENSG00000110330<br>ENSG00000168811<br>ENSG00000027697<br>ENSG00000130702<br>ENSG00000100906<br>ENSG00000184216<br>ENSG00000101966<br>ENSG00000173110<br>ENSG00000185386<br>ENSG00000102882<br>ENSG00000188130<br>ENSG00000115415<br>ENSG00000101680<br>ENSG00000104825<br>ENSG00000135862	LDLR MAP2I<br>BIRC3 LAME<br>NFKB1 LAMI<br>JAK1 BIRC<br>IL12A IFNGI<br>LAMA5<br>NFKBIA IRAI<br>XIAP HSPA<br>MAPK11<br>MAPK3<br>MAPK12<br>STAT1 LAMA<br>NFKBIB<br>LAMC1 TAE<br>LAMC3 CASI

						ENSG00000100324<br>ENSG00000050555<br>ENSG00000064012	
hsa04512	ECM-receptor interaction	22/1188	67/5573	0.0185371482241989	0.226735260095891	ENSG00000124145<br>ENSG00000105855<br>ENSG00000196878<br>ENSG00000137801<br>ENSG00000109072<br>ENSG00000142798<br>ENSG00000108821<br>ENSG00000172037<br>ENSG00000082781<br>ENSG00000213949<br>ENSG00000115414<br>ENSG00000130702<br>ENSG00000188153<br>ENSG00000092758<br>ENSG00000173402<br>ENSG00000164171<br>ENSG00000197565<br>ENSG00000101680<br>ENSG00000135862<br>ENSG00000142156<br>ENSG00000005884<br>ENSG00000050555	SDC4 ITGB<br>LAMB3 THB<br>VTN HSPG<br>COL1A1<br>LAMB2 ITGI<br>ITGA1 FN<br>LAMA5<br>COL4A5<br>COL9A3 DA<br>ITGA2<br>COL4A6<br>LAMA1<br>LAMC1<br>COL6A1<br>ITGA3 LAM
hsa00790	Folate biosynthesis	10/1188	24/5573	0.0193497377977696	0.226735260095891	ENSG00000163283<br>ENSG00000085662<br>ENSG00000196139<br>ENSG00000228716<br>ENSG00000163286<br>ENSG00000131979<br>ENSG00000116096<br>ENSG00000159228<br>ENSG00000163295<br>ENSG00000180176	ALPP AKR1I<br>AKR1C3 DH<br>ALPG GCH<br>SPR CBR1<br>ALPI TH
hsa04510	Focal adhesion	49/1188	175/5573	0.0201639515927901	0.226735260095891	ENSG00000162909<br>ENSG00000105855<br>ENSG00000023445<br>ENSG00000196878<br>ENSG00000137801<br>ENSG00000082701<br>ENSG00000134215<br>ENSG00000109072<br>ENSG00000108821<br>ENSG00000172037<br>ENSG00000134318<br>ENSG00000105976<br>ENSG00000110092<br>ENSG00000082781<br>ENSG00000112576<br>ENSG00000177606<br>ENSG00000184009<br>ENSG00000213949<br>ENSG00000197702<br>ENSG00000110330<br>ENSG00000115414<br>ENSG00000130702<br>ENSG00000132155<br>ENSG00000101966<br>ENSG00000188153<br>ENSG00000058272<br>ENSG00000174775<br>ENSG00000100485<br>ENSG00000092758<br>ENSG00000050820<br>ENSG00000197122<br>ENSG00000140795<br>ENSG00000102882<br>ENSG00000173511<br>ENSG00000196924<br>ENSG00000035403<br>ENSG00000112715<br>ENSG00000164171<br>ENSG00000197565<br>ENSG00000101680<br>ENSG00000171862<br>ENSG00000121879<br>ENSG00000135862<br>ENSG00000142156<br>ENSG00000101608<br>ENSG00000157764	CAPN2 ITGI<br>BIRC3 LAMI<br>THBS1 GSK<br>VAV3 VTN<br>COL1A1<br>LAMB2<br>ROCK2 ME<br>CCND1 ITGI<br>CCND3 JUI<br>ACTG1 ITG<br>PARVA BIR<br>FN1 LAMA<br>RAF1 XIAF<br>COL4A5<br>PPP1R12A<br>HRAS SOS<br>COL9A3<br>BCAR1 SR<br>MYLK3<br>MAPK3<br>VEGFB FLN<br>VCL VEGF<br>ITGA2<br>COL4A6<br>LAMA1 PTE<br>PIK3CA<br>LAMC1<br>COL6A1<br>MYL12A BR<br>ITGA3 FLN<br>LAMC3

						ENSG00000005884 ENSG00000128591 ENSG00000050555	
hsa04658	Th1 and Th2 cell differentiation	21/1188	64/5573	0.0212883381225639	0.226735260095891	ENSG00000184916 ENSG00000109320 ENSG00000020633 ENSG00000162434 ENSG00000177606 ENSG00000146232 ENSG00000168811 ENSG00000027697 ENSG00000100906 ENSG00000128917 ENSG00000148400 ENSG00000185386 ENSG00000102882 ENSG00000188130 ENSG00000170345 ENSG00000115415 ENSG00000100385 ENSG00000104825 ENSG00000184384 ENSG00000126561 ENSG00000147168	JAG2 NFKB RUNX3 JAK JUN NFKBI IL12A IFNGI NFKBIA DLL NOTCH1 MAPK11 MAPK3 MAPK12 FC STAT1 IL2R NFKBIB MAML2 STAT5A IL2RG
hsa00515	Mannose type O-glycan biosynthesis	9/1188	21/5573	0.021412073626457	0.226735260095891	ENSG00000086062 ENSG00000117411 ENSG00000196371 ENSG00000158850 ENSG00000118600 ENSG00000130714 ENSG00000167889 ENSG00000085998 ENSG00000115526	B4GALT1 B4GALT2 FUT4 B4GALT3 RXYLT1 POMT1 MGAT5B POMGNT1 CHST10
hsa05162	Measles	28/1188	91/5573	0.0214179887386998	0.226735260095891	ENSG00000136244 ENSG00000118503 ENSG00000082701 ENSG00000109320 ENSG00000105810 ENSG00000162434 ENSG00000110092 ENSG00000112576 ENSG00000142166 ENSG00000168811 ENSG00000027697 ENSG00000100906 ENSG00000184216 ENSG00000173110 ENSG00000135446 ENSG00000115415 ENSG00000111335 ENSG00000100385 ENSG00000163743 ENSG00000147065 ENSG00000121879 ENSG00000104825 ENSG00000263528 ENSG00000126561 ENSG00000147677 ENSG00000147168 ENSG00000123595 ENSG00000141510	IL6 TNFAIP GSK3B NFKI CDK6 JAK CCND1 CCND3 IFNAR1 IL1 IFNGR1 NFKBIA IRA HSPA6 CDK STAT1 OAS IL2RB RCHY MSN PIK3C NFKBIB IKBI STAT5A EIF3H IL2R RAB9A TP5
hsa04927	Cortisol synthesis and secretion	16/1188	46/5573	0.0241248470705576	0.233312331422579	ENSG00000130164 ENSG00000150995 ENSG00000128272 ENSG00000182158 ENSG00000149782 ENSG00000185630 ENSG00000171303 ENSG00000276045 ENSG00000174233 ENSG00000073417 ENSG00000144959 ENSG00000096433 ENSG00000162104 ENSG00000140459 ENSG00000121281 ENSG00000123358	LDLR ITPR ATF4 CREB3L2 PLCB3 PBX KCNK3 ORA ADCY6 PDE NCEH1 ITPI ADCY9 CYP11A1 ADCY7 NR4
hsa05120	Epithelial cell signaling in Helicobacter pylori infection	20/1188	61/5573	0.0244652650609664	0.233312331422579	ENSG00000169429 ENSG00000109320 ENSG00000117410 ENSG00000105976	CXCL8 NFKI ATP6V0B MI JUN GIT1 C NFKBIA

						ENSG00000177606 ENSG00000108262 ENSG00000103653 ENSG00000100906 ENSG00000163739 ENSG00000137845 ENSG00000185386 ENSG00000197122 ENSG00000188130 ENSG00000197943 ENSG00000106100 ENSG00000110719 ENSG00000154721 ENSG00000116039 ENSG00000065559 ENSG00000114573	CXCL1 ADAM10 MAPK11 SR MAPK12 PLCG2 NOD TCIRG1 JAM ATP6V1B1 MAP2K4 ATP6V1A
[hsa04380]()	Osteoclast differentiation	28/1188	92/5573	0.0247247846946687	0.233312331422579	ENSG00000077150 ENSG00000108984 ENSG00000104856 ENSG00000164761 ENSG00000083799 ENSG00000109320 ENSG00000162434 ENSG00000177606 ENSG00000175592 ENSG00000142166 ENSG00000027697 ENSG00000100906 ENSG00000115641 ENSG00000184557 ENSG00000187098 ENSG00000185386 ENSG00000102882 ENSG00000188130 ENSG00000170345 ENSG00000115415 ENSG00000116701 ENSG00000197943 ENSG00000184371 ENSG00000121879 ENSG00000100324 ENSG00000007952 ENSG00000075426 ENSG00000143387	NFKB2 MAP2K6 REI TNFRSF11I CYLD NFKB JAK1 JUN FOSL1 IFNAR1 IFNGR1 NFKBIA FHI SOCS3 MIT MAPK11 MAPK3 MAPK12 FC STAT1 NCF PLCG2 CSF PIK3CA TAE NOX1 FOSL CTSK
[hsa04115]()	p53 signaling pathway	22/1188	69/5573	0.0260611519738397	0.233312331422579	ENSG00000137801 ENSG00000206075 ENSG00000130766 ENSG00000116717 ENSG00000172667 ENSG00000105810 ENSG00000146674 ENSG00000110092 ENSG00000112576 ENSG00000099860 ENSG00000134574 ENSG00000135446 ENSG00000147889 ENSG00000157456 ENSG00000141682 ENSG00000163743 ENSG00000149311 ENSG00000198625 ENSG00000171862 ENSG00000164054 ENSG00000064012 ENSG00000141510	THBS1 SERPINB5 SESN2 GADD45A ZMAT3 CDK IGFBP3 CCND1 CCND3 GADD45B DDB2 CDK CDKN2A CCNB2 PMAIP1 RCHY1 AT MDM4 PTE SHISA5 CAS TP53
[hsa05202]()	Transcriptional misregulation in cancer	43/1188	153/5573	0.0265859989695248	0.233312331422579	ENSG00000136244 ENSG00000023445 ENSG00000169429 ENSG00000172493 ENSG00000116717 ENSG00000132475 ENSG00000173801 ENSG00000152804 ENSG00000172216 ENSG00000109320 ENSG00000133895 ENSG00000105976 ENSG00000146674 ENSG00000147050 ENSG00000164120	IL6 BIRC3 CXCL8 AFF GADD45A H3F3B JUI HHEX CEBF NFKB1 MEN MET IGFBP KDM6A HPC JMJD1C GADD45B PBX1 DDB SIN3A RUN ETV4 SLC45A3 BAK1 TLX

						ENSG00000171988 ENSG00000099860 ENSG00000185630 ENSG00000134574 ENSG00000169375 ENSG00000159216 ENSG00000175832 ENSG00000158715 ENSG00000030110 ENSG00000164438 ENSG00000139083 ENSG00000071564 ENSG00000187098 ENSG00000125618 ENSG00000175197 ENSG00000168283 ENSG00000113916 ENSG00000253293 ENSG00000100385 ENSG00000149311 ENSG00000171843 ENSG00000115738 ENSG00000123268 ENSG00000104885 ENSG00000138756 ENSG00000136807 ENSG00000198728 ENSG00000141510	ETV6 TCF MITF PAX DDIT3 BMI BCL6 HOXA IL2RB ATM MLLT3 ID2 ATF1 DOT1 BMP2K CDK LDB1 TP5
hsa05230	Central carbon metabolism in cancer	18/1188	54/5573	0.0269978591944065	0.233312331422579	ENSG00000152256 ENSG00000105976 ENSG00000105281 ENSG00000138413 ENSG00000132155 ENSG00000174775 ENSG00000068078 ENSG00000102882 ENSG00000130489 ENSG00000100644 ENSG00000141526 ENSG00000156510 ENSG00000077463 ENSG00000117394 ENSG00000171862 ENSG00000121879 ENSG00000160211 ENSG00000141510	PDK1 MET SLC1A5 IDH RAF1 HRA FGFR3 MAP SCO2 HIF1 SLC16A3 HKDC1 SIRT SLC2A1 PTE PIK3CA G6P TP53
hsa04137	Mitophagy - animal	20/1188	62/5573	0.0291081190161847	0.233312331422579	ENSG00000176171 ENSG00000104765 ENSG00000123240 ENSG00000128272 ENSG00000177606 ENSG00000177169 ENSG00000139112 ENSG00000121749 ENSG00000174775 ENSG00000158828 ENSG00000187098 ENSG00000197122 ENSG00000100644 ENSG00000116688 ENSG00000136436 ENSG00000140983 ENSG00000188554 ENSG00000247077 ENSG00000164442 ENSG00000141510	BNIP3 BNIP OPTN ATF JUN ULK1 GABARAPL TBC1D15 HRAS PINK MITF SRC HIF1A MFN CALCOCO RHOT2 NBR PGAM5 CITED2 TP5
hsa05206	MicroRNAs in cancer	43/1188	154/5573	0.0295319246616885	0.233312331422579	ENSG00000168209 ENSG00000137801 ENSG00000206075 ENSG00000109320 ENSG00000105976 ENSG00000105810 ENSG00000068028 ENSG00000110092 ENSG00000150593 ENSG00000137193 ENSG00000073756 ENSG00000148400 ENSG00000005339 ENSG00000171132 ENSG00000132155	DDIT4 THB SERPINB5 NFKB1 ME CDK6 RASS CCND1 PDCD4 PIM PTGS2 NOTCH1 CREBBP PRKCE RAF SLC45A3 HRAS KIF2 BAK1 SOS FGFR3 SPR RPS6KA5

						ENSG00000158715<br>ENSG00000174775<br>ENSG00000137807<br>ENSG00000030110<br>ENSG00000100485<br>ENSG00000068078<br>ENSG00000136158<br>ENSG00000100784<br>ENSG00000147889<br>ENSG00000117632<br>ENSG00000139514<br>ENSG00000075618<br>ENSG00000168283<br>ENSG00000112715<br>ENSG00000124766<br>ENSG00000197943<br>ENSG00000169946<br>ENSG00000149311<br>ENSG00000198625<br>ENSG00000171862<br>ENSG00000121879<br>ENSG00000164045<br>ENSG00000112242<br>ENSG00000026025<br>ENSG00000146670<br>ENSG00000137710<br>ENSG00000106462<br>ENSG00000141510	CDKN2A<br>STMN1<br>SLC7A1<br>FSCN1 BMI<br>VEGFA SOX<br>PLCG2 ZFPM<br>ATM MDM<br>PTEN PIK3C<br>CDC25A E2I<br>VIM CDCA<br>RDX EZH2<br>TP53
hsa00514	Other types of O-glycan biosynthesis	9/1188	22/5573	0.0295523099338732	0.233312331422579	ENSG00000086062<br>ENSG00000130309<br>ENSG00000117411<br>ENSG00000158850<br>ENSG00000106003<br>ENSG00000106397<br>ENSG00000130714<br>ENSG00000163389<br>ENSG00000169733	B4GALT1<br>COLGALT<br>B4GALT2<br>B4GALT3<br>LFNG PLOD<br>POMT1<br>POGLUT1<br>RFNG
hsa04350	TGF-beta signaling pathway	23/1188	74/5573	0.0311589758577386	0.233312331422579	ENSG00000137801<br>ENSG00000164093<br>ENSG00000166949<br>ENSG00000139269<br>ENSG00000177426<br>ENSG00000175387<br>ENSG00000090539<br>ENSG00000005339<br>ENSG00000125845<br>ENSG00000117318<br>ENSG00000116985<br>ENSG00000102882<br>ENSG00000137834<br>ENSG00000095739<br>ENSG00000205250<br>ENSG00000115738<br>ENSG00000107779<br>ENSG00000118707<br>ENSG00000100387<br>ENSG00000133740<br>ENSG00000125968<br>ENSG00000198742<br>ENSG00000135503	THBS1 PITX<br>SMAD3 INHI<br>TGIF1 SMAI<br>CHRD<br>CREBBP BM<br>ID3 BMP8E<br>MAPK3<br>SMAD6 BAM<br>E2F4 ID2<br>BMPR1A<br>TGIF2 RBX<br>E2F5 ID1<br>SMURF1<br>ACVR1B
hsa04659	Th17 cell differentiation	23/1188	74/5573	0.0311589758577386	0.233312331422579	ENSG00000136244<br>ENSG00000080824<br>ENSG00000166949<br>ENSG00000109320<br>ENSG00000162434<br>ENSG00000177606<br>ENSG00000146232<br>ENSG00000175387<br>ENSG00000027697<br>ENSG00000100906<br>ENSG00000159216<br>ENSG00000104998<br>ENSG00000096384<br>ENSG00000185386<br>ENSG00000102882<br>ENSG00000188130<br>ENSG00000170345<br>ENSG00000100644<br>ENSG00000115415<br>ENSG00000100385<br>ENSG00000104825	IL6 HSP90A<br>SMAD3 NFK<br>JAK1 JUN<br>NFKBIE<br>SMAD2<br>IFNGR1<br>NFKBIA<br>RUNX1<br>IL27RA<br>HSP90AB1<br>MAPK11<br>MAPK3<br>MAPK12 FC<br>HIF1A STAT<br>IL2RB NFKB<br>STAT5A<br>IL2RG

						ENSG00000126561<br>ENSG00000147168	
hsa05146	Amoebiasis	23/1188	74/5573	0.0311589758577386	0.233312331422579	ENSG00000136244<br>ENSG00000196878<br>ENSG00000169429<br>ENSG00000108821<br>ENSG00000109320<br>ENSG00000172037<br>ENSG00000168542<br>ENSG00000149782<br>ENSG00000168811<br>ENSG00000115414<br>ENSG00000130702<br>ENSG00000081181<br>ENSG00000163739<br>ENSG00000188153<br>ENSG00000021355<br>ENSG00000111540<br>ENSG00000035403<br>ENSG00000106211<br>ENSG00000197565<br>ENSG00000101680<br>ENSG00000121879<br>ENSG00000135862<br>ENSG00000050555	IL6 LAMB3<br>CXCL8<br>COL1A1<br>NFKB1 LAM<br>COL3A1<br>PLCB3 IL12<br>FN1 LAMA<br>ARG2 CXCL<br>COL4A5<br>SERPINB1<br>RAB5B VC<br>HSPB1<br>COL4A6<br>LAMA1<br>PIK3CA<br>LAMC1<br>LAMC3
hsa05167	Kaposi sarcoma-associated herpesvirus infection	42/1188	151/5573	0.0331523208755073	0.242327678780494	ENSG00000125730<br>ENSG00000136244<br>ENSG00000150995<br>ENSG00000108984<br>ENSG00000169429<br>ENSG00000082701<br>ENSG00000109320<br>ENSG00000105810<br>ENSG00000162434<br>ENSG00000110092<br>ENSG00000177606<br>ENSG00000073756<br>ENSG00000142166<br>ENSG00000090339<br>ENSG00000027697<br>ENSG00000100906<br>ENSG00000005339<br>ENSG00000139112<br>ENSG00000132155<br>ENSG00000131323<br>ENSG00000163739<br>ENSG00000135446<br>ENSG00000174775<br>ENSG00000168040<br>ENSG00000030110<br>ENSG00000185386<br>ENSG00000197122<br>ENSG00000096433<br>ENSG00000172354<br>ENSG00000102882<br>ENSG00000188130<br>ENSG00000112715<br>ENSG00000170345<br>ENSG00000100644<br>ENSG00000115415<br>ENSG00000197943<br>ENSG00000121879<br>ENSG00000065559<br>ENSG00000263528<br>ENSG00000112242<br>ENSG00000064012<br>ENSG00000141510	C3 IL6 ITP<br>MAP2K6<br>CXCL8 GSK<br>NFKB1 CDK<br>JAK1 CCND<br>JUN PTGS<br>IFNAR1<br>ICAM1<br>IFNGR1<br>NFKBIA<br>CREBBP<br>GABARAPL<br>RAF1 TRAF<br>CXCL1 CDK<br>HRAS FAD<br>BAK1 MAPK<br>SRC ITPR<br>GNB2 MAPK<br>MAPK12<br>VEGFA FO<br>HIF1A STAT<br>PLCG2<br>PIK3CA<br>MAP2K4<br>IKBKE E2F<br>CASP8 TP5
hsa04218	Cellular senescence	39/1188	140/5573	0.0381797388624261	0.265035046583717	ENSG00000136244<br>ENSG00000162909<br>ENSG00000150995<br>ENSG00000108984<br>ENSG00000169429<br>ENSG00000116717<br>ENSG00000166949<br>ENSG00000109320<br>ENSG00000105810<br>ENSG00000146674<br>ENSG00000110092<br>ENSG00000112576<br>ENSG00000099860<br>ENSG00000175387	IL6 CAPN<br>ITPR1<br>MAP2K6<br>CXCL8<br>GADD45A<br>SMAD3 NFK<br>CDK6 IGFB<br>CCND1<br>CCND3<br>GADD45B<br>SMAD2 RAF<br>ZFP36L2<br>CDK4 HRA<br>CDKN2A

						ENSG00000132155 ENSG00000152518 ENSG00000135446 ENSG00000174775 ENSG00000147889 ENSG00000110422 ENSG00000185386 ENSG00000096433 ENSG00000157456 ENSG00000102882 ENSG00000188130 ENSG00000205250 ENSG00000149311 ENSG00000171497 ENSG00000064393 ENSG00000171862 ENSG00000121879 ENSG00000164045 ENSG00000112242 ENSG00000103479 ENSG00000111206 ENSG00000133740 ENSG00000187840 ENSG00000183814 ENSG00000141510	HIPK3 MAPK11 ITPR3 CCNI MAPK3 MAPK12 E2I ATM PPID HIPK2 PTE PIK3CA CDC25A E2I RBL2 FOXM E2F5 EIF4EBP1 LIN9 TP53
hsa05217	Basal cell carcinoma	18/1188	56/5573	0.0385442469217226	0.265035046583717	ENSG00000082701 ENSG00000114251 ENSG00000116717 ENSG00000107404 ENSG00000188064 ENSG00000099860 ENSG00000134574 ENSG00000155760 ENSG00000125845 ENSG00000030110 ENSG00000148737 ENSG00000180340 ENSG00000103126 ENSG00000169884 ENSG00000115596 ENSG00000163251 ENSG00000157240 ENSG00000141510	GSK3B WNT5A GADD45A DVL1 WNT7 GADD45B DDB2 FZD BMP2 BAK TCF7L2 FZI AXIN1 WNT10B WNT6 FZD FZD1 TP53
hsa04120	Ubiquitin mediated proteolysis	36/1188	128/5573	0.0395525440141355	0.265035046583717	ENSG00000023445 ENSG00000117399 ENSG00000049759 ENSG00000072422 ENSG00000116661 ENSG00000110330 ENSG00000134574 ENSG00000176386 ENSG00000099804 ENSG00000101966 ENSG00000103266 ENSG00000160087 ENSG00000114125 ENSG00000003096 ENSG00000184557 ENSG00000151148 ENSG00000198373 ENSG00000108395 ENSG00000033800 ENSG00000108106 ENSG00000163743 ENSG00000123124 ENSG00000008853 ENSG00000186591 ENSG00000141552 ENSG00000078747 ENSG00000100387 ENSG00000139842 ENSG00000102858 ENSG00000100023 ENSG00000044090 ENSG00000078043 ENSG00000160224 ENSG00000148634 ENSG00000198742 ENSG00000170142	BIRC3 CDC2 NEDD4L RHOBTB1 FBXO2 BIRC DDB2 CDC2 CDC34 XIA STUB1 UBE2J2 RNI KLHL13 SOCS3 UBE WWP2 TRIM37 PIAS UBE2S RCHY WWP1 RHOBTB2 UBE2H ANAPC11 ITCH RBX1 CUL4A MGRN1 PPII CUL7 PIAS AIRE HERC SMURF1 UBE2E1
hsa00190	Oxidative phosphorylation	35/1188	124/5573	0.0399737752032815	0.265035046583717	ENSG00000198804 ENSG00000117410	MT-CO1 ATP6V0B

						ENSG00000151366<br>ENSG00000099624<br>ENSG00000185633<br>ENSG00000198712<br>ENSG00000198786<br>ENSG00000179091<br>ENSG00000109390<br>ENSG00000212907<br>ENSG00000131174<br>ENSG00000198938<br>ENSG00000140990<br>ENSG00000198886<br>ENSG00000184076<br>ENSG00000115286<br>ENSG00000099795<br>ENSG00000127540<br>ENSG00000117118<br>ENSG00000164258<br>ENSG00000127184<br>ENSG00000110719<br>ENSG00000154723<br>ENSG00000116039<br>ENSG00000119421<br>ENSG00000105675<br>ENSG00000198840<br>ENSG00000183648<br>ENSG00000107902<br>ENSG00000114573<br>ENSG00000124172<br>ENSG00000198695<br>ENSG00000110717<br>ENSG00000233954<br>ENSG00000140740	NDUFC2<br>ATP5F1D<br>NDUFA4L<br>MT-CO2 MT<br>ND5 CYC1<br>NDUFC1 MT<br>ND4L COX7<br>MT-CO3<br>NDUFB10 M<br>ND4 UQCR1<br>NDUFS7<br>NDUFB7<br>UQCR11<br>SDHB NDUF<br>COX7C<br>TCIRG1<br>ATP5PF<br>ATP6V1B1<br>NDUFA8<br>ATP4A MT<br>ND3 NDUFE<br>LHPP<br>ATP6V1A<br>ATP5F1E M<br>ND6 NDUFS<br>UQCRHL<br>UQCRC2
[hsa05205]()	Proteoglycans in cancer	47/1188	174/5573	0.0410983227549801	0.265035046583717	ENSG00000124145<br>ENSG00000150995<br>ENSG00000137801<br>ENSG00000114251<br>ENSG00000109072<br>ENSG00000142798<br>ENSG00000134318<br>ENSG00000105976<br>ENSG00000140575<br>ENSG00000139329<br>ENSG00000110092<br>ENSG00000188064<br>ENSG00000082781<br>ENSG00000150593<br>ENSG00000184009<br>ENSG00000175387<br>ENSG00000155760<br>ENSG00000115414<br>ENSG00000132155<br>ENSG00000058272<br>ENSG00000174775<br>ENSG00000168101<br>ENSG00000100485<br>ENSG00000180340<br>ENSG00000145362<br>ENSG00000185386<br>ENSG00000197122<br>ENSG00000096433<br>ENSG00000102882<br>ENSG00000188130<br>ENSG00000196924<br>ENSG00000169884<br>ENSG00000112715<br>ENSG00000100644<br>ENSG00000115596<br>ENSG00000197943<br>ENSG00000163251<br>ENSG00000135047<br>ENSG00000164171<br>ENSG00000196914<br>ENSG00000147065<br>ENSG00000121879<br>ENSG00000157240<br>ENSG00000157764<br>ENSG00000128591<br>ENSG00000137710<br>ENSG00000141510	SDC4 ITPR<br>THBS1<br>WNT5A VT<br>HSPG2<br>ROCK2 ME<br>IQGAP1 LU<br>CCND1<br>WNT7B ITG<br>PDCD4<br>ACTG1<br>SMAD2 FZD<br>FN1 RAF1<br>PPP1R12A<br>HRAS<br>NUDT16L1<br>SOS2 FZD<br>ANK2 MAPK<br>SRC ITPR3<br>MAPK3<br>MAPK12 FLN<br>WNT10B<br>VEGFA HIF1<br>WNT6 PLCG<br>FZD5 CTSI<br>ITGA2<br>ARHGEF12<br>MSN PIK3C<br>FZD1 BRAI<br>FLNC RDX<br>TP53
[hsa05212]()	Pancreatic cancer	22/1188	72/5573	0.0414387043518515	0.265035046583717	ENSG00000116717<br>ENSG00000166949	GADD45A<br>SMAD3 NFK

						ENSG00000109320<br>ENSG00000105810<br>ENSG00000162434<br>ENSG00000110092<br>ENSG00000099860<br>ENSG00000134574<br>ENSG00000175387<br>ENSG00000132155<br>ENSG00000144118<br>ENSG00000135446<br>ENSG00000030110<br>ENSG00000147889<br>ENSG00000102882<br>ENSG00000112715<br>ENSG00000115415<br>ENSG00000163235<br>ENSG00000121879<br>ENSG00000112242<br>ENSG00000157764<br>ENSG00000141510	CDK6 JAK<br>CCND1<br>GADD45B<br>DDB2 SMA<br>RAF1 RAL<br>CDK4 BAK<br>CDKN2A<br>MAPK3<br>VEGFA STA<br>TGFA PIK3<br>E2F3 BRA<br>TP53
[hsa04625]()	C-type lectin receptor signaling pathway	24/1188	81/5573	0.0480740580904671	0.301198690485171	ENSG00000136244<br>ENSG00000077150<br>ENSG00000150995<br>ENSG00000069399<br>ENSG00000104856<br>ENSG00000083799<br>ENSG00000109320<br>ENSG00000177606<br>ENSG00000073756<br>ENSG00000168811<br>ENSG00000100906<br>ENSG00000132155<br>ENSG00000174775<br>ENSG00000185386<br>ENSG00000197122<br>ENSG00000096433<br>ENSG00000102882<br>ENSG00000188130<br>ENSG00000115415<br>ENSG00000197943<br>ENSG00000196914<br>ENSG00000121879<br>ENSG00000263528<br>ENSG00000064012	IL6 NFKB<br>ITPR1 BCL<br>RELB CYL<br>NFKB1 JU<br>PTGS2 IL1<br>NFKBIA RA<br>HRAS MAPK<br>SRC ITPR<br>MAPK3<br>MAPK12<br>STAT1 PLC<br>ARHGEF1<br>PIK3CA IKB<br>CASP8
[hsa00510]()	N-Glycan biosynthesis	15/1188	46/5573	0.0497360368337934	0.305340122368427	ENSG00000086062<br>ENSG00000117411<br>ENSG00000196547<br>ENSG00000158850<br>ENSG00000161013<br>ENSG00000198162<br>ENSG00000167889<br>ENSG00000179085<br>ENSG00000115275<br>ENSG00000101901<br>ENSG00000118705<br>ENSG00000172269<br>ENSG00000214160<br>ENSG00000175548<br>ENSG00000033011	B4GALT1<br>B4GALT2<br>MAN2A2<br>B4GALT3<br>MGAT4B<br>MAN1A2<br>MGAT5B<br>DPM3 MOG<br>ALG13 RP<br>DPAGT1<br>ALG3 ALG1<br>ALG1
[hsa00230]()	Purine metabolism	40/1188	147/5573	0.0507242548559928	0.305340122368427	ENSG00000113448<br>ENSG00000172572<br>ENSG00000112541<br>ENSG00000171408<br>ENSG00000113356<br>ENSG00000178921<br>ENSG00000115350<br>ENSG00000102978<br>ENSG00000178035<br>ENSG00000161980<br>ENSG00000135318<br>ENSG00000106348<br>ENSG00000148229<br>ENSG00000079739<br>ENSG00000243678<br>ENSG00000125630<br>ENSG00000181222<br>ENSG00000174233<br>ENSG00000116337<br>ENSG00000073417<br>ENSG00000285437<br>ENSG00000065989	PDE4D PDE<br>PDE10A<br>PDE7B<br>POLR3G PF<br>POLE4<br>POLR2C<br>IMPDH2<br>POLR3K NT<br>IMPDH1<br>POLE3 PG<br>NME2 POLR<br>POLR2A<br>ADCY6<br>AMPD2 PDE<br>POLR2J3<br>PDE4A NM<br>APRT ADC<br>AK3 POLR3<br>PDE9A NT5<br>POLD2<br>POLR2E<br>AMPD3

						ENSG00000103024<br>ENSG00000198931<br>ENSG00000162104<br>ENSG00000147853<br>ENSG00000058600<br>ENSG00000160191<br>ENSG00000076685<br>ENSG00000106628<br>ENSG00000099817<br>ENSG00000133805<br>ENSG00000121281<br>ENSG00000185100<br>ENSG00000115252<br>ENSG00000140057<br>ENSG00000062822<br>ENSG00000138185<br>ENSG00000159899<br>ENSG00000198805	ADCY7<br>ADSSL1<br>PDE1A AK<br>POLD1<br>ENTPD1 NP<br>PNP
[hsa05010]()	Alzheimer disease	42/1188	156/5573	0.0538128451774168	0.305572903683738	ENSG00000162909<br>ENSG00000123384<br>ENSG00000150995<br>ENSG00000082701<br>ENSG00000198804<br>ENSG00000176749<br>ENSG00000149782<br>ENSG00000111640<br>ENSG00000151366<br>ENSG00000099624<br>ENSG00000185633<br>ENSG00000198712<br>ENSG00000179091<br>ENSG00000080815<br>ENSG00000109390<br>ENSG00000168040<br>ENSG00000131174<br>ENSG00000198938<br>ENSG00000140990<br>ENSG00000142192<br>ENSG00000184076<br>ENSG00000137845<br>ENSG00000115286<br>ENSG00000099795<br>ENSG00000127540<br>ENSG00000096433<br>ENSG00000117118<br>ENSG00000102882<br>ENSG00000164258<br>ENSG00000127184<br>ENSG00000154723<br>ENSG00000162736<br>ENSG00000119421<br>ENSG00000183648<br>ENSG00000133318<br>ENSG00000118217<br>ENSG00000124172<br>ENSG00000198838<br>ENSG00000110717<br>ENSG00000233954<br>ENSG00000064012<br>ENSG00000140740	CAPN2 LRP<br>ITPR1 GSK<br>MT-CO1<br>CDK5R1<br>PLCB3<br>GAPDH<br>NDUFC2<br>ATP5F1D<br>NDUFA4L<br>MT-CO2 CY<br>PSEN1<br>NDUFC1<br>FADD COX7<br>MT-CO3<br>NDUFB10 A<br>UQCR10<br>ADAM10<br>NDUFS7<br>NDUFB7<br>UQCR11<br>ITPR3 SDH<br>MAPK3<br>NDUFS4<br>COX7C<br>ATP5PF<br>NCSTN<br>NDUFA8<br>NDUFB1<br>RTN3 ATF<br>ATP5F1E<br>RYR3 NDUF<br>UQCRHL<br>CASP8<br>UQCRC2
[hsa01522]()	Endocrine resistance	25/1188	86/5573	0.0545426335476288	0.305572903683738	ENSG00000184916<br>ENSG00000110092<br>ENSG00000177606<br>ENSG00000128917<br>ENSG00000148400<br>ENSG00000132155<br>ENSG00000164850<br>ENSG00000135446<br>ENSG00000174775<br>ENSG00000100485<br>ENSG00000174233<br>ENSG00000147889<br>ENSG00000142453<br>ENSG00000185386<br>ENSG00000197122<br>ENSG00000102882<br>ENSG00000188130<br>ENSG00000162104<br>ENSG00000170345<br>ENSG00000124151<br>ENSG00000121879<br>ENSG00000121281	JAG2 CCND<br>JUN DLL4<br>NOTCH1<br>RAF1 GPER<br>CDK4 HRA<br>SOS2 ADCY<br>CDKN2A<br>CARM1<br>MAPK11 SR<br>MAPK3<br>MAPK12<br>ADCY9 FO<br>NCOA3<br>PIK3CA<br>ADCY7 E2F<br>BRAF TP5

						ENSG00000112242<br>ENSG00000157764<br>ENSG00000141510	
hsa04064	NF-kappa B signaling pathway	21/1188	70/5573	0.0547155228096473	0.305572903683738	ENSG00000118503<br>ENSG00000077150<br>ENSG00000023445<br>ENSG00000169429<br>ENSG00000141527<br>ENSG00000104856<br>ENSG00000109320<br>ENSG00000099860<br>ENSG00000073756<br>ENSG00000110330<br>ENSG00000090339<br>ENSG00000100906<br>ENSG00000184216<br>ENSG00000131323<br>ENSG00000101966<br>ENSG00000157625<br>ENSG00000003402<br>ENSG00000197943<br>ENSG00000149311<br>ENSG00000082805<br>ENSG00000100324	TNFAIP3<br>NFKB2 BIR<br>CXCL8<br>CARD14 RE<br>NFKB1<br>GADD45B<br>PTGS2 BIR<br>ICAM1<br>NFKBIA IRA<br>TRAF3 XIA<br>TAB3 CFLA<br>PLCG2 AT<br>ERC1 TAB
hsa05210	Colorectal cancer	24/1188	82/5573	0.0547443312788456	0.305572903683738	ENSG00000082701<br>ENSG00000116717<br>ENSG00000166949<br>ENSG00000110092<br>ENSG00000177606<br>ENSG00000099860<br>ENSG00000134574<br>ENSG00000175387<br>ENSG00000132155<br>ENSG00000144118<br>ENSG00000174775<br>ENSG00000030110<br>ENSG00000100485<br>ENSG00000148737<br>ENSG00000124882<br>ENSG00000102882<br>ENSG00000141682<br>ENSG00000103126<br>ENSG00000170345<br>ENSG00000089685<br>ENSG00000163235<br>ENSG00000121879<br>ENSG00000157764<br>ENSG00000141510	GSK3B<br>GADD45A<br>SMAD3<br>CCND1 JU<br>GADD45B<br>DDB2 SMA<br>RAF1 RAL<br>HRAS BAK<br>SOS2 TCF7<br>EREG MAP<br>PMAIP1 AXI<br>FOS BIRC<br>TGFA PIK3<br>BRAF TP5
hsa05323	Rheumatoid arthritis	17/1188	55/5573	0.0613820874001706	0.336505371997364	ENSG00000136244<br>ENSG00000169429<br>ENSG00000115009<br>ENSG00000117410<br>ENSG00000177606<br>ENSG00000090339<br>ENSG00000095752<br>ENSG00000163739<br>ENSG00000112715<br>ENSG00000170345<br>ENSG00000110719<br>ENSG00000135047<br>ENSG00000116039<br>ENSG00000184371<br>ENSG00000164136<br>ENSG00000114573<br>ENSG00000143387	IL6 CXCL<br>CCL20<br>ATP6V0B J<br>ICAM1 IL1<br>CXCL1 VEG<br>FOS TCIRG<br>CTSL<br>ATP6V1B1<br>CSF1 IL15<br>ATP6V1A<br>CTSK
hsa05161	Hepatitis B	33/1188	121/5573	0.0690539952036162	0.371922395219477	ENSG00000136244<br>ENSG00000169429<br>ENSG00000128272<br>ENSG00000142798<br>ENSG00000166949<br>ENSG00000109320<br>ENSG00000182158<br>ENSG00000105810<br>ENSG00000162434<br>ENSG00000110092<br>ENSG00000177606<br>ENSG00000134574<br>ENSG00000142166<br>ENSG00000100906<br>ENSG00000005339<br>ENSG00000132155	IL6 CXCL<br>ATF4 HSPG<br>SMAD3 NFK<br>CREB3L2<br>CDK6 JAK<br>CCND1 JU<br>DDB2 IFNA<br>NFKBIA<br>CREBBP RA<br>CDK4 HRA<br>FADD SRC<br>MAPK3 FO<br>PTK2B STA<br>BIRC5 PTE<br>PIK3CA<br>MAP2K4

						ENSG00000135446 ENSG00000174775 ENSG00000168040 ENSG00000197122 ENSG00000102882 ENSG00000170345 ENSG00000120899 ENSG00000115415 ENSG00000089685 ENSG00000171862 ENSG00000121879 ENSG00000065559 ENSG00000263528 ENSG00000112242 ENSG00000126561 ENSG00000064012 ENSG00000141510	IKBKE E2F STAT5A CASP8 TP5
[hsa05142]()	Chagas disease (American trypanosomiasis)	22/1188	76/5573	0.0710743615961856	0.376203948448775	ENSG00000125730 ENSG00000136244 ENSG00000169429 ENSG00000166949 ENSG00000109320 ENSG00000149782 ENSG00000177606 ENSG00000175387 ENSG00000168811 ENSG00000027697 ENSG00000179218 ENSG00000100906 ENSG00000184216 ENSG00000168040 ENSG00000185386 ENSG00000102882 ENSG00000188130 ENSG00000003402 ENSG00000170345 ENSG00000121879 ENSG00000065559 ENSG00000064012	C3 IL6 CXC SMAD3 NFK PLCB3 JUN SMAD2 IL12 IFNGR1 CAL NFKBIA IRA FADD MAPK MAPK3 MAPK12 CFLAR FOS PIK3CA MAP2K4 CASP8
[hsa04926]()	Relaxin signaling pathway	30/1188	109/5573	0.0724547443934073	0.377010280148747	ENSG00000128272 ENSG00000166949 ENSG00000108821 ENSG00000109320 ENSG00000182158 ENSG00000168542 ENSG00000149782 ENSG00000177606 ENSG00000175387 ENSG00000100906 ENSG00000132155 ENSG00000188153 ENSG00000174775 ENSG00000100485 ENSG00000174233 ENSG00000185386 ENSG00000197122 ENSG00000172354 ENSG00000102882 ENSG00000188130 ENSG00000173511 ENSG00000162104 ENSG00000112715 ENSG00000170345 ENSG00000197565 ENSG00000121879 ENSG00000121281 ENSG00000065559 ENSG00000067606 ENSG00000137486	ATF4 SMAD COL1A1 NFKB1 CREB3L2 COL3A1 PLCB3 JUN SMAD2 NFKBIA RAF COL4A5 HRA SOS2 ADCY MAPK11 SR GNB2 MAPK MAPK12 VEGFB ADC VEGFA FO COL4A6 PIK3CA ADCY7 MAP2K4 PRKCZ ARR
[hsa05216]()	Thyroid cancer	11/1188	33/5573	0.0746822912827918	0.382124390396952	ENSG00000116717 ENSG00000110092 ENSG00000099860 ENSG00000134574 ENSG00000174775 ENSG00000030110 ENSG00000148737 ENSG00000125618 ENSG00000102882 ENSG00000157764 ENSG00000141510	GADD45A CCND1 GADD45B DDB2 HRA BAK1 TCF7 PAX8 MAPK BRAF TP5
[hsa00480]()	Glutathione metabolism	13/1188	41/5573	0.0793419238534166	0.391117769965337	ENSG00000128965	CHAC1 ODC

						ENSG00000115758<br>ENSG00000182054<br>ENSG00000116649<br>ENSG00000138413<br>ENSG00000143198<br>ENSG00000085871<br>ENSG00000197448<br>ENSG00000233276<br>ENSG00000100983<br>ENSG00000134184<br>ENSG00000160211<br>ENSG00000178814	IDH2 SRM<br>IDH1 MGST<br>MGST2 GST<br>GPX1 GSS<br>GSTM1 G6F<br>OPLAH
hsa05220	Chronic myeloid leukemia	21/1188	73/5573	0.0809959129923018	0.391117769965337	ENSG00000116717<br>ENSG00000166949<br>ENSG00000109320<br>ENSG00000105810<br>ENSG00000110092<br>ENSG00000099860<br>ENSG00000134574<br>ENSG00000100906<br>ENSG00000159216<br>ENSG00000132155<br>ENSG00000135446<br>ENSG00000174775<br>ENSG00000030110<br>ENSG00000100485<br>ENSG00000147889<br>ENSG00000102882<br>ENSG00000121879<br>ENSG00000112242<br>ENSG00000126561<br>ENSG00000157764<br>ENSG00000141510	GADD45A<br>SMAD3 NFK<br>CDK6 CCND<br>GADD45B<br>DDB2 NFKB<br>RUNX1 RAF<br>CDK4 HRA<br>BAK1 SOS2<br>CDKN2A<br>MAPK3<br>PIK3CA E2F<br>STAT5A BRA<br>TP53
hsa05214	Glioma	19/1188	65/5573	0.0820021366079631	0.391117769965337	ENSG00000116717<br>ENSG00000105810<br>ENSG00000110092<br>ENSG00000099860<br>ENSG00000134574<br>ENSG00000132155<br>ENSG00000135446<br>ENSG00000174775<br>ENSG00000030110<br>ENSG00000100485<br>ENSG00000147889<br>ENSG00000102882<br>ENSG00000197943<br>ENSG00000163235<br>ENSG00000171862<br>ENSG00000121879<br>ENSG00000112242<br>ENSG00000157764<br>ENSG00000141510	GADD45A<br>CDK6 CCND<br>GADD45B<br>DDB2 RAF<br>CDK4 HRA<br>BAK1 SOS2<br>CDKN2A<br>MAPK3 PLC<br>TGFA PTE<br>PIK3CA E2F<br>BRAF TP53
hsa05218	Melanoma	18/1188	61/5573	0.0822631261758009	0.391117769965337	ENSG00000116717<br>ENSG00000105976<br>ENSG00000105810<br>ENSG00000110092<br>ENSG00000099860<br>ENSG00000134574<br>ENSG00000132155<br>ENSG00000135446<br>ENSG00000174775<br>ENSG00000030110<br>ENSG00000147889<br>ENSG00000187098<br>ENSG00000102882<br>ENSG00000171862<br>ENSG00000121879<br>ENSG00000112242<br>ENSG00000157764<br>ENSG00000141510	GADD45A<br>MET CDK6<br>CCND1<br>GADD45B<br>DDB2 RAF<br>CDK4 HRA<br>BAK1 CDKN<br>MITF MAPK<br>PTEN PIK3C<br>E2F3 BRAF<br>TP53
hsa00410	beta-Alanine metabolism	9/1188	26/5573	0.0828099512955924	0.391117769965337	ENSG00000116649<br>ENSG00000006534<br>ENSG00000131471<br>ENSG00000133313<br>ENSG00000164904<br>ENSG00000108602<br>ENSG00000084754<br>ENSG00000117054<br>ENSG00000119711	SRM ALDH3<br>AOC3 CNDP<br>ALDH7A1<br>ALDH3A1<br>HADHA<br>ACADM<br>ALDH6A1
hsa05012	Parkinson disease	34/1188	128/5573	0.0897193078021153	0.417330719624991	ENSG00000198804<br>ENSG00000151366	MT-CO1<br>NDUFC2

						ENSG00000099624 ENSG00000185633 ENSG00000198712 ENSG00000170775 ENSG00000198786 ENSG00000179091 ENSG00000160087 ENSG00000115317 ENSG00000109390 ENSG00000212907 ENSG00000131174 ENSG00000158828 ENSG00000198938 ENSG00000140990 ENSG00000198886 ENSG00000184076 ENSG00000115286 ENSG00000099795 ENSG00000127540 ENSG00000117118 ENSG00000164258 ENSG00000127184 ENSG00000180176 ENSG00000154723 ENSG00000119421 ENSG00000198840 ENSG00000183648 ENSG00000124172 ENSG00000198695 ENSG00000110717 ENSG00000233954 ENSG00000140740	ATP5F1D NDUFA4L[illegible] MT-CO2 GPR37 MT[illegible] ND5 CYC1 UBE2J2 HTRA2 NDUFC1 M[illegible] ND4L COX[illegible] PINK1 MT-[illegible] CO3 NDUFB10 M[illegible] ND4 UQCR[illegible] NDUFS7 NDUFB7 UQCR11 SDHB NDUF[illegible] COX7C T[illegible] ATP5PF NDUFA8 M[illegible] ND3 NDUF[illegible] ATP5F1E M[illegible] ND6 NDUF[illegible] UQCRHL UQCRC2
[hsa04330]()	Notch signaling pathway	14/1188	46/5573	0.0942597485947848	0.420977362895548	ENSG00000184916 ENSG00000108773 ENSG00000107404 ENSG00000105245 ENSG00000128917 ENSG00000148400 ENSG00000005339 ENSG00000106003 ENSG00000080815 ENSG00000110042 ENSG00000162736 ENSG00000184384 ENSG00000138433 ENSG00000169733	JAG2 KAT2 DVL1 NUM[illegible] DLL4 NOTC[illegible] CREBBP LF[illegible] PSEN1 DTX NCSTN MAML2 CIR RFNG
[hsa04917]()	Prolactin signaling pathway	17/1188	58/5573	0.0943387635819127	0.420977362895548	ENSG00000082701 ENSG00000109320 ENSG00000110092 ENSG00000132155 ENSG00000174775 ENSG00000100485 ENSG00000184557 ENSG00000185386 ENSG00000197122 ENSG00000102882 ENSG00000188130 ENSG00000120833 ENSG00000170345 ENSG00000115415 ENSG00000180176 ENSG00000121879 ENSG00000126561	GSK3B NFK[illegible] CCND1 RA[illegible] HRAS SOS[illegible] SOCS3 MAPK11 SR[illegible] MAPK3 MAPK12 SOCS2 FO[illegible] STAT1 TH[illegible] PIK3CA STAT5A
[hsa05213]()	Endometrial cancer	16/1188	54/5573	0.0946170620188691	0.420977362895548	ENSG00000082701 ENSG00000116717 ENSG00000110092 ENSG00000099860 ENSG00000134574 ENSG00000132155 ENSG00000174775 ENSG00000030110 ENSG00000100485 ENSG00000148737 ENSG00000102882 ENSG00000103126 ENSG00000171862 ENSG00000121879 ENSG00000157764 ENSG00000141510	GSK3B GADD45A CCND1 GADD45B DDB2 RAF[illegible] HRAS BAK SOS2 TCF7[illegible] MAPK3 AXI[illegible] PTEN PIK3[illegible] BRAF TP5[illegible]
[hsa03013]()	RNA transport	40/1188	155/5573	0.101226294005626	0.443949603710387	ENSG00000100462 ENSG00000090621	PRMT5 PABPC4

						ENSG00000115211<br>ENSG00000100941<br>ENSG00000156976<br>ENSG00000100664<br>ENSG00000178718<br>ENSG00000254535<br>ENSG00000265241<br>ENSG00000132341<br>ENSG00000106263<br>ENSG00000051596<br>ENSG00000162231<br>ENSG00000070785<br>ENSG00000095319<br>ENSG00000124789<br>ENSG00000084623<br>ENSG00000114503<br>ENSG00000100401<br>ENSG00000006744<br>ENSG00000130811<br>ENSG00000173674<br>ENSG00000141543<br>ENSG00000184575<br>ENSG00000152464<br>ENSG00000129245<br>ENSG00000169371<br>ENSG00000172336<br>ENSG00000082516<br>ENSG00000023734<br>ENSG00000110321<br>ENSG00000005007<br>ENSG00000145191<br>ENSG00000151846<br>ENSG00000104131<br>ENSG00000147677<br>ENSG00000187840<br>ENSG00000015568<br>ENSG00000124571<br>ENSG00000092208	EIF2B4 PNI<br>EIF4A2 EIF<br>RPP25<br>PABPC4L<br>RBM8A RAI<br>EIF3B THOC<br>NXF1 EIF2E<br>NUP188<br>NUP153 EIF<br>NCBP2<br>RANGAP1<br>ELAC2 EIF3<br>EIF1AX<br>EIF4A3 XPC<br>RPP38 FXR<br>SNUPN POF<br>GEMIN5<br>STRAP<br>EIF4G2 UPF<br>EIF2B5<br>PABPC3 EIF<br>EIF3H<br>EIF4EBP1<br>RGPD5 XPC<br>GEMIN2
hsa05132	Salmonella infection	20/1188	71/5573	0.103925587345369	0.449368384718709	ENSG00000136244<br>ENSG00000169429<br>ENSG00000109320<br>ENSG00000134318<br>ENSG00000177606<br>ENSG00000184009<br>ENSG00000027697<br>ENSG00000163739<br>ENSG00000160447<br>ENSG00000133026<br>ENSG00000136950<br>ENSG00000185386<br>ENSG00000102882<br>ENSG00000188130<br>ENSG00000196924<br>ENSG00000170345<br>ENSG00000187554<br>ENSG00000070087<br>ENSG00000128591<br>ENSG00000065243	IL6 CXCL8<br>NFKB1 ROC<br>JUN ACTG<br>IFNGR1<br>CXCL1 PKN<br>MYH10<br>ARPC5L<br>MAPK11<br>MAPK3<br>MAPK12 FLN<br>FOS TLR5<br>PFN2 FLNC<br>PKN2
hsa01524	Platinum drug resistance	18/1188	63/5573	0.106391717032112	0.45159461459689	ENSG00000023445<br>ENSG00000110330<br>ENSG00000143198<br>ENSG00000101966<br>ENSG00000168040<br>ENSG00000030110<br>ENSG00000147889<br>ENSG00000085871<br>ENSG00000102882<br>ENSG00000141682<br>ENSG00000149311<br>ENSG00000134184<br>ENSG00000089685<br>ENSG00000121879<br>ENSG00000165240<br>ENSG00000009413<br>ENSG00000064012<br>ENSG00000141510	BIRC3 BIRC<br>MGST3 XIA<br>FADD BAK<br>CDKN2A<br>MGST2<br>MAPK3<br>PMAIP1 AT<br>GSTM1 BIRC<br>PIK3CA<br>ATP7A REV<br>CASP8 TP5
hsa05163	Human cytomegalovirus infection	44/1188	173/5573	0.107382432786883	0.45159461459689	ENSG00000136244<br>ENSG00000170776<br>ENSG00000150995<br>ENSG00000108984<br>ENSG00000169429	IL6 AKAP1<br>ITPR1<br>MAP2K6<br>CXCL8 GSK<br>ATF4 NFKB

						ENSG00000082701<br>ENSG00000128272<br>ENSG00000109320<br>ENSG00000134318<br>ENSG00000182158<br>ENSG00000105810<br>ENSG00000162434<br>ENSG00000110092<br>ENSG00000149782<br>ENSG00000073756<br>ENSG00000179218<br>ENSG00000100906<br>ENSG00000167004<br>ENSG00000132155<br>ENSG00000135446<br>ENSG00000174775<br>ENSG00000168040<br>ENSG00000030110<br>ENSG00000100485<br>ENSG00000174233<br>ENSG00000147889<br>ENSG00000050820<br>ENSG00000185386<br>ENSG00000197122<br>ENSG00000096433<br>ENSG00000172354<br>ENSG00000102882<br>ENSG00000188130<br>ENSG00000162104<br>ENSG00000112715<br>ENSG00000120899<br>ENSG00000196914<br>ENSG00000121879<br>ENSG00000121281<br>ENSG00000112242<br>ENSG00000187840<br>ENSG00000184584<br>ENSG00000064012<br>ENSG00000141510	ROCK2<br>CREB3L2<br>CDK6 JAK<br>CCND1 PLC<br>PTGS2 CAL<br>NFKBIA PDI<br>RAF1 CDK<br>HRAS FAD<br>BAK1 SOS<br>ADCY6<br>CDKN2A<br>BCAR1<br>MAPK11 SR<br>ITPR3 GNB<br>MAPK3<br>MAPK12<br>ADCY9 VEG<br>PTK2B<br>ARHGEF1<br>PIK3CA<br>ADCY7 E2F<br>EIF4EBP1<br>TMEM173<br>CASP8 TP5
[hsa04310]()	Wnt signaling pathway	31/1188	118/5573	0.113839148505683	0.468022278604662	ENSG00000082701<br>ENSG00000114251<br>ENSG00000107984<br>ENSG00000166949<br>ENSG00000107404<br>ENSG00000134318<br>ENSG00000087095<br>ENSG00000110092<br>ENSG00000149782<br>ENSG00000188064<br>ENSG00000175792<br>ENSG00000112576<br>ENSG00000177606<br>ENSG00000175592<br>ENSG00000145506<br>ENSG00000155760<br>ENSG00000005339<br>ENSG00000012211<br>ENSG00000080815<br>ENSG00000148737<br>ENSG00000180340<br>ENSG00000162337<br>ENSG00000103126<br>ENSG00000169884<br>ENSG00000095739<br>ENSG00000115596<br>ENSG00000163251<br>ENSG00000100592<br>ENSG00000157240<br>ENSG00000100387<br>ENSG00000141510	GSK3B<br>WNT5A DK<br>SMAD3 DVL<br>ROCK2 NL<br>CCND1 PLC<br>WNT7B<br>RUVBL1<br>CCND3 JU<br>FOSL1 NKD<br>FZD7 CREB<br>PRICKLE3<br>PSEN1<br>TCF7L2 FZD<br>LRP5 AXIN<br>WNT10B<br>BAMBI WNT<br>FZD5 DAAM<br>FZD1 RBX<br>TP53
[hsa04919]()	Thyroid hormone signaling pathway	26/1188	97/5573	0.115533956088888	0.468022278604662	ENSG00000211448<br>ENSG00000082701<br>ENSG00000143153<br>ENSG00000108773<br>ENSG00000110092<br>ENSG00000149782<br>ENSG00000184009<br>ENSG00000169375<br>ENSG00000148400<br>ENSG00000005339<br>ENSG00000132155<br>ENSG00000151090	DIO2 GSK3<br>ATP1B1<br>KAT2A<br>CCND1 PLC<br>ACTG1 SIN<br>NOTCH1<br>CREBBP RA<br>THRB HRA<br>MED17 SR<br>MAPK3<br>TBC1D4<br>HIF1A STA

						ENSG00000174775<br>ENSG00000042429<br>ENSG00000197122<br>ENSG00000102882<br>ENSG00000136111<br>ENSG00000100644<br>ENSG00000115415<br>ENSG00000197943<br>ENSG00000117394<br>ENSG00000124151<br>ENSG00000121879<br>ENSG00000008838<br>ENSG00000160563<br>ENSG00000141510	PLCG2<br>SLC2A1<br>NCOA3<br>PIK3CA<br>MED24 MED<br>TP53
hsa01230	Biosynthesis of amino acids	19/1188	68/5573	0.118218260718879	0.468022278604662	ENSG00000173599<br>ENSG00000135069<br>ENSG00000109107<br>ENSG00000102144<br>ENSG00000182054<br>ENSG00000146733<br>ENSG00000021826<br>ENSG00000111640<br>ENSG00000138413<br>ENSG00000166411<br>ENSG00000070669<br>ENSG00000081181<br>ENSG00000166123<br>ENSG00000108515<br>ENSG00000143811<br>ENSG00000153574<br>ENSG00000130707<br>ENSG00000116984<br>ENSG00000183010	PC PSAT1<br>ALDOC PG<br>IDH2 PSP<br>CPS1 GAPD<br>IDH1 IDH3<br>ASNS ARG<br>GPT2 ENO<br>PYCR2 RP<br>ASS1 MTR<br>PYCR1
hsa04390	Hippo signaling pathway	36/1188	140/5573	0.119823478489088	0.468022278604662	ENSG00000187079<br>ENSG00000150457<br>ENSG00000082701<br>ENSG00000114251<br>ENSG00000118523<br>ENSG00000166949<br>ENSG00000107404<br>ENSG00000068028<br>ENSG00000110092<br>ENSG00000188064<br>ENSG00000112576<br>ENSG00000184009<br>ENSG00000129474<br>ENSG00000110330<br>ENSG00000175387<br>ENSG00000155760<br>ENSG00000125845<br>ENSG00000116985<br>ENSG00000148737<br>ENSG00000180340<br>ENSG00000075711<br>ENSG00000128245<br>ENSG00000018408<br>ENSG00000103126<br>ENSG00000169884<br>ENSG00000132849<br>ENSG00000115596<br>ENSG00000163251<br>ENSG00000089685<br>ENSG00000134376<br>ENSG00000115738<br>ENSG00000107779<br>ENSG00000157240<br>ENSG00000067606<br>ENSG00000125968<br>ENSG00000139926	TEAD1 LAT<br>GSK3B<br>WNT5A CT<br>SMAD3 DVL<br>RASSF1<br>CCND1<br>WNT7B<br>CCND3<br>ACTG1<br>AJUBA BIR<br>SMAD2 FZD<br>BMP2 BMP<br>TCF7L2 FZD<br>DLG1 YWH<br>WWTR1<br>AXIN1<br>WNT10B<br>PATJ WNT<br>FZD5 BIRC<br>CRB1 ID2<br>BMPR1A FZ<br>PRKCZ ID<br>FRMD6
hsa04932	Non-alcoholic fatty liver disease (NAFLD)	35/1188	136/5573	0.122625355988637	0.468022278604662	ENSG00000136244<br>ENSG00000169429<br>ENSG00000082701<br>ENSG00000128272<br>ENSG00000198804<br>ENSG00000109320<br>ENSG00000177606<br>ENSG00000151366<br>ENSG00000105723<br>ENSG00000185633<br>ENSG00000198712<br>ENSG00000179091	IL6 CXCL<br>GSK3B ATF<br>MT-CO1<br>NFKB1 JU<br>NDUFC2<br>GSK3A<br>NDUFA4L<br>MT-CO2 CY<br>NDUFC1<br>COX7B SOC<br>SREBF1 MT<br>CO3

						ENSG00000109390 ENSG00000131174 ENSG00000184557 ENSG00000072310 ENSG00000198938 ENSG00000140990 ENSG00000184076 ENSG00000115286 ENSG00000171105 ENSG00000099795 ENSG00000127540 ENSG00000175197 ENSG00000117118 ENSG00000164258 ENSG00000127184 ENSG00000119421 ENSG00000183648 ENSG00000121879 ENSG00000078747 ENSG00000110717 ENSG00000233954 ENSG00000064012 ENSG00000140740	NDUFB10 UQCR10 NDUFS7 INS NDUFB7 UQCR11 DDIT3 SDH NDUFS4 COX7C NDUFA8 NDUFB1 PIK3CA ITC NDUFS8 UQCRHL CASP8 UQCRC2
hsa04068	FoxO signaling pathway	31/1188	119/5573	0.123946938528155	0.468022278604662	ENSG00000136244 ENSG00000176171 ENSG00000116717 ENSG00000166949 ENSG00000112096 ENSG00000087095 ENSG00000110092 ENSG00000099860 ENSG00000118515 ENSG00000175387 ENSG00000005339 ENSG00000139112 ENSG00000132155 ENSG00000126457 ENSG00000174775 ENSG00000100485 ENSG00000101109 ENSG00000166851 ENSG00000184481 ENSG00000185386 ENSG00000171105 ENSG00000157456 ENSG00000102882 ENSG00000188130 ENSG00000113916 ENSG00000149311 ENSG00000171862 ENSG00000121879 ENSG00000103479 ENSG00000121691 ENSG00000157764	IL6 BNIP3 GADD45A SMAD3 SOD NLK CCND GADD45B SGK1 SMAD CREBBP GABARAPL RAF1 PRMT HRAS SOS STK4 PLK FOXO4 MAPK11 INS CCNB2 MAPK3 MAPK12 BC ATM PTEN PIK3CA RBL CAT BRAF
hsa04110	Cell cycle	31/1188	119/5573	0.123946938528155	0.468022278604662	ENSG00000082701 ENSG00000116717 ENSG00000117399 ENSG00000166949 ENSG00000105810 ENSG00000110092 ENSG00000112576 ENSG00000099860 ENSG00000164754 ENSG00000175387 ENSG00000005339 ENSG00000127564 ENSG00000176386 ENSG00000135446 ENSG00000147889 ENSG00000166851 ENSG00000157456 ENSG00000128245 ENSG00000114126 ENSG00000205250 ENSG00000149311 ENSG00000141552 ENSG00000116670 ENSG00000134480 ENSG00000164109 ENSG00000100387 ENSG00000164045 ENSG00000112242	GSK3B GADD45A CDC20 SMA CDK6 CCND CCND3 GADD45B RAD21 SMA CREBBP PKMYT1 CDC26 CDK CDKN2A PLI CCNB2 YWHAH TFDP2 E2F ATM ANAPC11 MAD2L2 CCNH MAD2L1 RB CDC25A E2 RBL2 E2F TP53

						ENSG00000103479 ENSG00000133740 ENSG00000141510	
hsa04215	Apoptosis - multiple species	10/1188	32/5573	0.125009207966066	0.468022278604662	ENSG00000023445 ENSG00000110330 ENSG00000101966 ENSG00000115317 ENSG00000168040 ENSG00000030110 ENSG00000141682 ENSG00000176720 ENSG00000089685 ENSG00000064012	BIRC3 BIRC XIAP HTRA FADD BAK PMAIP1 BO BIRC5 CASF
hsa05321	Inflammatory bowel disease (IBD)	10/1188	32/5573	0.125009207966066	0.468022278604662	ENSG00000136244 ENSG00000166949 ENSG00000109320 ENSG00000177606 ENSG00000175387 ENSG00000168811 ENSG00000027697 ENSG00000115415 ENSG00000187554 ENSG00000147168	IL6 SMAD3 NFKB1 JUN SMAD2 IL12 IFNGR1 STAT1 TLR IL2RG
hsa00240	Pyrimidine metabolism	25/1188	94/5573	0.129616754222647	0.479425825859671	ENSG00000158825 ENSG00000113356 ENSG00000084774 ENSG00000115350 ENSG00000102978 ENSG00000161980 ENSG00000135318 ENSG00000183696 ENSG00000148229 ENSG00000102967 ENSG00000243678 ENSG00000125630 ENSG00000181222 ENSG00000285437 ENSG00000103024 ENSG00000168393 ENSG00000025708 ENSG00000058600 ENSG00000076685 ENSG00000106628 ENSG00000179958 ENSG00000099817 ENSG00000062822 ENSG00000138185 ENSG00000198805	CDA POLR3 CAD POLE POLR2C POLR3K NT UPP1 POLE DHODH NMI POLR1B POLR2A POLR2J3 NME3 DTYM TYMP POLR NT5C2 POLI DCTPP1 POLR2E POLD1 ENTPD1 PN
hsa04810	Regulation of actin cytoskeleton	44/1188	177/5573	0.141416612472752	0.516169871709596	ENSG00000105855 ENSG00000134215 ENSG00000134318 ENSG00000140575 ENSG00000082781 ENSG00000108262 ENSG00000184009 ENSG00000213949 ENSG00000148180 ENSG00000102302 ENSG00000115414 ENSG00000160867 ENSG00000132155 ENSG00000156886 ENSG00000058272 ENSG00000174775 ENSG00000133026 ENSG00000100485 ENSG00000068078 ENSG00000050820 ENSG00000136950 ENSG00000197122 ENSG00000140795 ENSG00000102882 ENSG00000084112 ENSG00000035403 ENSG00000136002 ENSG00000139734 ENSG00000172757 ENSG00000254999 ENSG00000102606 ENSG00000164171 ENSG00000006747	ITGB8 VAV ROCK2 IQGAP1 ITGB5 GIT ACTG1 ITGA GSN FGD1 FN1 FGFR RAF1 ITGA PPP1R12A HRAS MYH1 SOS2 FGFR BCAR1 ARPC5L SR MYLK3 MAPK3 SSH VCL ARHGE DIAPH3 CFL BRK1 ARHGEF7 ITGA2 SCII ARHGEF12 LIMK1 PIP5K1A MS PIK3CA MYL12A BRA PFN2 ITGA ITGA3 RDX

						ENSG00000196914<br>ENSG00000106683<br>ENSG00000143398<br>ENSG00000147065<br>ENSG00000121879<br>ENSG00000101608<br>ENSG00000157764<br>ENSG00000070087<br>ENSG00000083457<br>ENSG00000005884<br>ENSG00000137710	
[hsa04925](#)	Aldosterone synthesis and secretion	20/1188	74/5573	0.144015671638104	0.516169871709596	ENSG00000130164<br>ENSG00000150995<br>ENSG00000128272<br>ENSG00000143153<br>ENSG00000182158<br>ENSG00000149782<br>ENSG00000171303<br>ENSG00000105287<br>ENSG00000171132<br>ENSG00000276045<br>ENSG00000174233<br>ENSG00000058668<br>ENSG00000070961<br>ENSG00000096433<br>ENSG00000162104<br>ENSG00000140459<br>ENSG00000121281<br>ENSG00000123268<br>ENSG00000164535<br>ENSG00000123358	LDLR ITPR<br>ATF4 ATP1<br>CREB3L2<br>PLCB3 KCN<br>PRKD2 PRK<br>ORAI1 ADC<br>ATP2B4<br>ATP2B1<br>ITPR3 ADC<br>CYP11A1<br>ADCY7 ATF<br>DAGLB NR4
[hsa05226](#)	Gastric cancer	31/1188	121/5573	0.14577969326196	0.516169871709596	ENSG00000082701<br>ENSG00000114251<br>ENSG00000116717<br>ENSG00000173801<br>ENSG00000166949<br>ENSG00000107404<br>ENSG00000105976<br>ENSG00000110092<br>ENSG00000188064<br>ENSG00000099860<br>ENSG00000134574<br>ENSG00000175387<br>ENSG00000155760<br>ENSG00000132155<br>ENSG00000164362<br>ENSG00000174775<br>ENSG00000030110<br>ENSG00000100485<br>ENSG00000148737<br>ENSG00000180340<br>ENSG00000162337<br>ENSG00000102882<br>ENSG00000103126<br>ENSG00000169884<br>ENSG00000115596<br>ENSG00000163251<br>ENSG00000121879<br>ENSG00000157240<br>ENSG00000112242<br>ENSG00000157764<br>ENSG00000141510	GSK3B<br>WNT5A<br>GADD45A J<br>SMAD3 DVL<br>MET CCND<br>WNT7B<br>GADD45B<br>DDB2 SMAD<br>FZD7 RAF<br>TERT HRA<br>BAK1 SOS<br>TCF7L2 FZD<br>LRP5 MAPK<br>AXIN1<br>WNT10B<br>WNT6 FZD<br>PIK3CA FZD<br>E2F3 BRA<br>TP53
[hsa05416](#)	Viral myocarditis	9/1188	29/5573	0.146276152569169	0.516169871709596	ENSG00000110092<br>ENSG00000184009<br>ENSG00000090339<br>ENSG00000143322<br>ENSG00000154639<br>ENSG00000173402<br>ENSG00000198947<br>ENSG00000110321<br>ENSG00000064012	CCND1<br>ACTG1 ICA<br>ABL2 CXAD<br>DAG1 DMD<br>EIF4G2 CAS
[hsa04151](#)	PI3K-Akt signaling pathway	66/1188	275/5573	0.149638567938273	0.522034549511929	ENSG00000136244<br>ENSG00000105855<br>ENSG00000168209<br>ENSG00000196878<br>ENSG00000137801<br>ENSG00000082701<br>ENSG00000169242<br>ENSG00000128272<br>ENSG00000109072<br>ENSG00000080824	IL6 ITGB8<br>DDIT4 LAM<br>THBS1 GSK<br>EFNA1 ATF<br>VTN<br>HSP90AA1<br>COL1A1<br>NFKB1 LAM<br>CREB3L2 M<br>CDK6 JAK

						ENSG00000108821<br>ENSG00000109320<br>ENSG00000172037<br>ENSG00000182158<br>ENSG00000105976<br>ENSG00000105810<br>ENSG00000162434<br>ENSG00000110092<br>ENSG00000082781<br>ENSG00000112576<br>ENSG00000166598<br>ENSG00000118515<br>ENSG00000213949<br>ENSG00000142166<br>ENSG00000115414<br>ENSG00000130702<br>ENSG00000160867<br>ENSG00000132155<br>ENSG00000142627<br>ENSG00000188153<br>ENSG00000160447<br>ENSG00000135446<br>ENSG00000174775<br>ENSG00000100485<br>ENSG00000068078<br>ENSG00000096384<br>ENSG00000092758<br>ENSG00000124882<br>ENSG00000171105<br>ENSG00000172354<br>ENSG00000102882<br>ENSG00000128245<br>ENSG00000173511<br>ENSG00000151276<br>ENSG00000112715<br>ENSG00000100385<br>ENSG00000040199<br>ENSG00000213218<br>ENSG00000164171<br>ENSG00000184371<br>ENSG00000163235<br>ENSG00000197565<br>ENSG00000101680<br>ENSG00000171862<br>ENSG00000121879<br>ENSG00000135862<br>ENSG00000142156<br>ENSG00000103479<br>ENSG00000005884<br>ENSG00000105401<br>ENSG00000187840<br>ENSG00000065243<br>ENSG00000147168<br>ENSG00000050555<br>ENSG00000123358<br>ENSG00000141510	CCND1 ITGI<br>CCND3<br>HSP90B1<br>SGK1 ITGA<br>IFNAR1 FN<br>LAMA5 FGFI<br>RAF1 EPHA<br>COL4A5 PKI<br>CDK4 HRA<br>SOS2 FGFR<br>HSP90AB1<br>COL9A3 ERI<br>INSR GNB2<br>MAPK3<br>YWHAH<br>VEGFB MAG<br>VEGFA IL2F<br>PHLPP2 CSI<br>ITGA2 CSF<br>TGFA COL4<br>LAMA1 PTE<br>PIK3CA<br>LAMC1<br>COL6A1 RBI<br>ITGA3 CDC<br>EIF4EBP1<br>PKN2 IL2R<br>LAMC3 NR4<br>TP53
[hsa05131]()	Shigellosis	17/1188	62/5573	0.152933247161476	0.527047903000609	ENSG00000169429<br>ENSG00000109320<br>ENSG00000134318<br>ENSG00000275895<br>ENSG00000184009<br>ENSG00000100906<br>ENSG00000136950<br>ENSG00000185386<br>ENSG00000197122<br>ENSG00000102882<br>ENSG00000188130<br>ENSG00000035403<br>ENSG00000106100<br>ENSG00000116670<br>ENSG00000104825<br>ENSG00000102890<br>ENSG00000070087	CXCL8 NFKI<br>ROCK2<br>U2AF1L5<br>ACTG1<br>NFKBIA<br>ARPC5L<br>MAPK11 SR<br>MAPK3<br>MAPK12 VC<br>NOD1<br>MAD2L2<br>NFKBIB<br>ELMO3 PFN
[hsa04611]()	Platelet activation	25/1188	96/5573	0.155127582325496	0.527047903000609	ENSG00000150995<br>ENSG00000108821<br>ENSG00000095303<br>ENSG00000134318<br>ENSG00000168542<br>ENSG00000149782<br>ENSG00000184009<br>ENSG00000276045<br>ENSG00000058272	ITPR1<br>COL1A1<br>PTGS1 ROC<br>COL3A1<br>PLCB3 ACT<br>ORAI1<br>PPP1R12A<br>ADCY6<br>MAPK11 SR

						ENSG00000174233<br>ENSG00000185386<br>ENSG00000197122<br>ENSG00000140795<br>ENSG00000096433<br>ENSG00000102882<br>ENSG00000188130<br>ENSG00000162104<br>ENSG00000197943<br>ENSG00000164171<br>ENSG00000196914<br>ENSG00000121879<br>ENSG00000121281<br>ENSG00000243708<br>ENSG00000101608<br>ENSG00000067606	MYLK3 ITPI<br>MAPK3<br>MAPK12<br>ADCY9 PLC<br>ITGA2<br>ARHGEF1<br>PIK3CA<br>ADCY7<br>PLA2G4B<br>MYL12A<br>PRKCZ
hsa04622	RIG-I-like receptor signaling pathway	15/1188	54/5573	0.158769491658617	0.527047903000609	ENSG00000169429<br>ENSG00000083799<br>ENSG00000109320<br>ENSG00000168811<br>ENSG00000100906<br>ENSG00000131323<br>ENSG00000168040<br>ENSG00000185386<br>ENSG00000188130<br>ENSG00000187608<br>ENSG00000136560<br>ENSG00000104825<br>ENSG00000263528<br>ENSG00000184584<br>ENSG00000064012	CXCL8 CYL<br>NFKB1 IL12<br>NFKBIA<br>TRAF3 FAD<br>MAPK11<br>MAPK12<br>ISG15 TAN<br>NFKBIB IKBI<br>TMEM173<br>CASP8
hsa04666	Fc gamma R-mediated phagocytosis	20/1188	75/5573	0.159097180414047	0.527047903000609	ENSG00000176170<br>ENSG00000134215<br>ENSG00000148180<br>ENSG00000171132<br>ENSG00000132155<br>ENSG00000088280<br>ENSG00000136950<br>ENSG00000067113<br>ENSG00000141934<br>ENSG00000102882<br>ENSG00000172757<br>ENSG00000197943<br>ENSG00000006747<br>ENSG00000106683<br>ENSG00000143398<br>ENSG00000121879<br>ENSG00000153317<br>ENSG00000243708<br>ENSG00000145555<br>ENSG00000165527	SPHK1 VAV<br>GSN PRKC<br>RAF1 ASAP<br>ARPC5L<br>PLPP1 PLPI<br>MAPK3 CFL<br>PLCG2 SCI<br>LIMK1<br>PIP5K1A<br>PIK3CA<br>ASAP1<br>PLA2G4B<br>MYO10 ARI
hsa04370	VEGF signaling pathway	14/1188	50/5573	0.161572086807244	0.527047903000609	ENSG00000176170<br>ENSG00000073756<br>ENSG00000132155<br>ENSG00000174775<br>ENSG00000185386<br>ENSG00000197122<br>ENSG00000102882<br>ENSG00000188130<br>ENSG00000112715<br>ENSG00000106211<br>ENSG00000197943<br>ENSG00000114738<br>ENSG00000121879<br>ENSG00000243708	SPHK1 PTG<br>RAF1 HRA<br>MAPK11 SR<br>MAPK3<br>MAPK12<br>VEGFA HSPI<br>PLCG2<br>MAPKAPK<br>PIK3CA<br>PLA2G4B
hsa05414	Dilated cardiomyopathy (DCM)	18/1188	67/5573	0.166398945223157	0.527047903000609	ENSG00000105855<br>ENSG00000082781<br>ENSG00000184009<br>ENSG00000213949<br>ENSG00000174233<br>ENSG00000155657<br>ENSG00000043591<br>ENSG00000157445<br>ENSG00000173402<br>ENSG00000162104<br>ENSG00000175084<br>ENSG00000198947<br>ENSG00000198626<br>ENSG00000164171<br>ENSG00000160789<br>ENSG00000121281	ITGB8 ITGE<br>ACTG1 ITG<br>ADCY6 TTI<br>ADRB1<br>CACNA2D<br>DAG1 ADCY<br>DES DMD<br>RYR2 ITGA<br>LMNA ADCY<br>EMD ITGA

						ENSG00000102119<br>ENSG00000005884	
hsa03420	Nucleotide excision repair	12/1188	42/5573	0.166696539693234	0.527047903000609	ENSG00000115350<br>ENSG00000134574<br>ENSG00000148229<br>ENSG00000225830<br>ENSG00000179262<br>ENSG00000106628<br>ENSG00000104884<br>ENSG00000134480<br>ENSG00000100387<br>ENSG00000139842<br>ENSG00000062822<br>ENSG00000183474	POLE4 DDE<br>POLE3 ERC<br>RAD23A<br>POLD2 ERC<br>CCNH RBX<br>CUL4A POLI<br>GTF2H2C
hsa04150	mTOR signaling pathway	34/1188	136/5573	0.169065756385785	0.527047903000609	ENSG00000168209<br>ENSG00000082701<br>ENSG00000130766<br>ENSG00000114251<br>ENSG00000107404<br>ENSG00000188064<br>ENSG00000118515<br>ENSG00000155760<br>ENSG00000127580<br>ENSG00000177169<br>ENSG00000132155<br>ENSG00000174775<br>ENSG00000130779<br>ENSG00000168003<br>ENSG00000100485<br>ENSG00000180340<br>ENSG00000162337<br>ENSG00000186654<br>ENSG00000171105<br>ENSG00000100726<br>ENSG00000102882<br>ENSG00000164327<br>ENSG00000217128<br>ENSG00000169884<br>ENSG00000114388<br>ENSG00000115596<br>ENSG00000163251<br>ENSG00000116039<br>ENSG00000171862<br>ENSG00000121879<br>ENSG00000157240<br>ENSG00000114573<br>ENSG00000157764<br>ENSG00000187840	DDIT4 GSK<br>SESN2<br>WNT5A DVI<br>WNT7B SGI<br>FZD7 WDR<br>ULK1 RAF<br>HRAS CLIP<br>SLC3A2 SOS<br>FZD2 LRP<br>PRR5 INSF<br>TELO2 MAP<br>RICTOR FNI<br>WNT10B<br>NPRL2 WNT<br>FZD5<br>ATP6V1B1<br>PTEN PIK3<br>FZD1<br>ATP6V1A<br>BRAF<br>EIF4EBP1
hsa04630	JAK-STAT signaling pathway	28/1188	110/5573	0.169754006627054	0.527047903000609	ENSG00000136244<br>ENSG00000174564<br>ENSG00000162434<br>ENSG00000110092<br>ENSG00000112576<br>ENSG00000137193<br>ENSG00000142166<br>ENSG00000168811<br>ENSG00000027697<br>ENSG00000095752<br>ENSG00000005339<br>ENSG00000132155<br>ENSG00000104998<br>ENSG00000174775<br>ENSG00000100485<br>ENSG00000184557<br>ENSG00000033800<br>ENSG00000128342<br>ENSG00000120833<br>ENSG00000115415<br>ENSG00000100385<br>ENSG00000213218<br>ENSG00000164136<br>ENSG00000121879<br>ENSG00000242689<br>ENSG00000126561<br>ENSG00000078043<br>ENSG00000147168	IL6 IL20RE<br>JAK1 CCND<br>CCND3 PIM<br>IFNAR1 IL1<br>IFNGR1 IL1<br>CREBBP RA<br>IL27RA HR<br>SOS2 SOCS<br>PIAS1 LIF<br>SOCS2 STA<br>IL2RB CSH<br>IL15 PIK3C<br>CNTF STAT<br>PIAS2 IL2R
hsa00980	Metabolism of xenobiotics by cytochrome P450	10/1188	34/5573	0.170579713613723	0.527047903000609	ENSG00000143819<br>ENSG00000006534<br>ENSG00000143198<br>ENSG00000159228<br>ENSG00000085871	EPHX1<br>ALDH3B1<br>MGST3 CBF<br>MGST2<br>AKR1C1

						ENSG00000187134<br>ENSG00000197448<br>ENSG00000134184<br>ENSG00000167600<br>ENSG00000108602	GSTK1 GST<br>CYP2S1<br>ALDH3A1
hsa00640	Propanoate metabolism	9/1188	30/5573	0.171665315196086	0.527047903000609	ENSG00000083123<br>ENSG00000171989<br>ENSG00000111716<br>ENSG00000146085<br>ENSG00000120437<br>ENSG00000076555<br>ENSG00000084754<br>ENSG00000117054<br>ENSG00000119711	BCKDHB<br>LDHAL6B<br>LDHB MUT<br>ACAT2 ACA<br>HADHA<br>ACADM<br>ALDH6A1
hsa05144	Malaria	8/1188	26/5573	0.171804027639036	0.527047903000609	ENSG00000136244<br>ENSG00000123384<br>ENSG00000137801<br>ENSG00000169429<br>ENSG00000105976<br>ENSG00000168811<br>ENSG00000090339<br>ENSG00000110651	IL6 LRP1<br>THBS1 CXC<br>MET IL12<br>ICAM1 CD8
hsa05168	Herpes simplex infection	33/1188	132/5573	0.173393609781959	0.527047903000609	ENSG00000125730<br>ENSG00000136244<br>ENSG00000115875<br>ENSG00000109320<br>ENSG00000162434<br>ENSG00000177606<br>ENSG00000133794<br>ENSG00000142166<br>ENSG00000168811<br>ENSG00000027697<br>ENSG00000100906<br>ENSG00000005339<br>ENSG00000131323<br>ENSG00000099804<br>ENSG00000162231<br>ENSG00000181222<br>ENSG00000168040<br>ENSG00000184557<br>ENSG00000170345<br>ENSG00000165632<br>ENSG00000115415<br>ENSG00000111335<br>ENSG00000166337<br>ENSG00000108561<br>ENSG00000164136<br>ENSG00000104825<br>ENSG00000096063<br>ENSG00000263528<br>ENSG00000100324<br>ENSG00000159479<br>ENSG00000064012<br>ENSG00000141510<br>ENSG00000263001	C3 IL6 SRS<br>NFKB1 JAK<br>JUN ARNT<br>IFNAR1 IL1<br>IFNGR1<br>NFKBIA<br>CREBBP<br>TRAF3 CDC<br>NXF1 POLR<br>FADD SOCS<br>FOS TAF3<br>STAT1 OAS<br>TAF10 C1Q<br>IL15 NFKBI<br>SRPK1 IKBK<br>TAB1 MED<br>CASP8 TP5<br>GTF2I
hsa04915	Estrogen signaling pathway	25/1188	98/5573	0.183263278271239	0.547664130436135	ENSG00000150995<br>ENSG00000128272<br>ENSG00000080824<br>ENSG00000182158<br>ENSG00000004478<br>ENSG00000149782<br>ENSG00000166598<br>ENSG00000177606<br>ENSG00000132155<br>ENSG00000164850<br>ENSG00000173110<br>ENSG00000174775<br>ENSG00000100485<br>ENSG00000174233<br>ENSG00000096384<br>ENSG00000197122<br>ENSG00000096433<br>ENSG00000102882<br>ENSG00000162104<br>ENSG00000171345<br>ENSG00000170345<br>ENSG00000163235<br>ENSG00000124151<br>ENSG00000121879<br>ENSG00000121281	ITPR1 ATF<br>HSP90AA1<br>CREB3L2<br>FKBP4 PLC<br>HSP90B1 JU<br>RAF1 GPER<br>HSPA6 HRA<br>SOS2 ADCY<br>HSP90AB1<br>SRC ITPR3<br>MAPK3<br>ADCY9 KRT<br>FOS TGFA<br>NCOA3<br>PIK3CA<br>ADCY7
hsa04520	Adherens junction	18/1188	68/5573	0.183743991644697	0.547664130436135	ENSG00000166949	SMAD3 SNA

						ENSG00000124216 ENSG00000105976 ENSG00000140575 ENSG00000087095 ENSG00000142949 ENSG00000149177 ENSG00000184009 ENSG00000175387 ENSG00000198561 ENSG00000005339 ENSG00000148737 ENSG00000171105 ENSG00000197122 ENSG00000102882 ENSG00000035403 ENSG00000151422 ENSG00000136153	MET IQGAF NLK PTPRI PTPRJ ACT(SMAD2 CTNND1 CREBBP TCF7L2 INS SRC MAPK VCL FER LMO7
hsa04662	B cell receptor signaling pathway	16/1188	60/5573	0.192950191769141	0.567286448353374	ENSG00000082701 ENSG00000134215 ENSG00000109320 ENSG00000177606 ENSG00000146232 ENSG00000100906 ENSG00000132155 ENSG00000174775 ENSG00000100485 ENSG00000102882 ENSG00000170345 ENSG00000197943 ENSG00000105369 ENSG00000121879 ENSG00000110651 ENSG00000104825	GSK3B VAV NFKB1 JUI NFKBIE NFKBIA RAI HRAS SOS MAPK3 FO PLCG2 CD7 PIK3CA CD8 NFKBIB
hsa04714	Thermogenesis	50/1188	209/5573	0.1960008172424	0.567286448353374	ENSG00000198804 ENSG00000182158 ENSG00000184009 ENSG00000151366 ENSG00000099624 ENSG00000185633 ENSG00000198712 ENSG00000198786 ENSG00000179091 ENSG00000240230 ENSG00000110090 ENSG00000174775 ENSG00000109390 ENSG00000212907 ENSG00000131174 ENSG00000100485 ENSG00000174233 ENSG00000198938 ENSG00000140990 ENSG00000198886 ENSG00000181924 ENSG00000116985 ENSG00000184076 ENSG00000115286 ENSG00000168275 ENSG00000185386 ENSG00000099795 ENSG00000127540 ENSG00000117118 ENSG00000164258 ENSG00000188130 ENSG00000127184 ENSG00000162104 ENSG00000077463 ENSG00000162377 ENSG00000154723 ENSG00000119421 ENSG00000198840 ENSG00000197119 ENSG00000183648 ENSG00000121281 ENSG00000108604 ENSG00000124172 ENSG00000198695 ENSG00000134962 ENSG00000203667 ENSG00000166819 ENSG00000110717	MT-CO1 CREB3L2 ACTG1 NDUFC2 ATP5F1D NDUFA4L MT-CO2 MT ND5 CYC1 COX19 CPT HRAS NDUFC1 MT ND4L COX7 SOS2 ADCY MT-CO3 NDUFB10 M ND4 COA4 BMP8B UQCR10 NDUFS7 CO. MAPK11 NDUFB7 UQCR11 SDHB NDUF MAPK12 COX7C ADCY9 SIRT COA7 ATP5 NDUFA8 MT ND3 SLC25A29 NDUFB1 ADCY7 SMARCD2 ATP5F1E MT ND6 KLB COX20 PLIN NDUFS8 UQCRHL UQCRC2

						ENSG00000233954 ENSG00000140740	
hsa05221	Acute myeloid leukemia	15/1188	56/5573	0.197724203314944	0.567286448353374	ENSG00000173801 ENSG00000109320 ENSG00000110092 ENSG00000137193 ENSG00000159216 ENSG00000132155 ENSG00000174775 ENSG00000100485 ENSG00000148737 ENSG00000102882 ENSG00000121879 ENSG00000126561 ENSG00000157764 ENSG00000187840 ENSG00000102096	JUP NFKB CCND1 PIM RUNX1 RAF HRAS SOS TCF7L2 MAPK3 PIK3CA STAT5A BR EIF4EBP1 PIM2
hsa00250	Alanine, aspartate and glutamate metabolism	9/1188	31/5573	0.198922865022369	0.567286448353374	ENSG00000166532 ENSG00000185818 ENSG00000084774 ENSG00000021826 ENSG00000070669 ENSG00000166123 ENSG00000130707 ENSG00000185100 ENSG00000198380	RIMKLB NAT8L CA CPS1 ASN GPT2 ASS ADSSL1 GFPT1
hsa00360	Phenylalanine metabolism	5/1188	15/5573	0.199566568150373	0.567286448353374	ENSG00000158104 ENSG00000006534 ENSG00000131471 ENSG00000069535 ENSG00000108602	HPD ALDH3 AOC3 MAO ALDH3A1
hsa05143	African trypanosomiasis	7/1188	23/5573	0.20272202843443	0.570969382838256	ENSG00000136244 ENSG00000149782 ENSG00000172009 ENSG00000168811 ENSG00000090339 ENSG00000100342 ENSG00000164251	IL6 PLCB3 THOP1 IL1 ICAM1 APO F2RL1
hsa05164	Influenza A	29/1188	117/5573	0.206335105225315	0.575862520947016	ENSG00000136244 ENSG00000108984 ENSG00000169429 ENSG00000082701 ENSG00000109320 ENSG00000162434 ENSG00000177606 ENSG00000184009 ENSG00000142166 ENSG00000132002 ENSG00000168811 ENSG00000090339 ENSG00000027697 ENSG00000100906 ENSG00000005339 ENSG00000132155 ENSG00000173110 ENSG00000162231 ENSG00000102580 ENSG00000184557 ENSG00000185386 ENSG00000102882 ENSG00000188130 ENSG00000115415 ENSG00000111335 ENSG00000121879 ENSG00000065559 ENSG00000104825 ENSG00000263528	IL6 MAP2K CXCL8 GSK NFKB1 JAK JUN ACTG IFNAR1 DNAJB1 IL12A ICAM IFNGR1 NFKBIA CREBBP RA HSPA6 NXF DNAJC3 SOCS3 MAPK11 MAPK3 MAPK12 STAT1 OAS PIK3CA MAP2K4 NFKBIB IKB
hsa04062	Chemokine signaling pathway	31/1188	126/5573	0.209463503495726	0.579326987145838	ENSG00000169429 ENSG00000082701 ENSG00000115009 ENSG00000134215 ENSG00000109320 ENSG00000134318 ENSG00000149782 ENSG00000173020 ENSG00000105723 ENSG00000100906 ENSG00000132155 ENSG00000163739 ENSG00000174775	CXCL8 GSK CCL20 VAV NFKB1 ROC PLCB3 GRK GSK3A NFKBIA RA CXCL1 HRA SOS2 ADCY BCAR1 CCL SRC GNB2 MAPK3 ADCY9 PTK STAT1

						ENSG00000100485 ENSG00000174233 ENSG00000050820 ENSG00000151882 ENSG00000197122 ENSG00000172354 ENSG00000102882 ENSG00000162104 ENSG00000120899 ENSG00000115415 ENSG00000121879 ENSG00000121281 ENSG00000104825 ENSG00000067606 ENSG00000157764 ENSG00000137486 ENSG00000184451 ENSG00000169248	PIK3CA ADCY7 NFKBIB PRKCZ BRA ARRB1 CCR CXCL11
hsa03008	Ribosome biogenesis in eukaryotes	19/1188	74/5573	0.214959398549061	0.589219065665731	ENSG00000136718 ENSG00000183751 ENSG00000065183 ENSG00000178718 ENSG00000189306 ENSG00000241945 ENSG00000126749 ENSG00000132341 ENSG00000141101 ENSG00000162231 ENSG00000011260 ENSG00000165271 ENSG00000058729 ENSG00000041802 ENSG00000152464 ENSG00000101361 ENSG00000172336 ENSG00000070814 ENSG00000130826	IMP4 TBL3 WDR3 RPP2 RRP7A PWF EMG1 RAN NOB1 NXF UTP18 NOL RIOK2 LSG RPP38 NOP POP7 TCOF DKC1
hsa05133	Pertussis	15/1188	57/5573	0.218732275031037	0.594254941898481	ENSG00000125730 ENSG00000136244 ENSG00000169429 ENSG00000109320 ENSG00000177606 ENSG00000168811 ENSG00000184216 ENSG00000182326 ENSG00000159403 ENSG00000185386 ENSG00000102882 ENSG00000188130 ENSG00000170345 ENSG00000172757 ENSG00000106100	C3 IL6 CXCL NFKB1 JUN IL12A IRAK C1S C1R MAPK11 MAPK3 MAPK12 FC CFL1 NOD
hsa01521	EGFR tyrosine kinase inhibitor resistance	18/1188	70/5573	0.220983899986165	0.595105765752215	ENSG00000136244 ENSG00000082701 ENSG00000167601 ENSG00000105976 ENSG00000162434 ENSG00000132155 ENSG00000174775 ENSG00000100485 ENSG00000068078 ENSG00000197122 ENSG00000102882 ENSG00000112715 ENSG00000197943 ENSG00000163235 ENSG00000171862 ENSG00000121879 ENSG00000157764 ENSG00000187840	IL6 GSK3E AXL MET JAK1 RAF HRAS SOS FGFR3 SRC MAPK3 VEGFA PLC TGFA PTEI PIK3CA BRA EIF4EBP1
hsa04970	Salivary secretion	14/1188	53/5573	0.224737326982852	0.597851276242852	ENSG00000150995 ENSG00000143153 ENSG00000149782 ENSG00000164889 ENSG00000174233 ENSG00000104783 ENSG00000043591 ENSG00000058668 ENSG00000070961 ENSG00000096433 ENSG00000162104	ITPR1 ATP1B1 PLCB3 SLC4A2 ADCY6 KCNN4 ADRB1 ATP2B4 ATP2B1 ITPR3 ADCY

						ENSG00000121281 ENSG00000161798 ENSG00000198838	ADCY7 AQ[illegible] RYR3
hsa04612	Antigen processing and presentation	9/1188	32/5573	0.227845600392227	0.597851276242852	ENSG00000080824 ENSG00000092010 ENSG00000164733 ENSG00000179218 ENSG00000167004 ENSG00000173110 ENSG00000096384 ENSG00000143390 ENSG00000135047	HSP90AA1 PSME1 CTS[illegible] CALR PDIA[illegible] HSPA6 HSP90AB1 RFX5 CTS[illegible]
hsa04913	Ovarian steroidogenesis	9/1188	32/5573	0.227845600392227	0.597851276242852	ENSG00000130164 ENSG00000196139 ENSG00000073756 ENSG00000174233 ENSG00000171105 ENSG00000162104 ENSG00000140459 ENSG00000121281 ENSG00000243708	LDLR AKR1[illegible] PTGS2 ADC[illegible] INSR ADCY[illegible] CYP11A1 ADCY7 PLA2G4B
hsa04960	Aldosterone-regulated sodium reabsorption	8/1188	28/5573	0.232655692887	0.60529913318906	ENSG00000143153 ENSG00000049759 ENSG00000065054 ENSG00000118515 ENSG00000171105 ENSG00000102882 ENSG00000121879	ATP1B1 NEDD4L SLC9A3R2 SGK1 SCNN[illegible] INSR MAPK[illegible] PIK3CA
hsa04916	Melanogenesis	21/1188	84/5573	0.239170829190022	0.605561022616364	ENSG00000082701 ENSG00000114251 ENSG00000107404 ENSG00000182158 ENSG00000149782 ENSG00000188064 ENSG00000155760 ENSG00000005339 ENSG00000132155 ENSG00000174775 ENSG00000174233 ENSG00000148737 ENSG00000180340 ENSG00000187098 ENSG00000102882 ENSG00000162104 ENSG00000169884 ENSG00000115596 ENSG00000163251 ENSG00000157240 ENSG00000121281	GSK3B WNT5A DV[illegible] CREB3L2 PLCB3 WNT7B FZ[illegible] CREBBP RA[illegible] HRAS ADCY[illegible] TCF7L2 FZ[illegible] MITF MAPK[illegible] ADCY9 WNT10B WNT6 FZD[illegible] FZD1 ADCY[illegible]
hsa05215	Prostate cancer	21/1188	84/5573	0.239170829190022	0.605561022616364	ENSG00000082701 ENSG00000128272 ENSG00000080824 ENSG00000109320 ENSG00000182158 ENSG00000110092 ENSG00000166598 ENSG00000100906 ENSG00000005339 ENSG00000132155 ENSG00000174775 ENSG00000100485 ENSG00000096384 ENSG00000148737 ENSG00000102882 ENSG00000163235 ENSG00000171862 ENSG00000121879 ENSG00000112242 ENSG00000157764 ENSG00000141510	GSK3B ATF[illegible] HSP90AA1 NFKB1 CREB3L2 CCND1 HSP90B1 NFKBIA CREBBP RA[illegible] HRAS SOS[illegible] HSP90AB1 TCF7L2 MAPK3 TG[illegible] PTEN PIK3[illegible] E2F3 BRA[illegible] TP53
hsa05412	Arrhythmogenic right ventricular cardiomyopathy (ARVC)	15/1188	58/5573	0.240658212099482	0.605561022616364	ENSG00000105855 ENSG00000173801 ENSG00000082781 ENSG00000184009 ENSG00000213949 ENSG00000148737 ENSG00000157445 ENSG00000173402 ENSG00000175084	ITGB8 JU[illegible] ITGB5 ACT[illegible] ITGA1 TCF7L2 CACNA2D[illegible] DAG1 DES[illegible] DMD RYR[illegible] ITGA2 LMN[illegible] EMD ITGA[illegible]

						ENSG00000198947 ENSG00000198626 ENSG00000164171 ENSG00000160789 ENSG00000102119 ENSG00000005884	
hsa00670	One carbon pool by folate	5/1188	16/5573	0.242589887419026	0.605561022616364	ENSG00000228716 ENSG00000136371 ENSG00000065911 ENSG00000116984 ENSG00000100714	DHFR MTHI MTHFD2 MT MTHFD1
hsa04723	Retrograde endocannabinoid signaling	28/1188	115/5573	0.24261891134141	0.605561022616364	ENSG00000150995 ENSG00000149782 ENSG00000151366 ENSG00000073756 ENSG00000185633 ENSG00000198786 ENSG00000109390 ENSG00000212907 ENSG00000174233 ENSG00000140990 ENSG00000198886 ENSG00000115286 ENSG00000185386 ENSG00000099795 ENSG00000096433 ENSG00000172354 ENSG00000102882 ENSG00000164258 ENSG00000188130 ENSG00000162104 ENSG00000119421 ENSG00000198840 ENSG00000183648 ENSG00000121281 ENSG00000102287 ENSG00000164535 ENSG00000198695 ENSG00000110717	ITPR1 PLCE NDUFC2 PTGS2 NDUFA4L2 MT-ND5 NDUFC1 MT ND4L ADCY NDUFB10 M ND4 NDUFS MAPK11 NDUFB7 ITPR3 GNB MAPK3 NDUFS4 MAPK12 ADCY9 NDUFA8 MT ND3 NDUFE ADCY7 GABRE DAGLB MT ND6 NDUFS
hsa05418	Fluid shear stress and atherosclerosis	28/1188	116/5573	0.258658038217017	0.640387239779228	ENSG00000124145 ENSG00000108984 ENSG00000080824 ENSG00000109320 ENSG00000166598 ENSG00000177606 ENSG00000184009 ENSG00000090339 ENSG00000120129 ENSG00000143198 ENSG00000096384 ENSG00000085871 ENSG00000185386 ENSG00000197122 ENSG00000188130 ENSG00000116044 ENSG00000112715 ENSG00000170345 ENSG00000116701 ENSG00000135047 ENSG00000134184 ENSG00000130707 ENSG00000107779 ENSG00000121879 ENSG00000065559 ENSG00000007952 ENSG00000067606 ENSG00000141510	SDC4 MAP2I HSP90AA1 NFKB1 HSP90B1 JU ACTG1 ICAI DUSP1 MGS HSP90AB1 MGST2 MAPK11 SR MAPK12 NFE2L2 VEGFA FO NCF2 CTSI GSTM1 ASS BMPR1A PIK3CA MAP2K4 NOX1 PRKC TP53
hsa04540	Gap junction	18/1188	72/5573	0.261257904614699	0.641649413733702	ENSG00000150995 ENSG00000188229 ENSG00000149782 ENSG00000127824 ENSG00000137267 ENSG00000132155 ENSG00000176014 ENSG00000174775 ENSG00000100485 ENSG00000174233 ENSG00000123416 ENSG00000043591 ENSG00000197122 ENSG00000096433	ITPR1 TUBB4B PLCB3 TUBA4A TUBB2A RA TUBB6 HRA SOS2 ADCY TUBA1B ADRB1 SRC ITPR3 MAPI ADCY9 TUBA1C ADCY7

						ENSG00000102882 ENSG00000162104 ENSG00000167553 ENSG00000121281	
hsa04145	Phagosome	25/1188	103/5573	0.263924861313736	0.643055019232674	ENSG00000125730 ENSG00000173391 ENSG00000137801 ENSG00000188229 ENSG00000117410 ENSG00000082781 ENSG00000127824 ENSG00000184009 ENSG00000137267 ENSG00000179218 ENSG00000153246 ENSG00000176014 ENSG00000111540 ENSG00000123416 ENSG00000159403 ENSG00000132432 ENSG00000116701 ENSG00000110719 ENSG00000135047 ENSG00000167553 ENSG00000164171 ENSG00000116039 ENSG00000005893 ENSG00000117758 ENSG00000114573	C3 OLR1 THBS1 TUBB4B ATP6V0B ITGB5 TUBA4A ACTG1 TUBB2A CA PLA2R1 TUBB6 RAB TUBA1B C1 SEC61G NC TCIRG1 CT TUBA1C ITGA2 ATP6V1B1 LAMP2 STX ATP6V1A
hsa00330	Arginine and proline metabolism	11/1188	42/5573	0.271586962384536	0.656513365764194	ENSG00000115758 ENSG00000116649 ENSG00000122884 ENSG00000081181 ENSG00000133313 ENSG00000164904 ENSG00000143811 ENSG00000072682 ENSG00000069535 ENSG00000100033 ENSG00000183010	ODC1 SRM P4HA1 ARG CNDP2 ALDH7A1 PYCR2 P4H MAOB PROD PYCR1
hsa04918	Thyroid hormone synthesis	13/1188	51/5573	0.280601120990367	0.672000409871236	ENSG00000150995 ENSG00000128272 ENSG00000143153 ENSG00000182158 ENSG00000149782 ENSG00000166598 ENSG00000174233 ENSG00000155660 ENSG00000125618 ENSG00000096433 ENSG00000233276 ENSG00000162104 ENSG00000121281	ITPR1 ATF ATP1B1 CREB3L2 PLCB3 HSP90B1 ADCY6 PDI PAX8 ITPR GPX1 ADCY ADCY7
hsa04211	Longevity regulating pathway	18/1188	73/5573	0.28237150773091	0.672000409871236	ENSG00000128272 ENSG00000130766 ENSG00000112096 ENSG00000109320 ENSG00000182158 ENSG00000177169 ENSG00000174775 ENSG00000175224 ENSG00000174233 ENSG00000171105 ENSG00000123395 ENSG00000162104 ENSG00000121879 ENSG00000121281 ENSG00000110931 ENSG00000121691 ENSG00000187840 ENSG00000141510	ATF4 SESN SOD2 NFKE CREB3L2 ULK1 HRA ATG13 ADC INSR ATG1 ADCY9 PIK3CA ADCY7 CAMKK2 C EIF4EBP1 TP53
hsa04912	GnRH signaling pathway	20/1188	82/5573	0.285675940681025	0.674634721454421	ENSG00000150995 ENSG00000108984 ENSG00000128272 ENSG00000149782 ENSG00000177606 ENSG00000132155 ENSG00000174775 ENSG00000100485 ENSG00000174233 ENSG00000185386	ITPR1 MAP2K6 AT PLCB3 JU RAF1 HRA SOS2 ADCY MAPK11 SR ITPR3 MAP MAPK12 ADCY9 PTK GNRH1

						ENSG00000197122<br>ENSG00000096433<br>ENSG00000102882<br>ENSG00000188130<br>ENSG00000162104<br>ENSG00000120899<br>ENSG00000147437<br>ENSG00000121281<br>ENSG00000065559<br>ENSG00000243708	ADCY7<br>MAP2K4<br>PLA2G4B
hsa03020	RNA polymerase	8/1188	30/5573	0.299706500524127	0.699874620969842	ENSG00000113356<br>ENSG00000102978<br>ENSG00000161980<br>ENSG00000125630<br>ENSG00000181222<br>ENSG00000285437<br>ENSG00000058600<br>ENSG00000099817	POLR3G<br>POLR2C<br>POLR3K<br>POLR1B<br>POLR2A<br>POLR2J3<br>POLR3E<br>POLR2E
hsa05211	Renal cell carcinoma	16/1188	65/5573	0.300923289798108	0.699874620969842	ENSG00000105976<br>ENSG00000177606<br>ENSG00000116016<br>ENSG00000005339<br>ENSG00000132155<br>ENSG00000174775<br>ENSG00000100485<br>ENSG00000135766<br>ENSG00000102882<br>ENSG00000112715<br>ENSG00000100644<br>ENSG00000117394<br>ENSG00000163235<br>ENSG00000121879<br>ENSG00000100387<br>ENSG00000157764	MET JUN<br>EPAS1<br>CREBBP RAI<br>HRAS SOS2<br>EGLN1<br>MAPK3<br>VEGFA HIF1<br>SLC2A1 TGI<br>PIK3CA RBX<br>BRAF
hsa04974	Protein digestion and absorption	15/1188	61/5573	0.311018627625035	0.716508664483417	ENSG00000111799<br>ENSG00000130635<br>ENSG00000143153<br>ENSG00000108821<br>ENSG00000168542<br>ENSG00000105281<br>ENSG00000134294<br>ENSG00000188153<br>ENSG00000168003<br>ENSG00000092758<br>ENSG00000104783<br>ENSG00000182871<br>ENSG00000197565<br>ENSG00000142156<br>ENSG00000164626	COL12A1<br>COL5A1<br>ATP1B1<br>COL1A1<br>COL3A1<br>SLC1A5<br>SLC38A2<br>COL4A5<br>SLC3A2<br>COL9A3<br>KCNN4<br>COL18A1<br>COL4A6<br>COL6A1<br>KCNK5
hsa05016	Huntington disease	41/1188	178/5573	0.312743195572566	0.716508664483417	ENSG00000150995<br>ENSG00000198804<br>ENSG00000112096<br>ENSG00000182158<br>ENSG00000149782<br>ENSG00000102978<br>ENSG00000151366<br>ENSG00000070371<br>ENSG00000169375<br>ENSG00000099624<br>ENSG00000185633<br>ENSG00000198712<br>ENSG00000179091<br>ENSG00000005339<br>ENSG00000181222<br>ENSG00000109390<br>ENSG00000131174<br>ENSG00000198938<br>ENSG00000140990<br>ENSG00000184076<br>ENSG00000115286<br>ENSG00000285437<br>ENSG00000175416<br>ENSG00000099795<br>ENSG00000127540<br>ENSG00000117118<br>ENSG00000164258<br>ENSG00000233276<br>ENSG00000127184<br>ENSG00000161203<br>ENSG00000154723<br>ENSG00000119421	ITPR1 MT-<br>CO1 SOD2<br>CREB3L2<br>PLCB3<br>POLR2C<br>NDUFC2<br>CLTCL1<br>SIN3A<br>ATP5F1D<br>NDUFA4L2<br>MT-CO2 CY<br>CREBBP<br>POLR2A<br>NDUFC1<br>COX7B MT<br>CO3<br>NDUFB10<br>UQCR10<br>NDUFS7<br>POLR2J3<br>CLTB NDUF<br>UQCR11<br>SDHB NDUF<br>GPX1 COX7<br>AP2M1<br>ATP5PF<br>NDUFA8<br>NDUFB1<br>POLR2E<br>ATP5F1E<br>TFAM<br>NDUFS8

						ENSG00000183648<br>ENSG00000099817<br>ENSG00000124172<br>ENSG00000108064<br>ENSG00000110717<br>ENSG00000233954<br>ENSG00000064012<br>ENSG00000141510<br>ENSG00000140740	UQCRHL<br>CASP8 TP5<br>UQCRC2
hsa01040	Biosynthesis of unsaturated fatty acids	6/1188	22/5573	0.321822169407309	0.731847451911437	ENSG00000099194<br>ENSG00000012660<br>ENSG00000115425<br>ENSG00000084754<br>ENSG00000097021<br>ENSG00000184227	SCD ELOVL<br>PECR HADH<br>ACOT7<br>ACOT1
hsa04722	Neurotrophin signaling pathway	25/1188	107/5573	0.336841878189829	0.760371004443216	ENSG00000134070<br>ENSG00000082701<br>ENSG00000128272<br>ENSG00000007264<br>ENSG00000109320<br>ENSG00000177606<br>ENSG00000146232<br>ENSG00000100906<br>ENSG00000132155<br>ENSG00000184216<br>ENSG00000080815<br>ENSG00000174775<br>ENSG00000100485<br>ENSG00000100784<br>ENSG00000134243<br>ENSG00000185386<br>ENSG00000141522<br>ENSG00000102882<br>ENSG00000188130<br>ENSG00000197943<br>ENSG00000121879<br>ENSG00000104825<br>ENSG00000160999<br>ENSG00000157764<br>ENSG00000141510	IRAK2 GSK3<br>ATF4 MATI<br>NFKB1 JUN<br>NFKBIE<br>NFKBIA RAF<br>IRAK1 PSEN<br>HRAS SOS2<br>RPS6KA5<br>SORT1<br>MAPK11<br>ARHGDIA<br>MAPK3<br>MAPK12<br>PLCG2<br>PIK3CA<br>NFKBIB<br>SH2B2 BRA<br>TP53
hsa04961	Endocrine and other factor-regulated calcium reabsorption	9/1188	36/5573	0.355349005492774	0.791714028755622	ENSG00000143153<br>ENSG00000149782<br>ENSG00000070371<br>ENSG00000174233<br>ENSG00000197959<br>ENSG00000175416<br>ENSG00000070961<br>ENSG00000162104<br>ENSG00000161203	ATP1B1<br>PLCB3<br>CLTCL1<br>ADCY6 DNM<br>CLTB ATP2I<br>ADCY9 AP2/
hsa05160	Hepatitis C	25/1188	108/5573	0.355884481981354	0.791714028755622	ENSG00000130164<br>ENSG00000169429<br>ENSG00000082701<br>ENSG00000163347<br>ENSG00000109320<br>ENSG00000162434<br>ENSG00000142166<br>ENSG00000100906<br>ENSG00000132155<br>ENSG00000131323<br>ENSG00000174775<br>ENSG00000100485<br>ENSG00000184557<br>ENSG00000185386<br>ENSG00000033800<br>ENSG00000102882<br>ENSG00000188130<br>ENSG00000181885<br>ENSG00000115415<br>ENSG00000111335<br>ENSG00000121879<br>ENSG00000110651<br>ENSG00000263528<br>ENSG00000157764<br>ENSG00000141510	LDLR CXCL<br>GSK3B CLDI<br>NFKB1 JAK<br>IFNAR1<br>NFKBIA RAF<br>TRAF3 HRA<br>SOS2 SOCS<br>MAPK11<br>PIAS1 MAPK<br>MAPK12<br>CLDN7 STAT<br>OAS2 PIK3C<br>CD81 IKBK<br>BRAF TP53
hsa04914	Progesterone-mediated oocyte maturation	20/1188	86/5573	0.370037802062063	0.807182039659756	ENSG00000080824<br>ENSG00000127564<br>ENSG00000132155<br>ENSG00000176386<br>ENSG00000174233<br>ENSG00000096384<br>ENSG00000166851	HSP90AA1<br>PKMYT1 RA<br>CDC26 ADC<br>HSP90AB1<br>PLK1 MAPK<br>CCNB2<br>MAPK3

						ENSG00000185386<br>ENSG00000157456<br>ENSG00000102882<br>ENSG00000188130<br>ENSG00000162104<br>ENSG00000107864<br>ENSG00000141552<br>ENSG00000116670<br>ENSG00000121879<br>ENSG00000121281<br>ENSG00000164109<br>ENSG00000164045<br>ENSG00000157764	MAPK12<br>ADCY9 CPE<br>ANAPC11<br>MAD2L2<br>PIK3CA<br>ADCY7<br>MAD2L1<br>CDC25A BR
hsa04750	Inflammatory mediator regulation of TRP channels	18/1188	77/5573	0.371584527976532	0.807182039659756	ENSG00000150995<br>ENSG00000108984<br>ENSG00000110881<br>ENSG00000149782<br>ENSG00000175591<br>ENSG00000171132<br>ENSG00000167723<br>ENSG00000174233<br>ENSG00000185386<br>ENSG00000197122<br>ENSG00000096433<br>ENSG00000188130<br>ENSG00000162104<br>ENSG00000197943<br>ENSG00000121879<br>ENSG00000121281<br>ENSG00000164251<br>ENSG00000243708	ITPR1<br>MAP2K6<br>ASIC1 PLC<br>P2RY2 PRK<br>TRPV3 ADC<br>MAPK11 SR<br>ITPR3<br>MAPK12<br>ADCY9 PLC<br>PIK3CA<br>ADCY7 F2R<br>PLA2G4B
hsa04010	MAPK signaling pathway	56/1188	251/5573	0.371921642568696	0.807182039659756	ENSG00000077150<br>ENSG00000108984<br>ENSG00000158050<br>ENSG00000169242<br>ENSG00000128272<br>ENSG00000164086<br>ENSG00000116717<br>ENSG00000162302<br>ENSG00000104856<br>ENSG00000109320<br>ENSG00000105976<br>ENSG00000087095<br>ENSG00000177606<br>ENSG00000099860<br>ENSG00000120129<br>ENSG00000160867<br>ENSG00000132155<br>ENSG00000184216<br>ENSG00000142627<br>ENSG00000173110<br>ENSG00000174775<br>ENSG00000109756<br>ENSG00000099875<br>ENSG00000100485<br>ENSG00000068078<br>ENSG00000100784<br>ENSG00000101109<br>ENSG00000117632<br>ENSG00000130159<br>ENSG00000120875<br>ENSG00000124882<br>ENSG00000185386<br>ENSG00000157445<br>ENSG00000171105<br>ENSG00000175197<br>ENSG00000102882<br>ENSG00000188130<br>ENSG00000173511<br>ENSG00000196924<br>ENSG00000112715<br>ENSG00000170345<br>ENSG00000106211<br>ENSG00000130829<br>ENSG00000114738<br>ENSG00000184371<br>ENSG00000163235<br>ENSG00000160551<br>ENSG00000065559<br>ENSG00000149930<br>ENSG00000243708<br>ENSG00000100324	NFKB2<br>MAP2K6<br>DUSP2 EFN<br>ATF4 DUSP<br>GADD45A<br>RPS6KA4<br>RELB NFKB<br>MET NLK JU<br>GADD45B<br>DUSP1 FGF<br>RAF1 IRAK<br>EPHA2 HSP<br>HRAS<br>RAPGEF2<br>MKNK2 SOS<br>FGFR3<br>RPS6KA5<br>STK4 STMN<br>ECSIT DUSP<br>EREG MAPK<br>CACNA2D<br>INSR DDIT<br>MAPK3<br>MAPK12<br>VEGFB FLN<br>VEGFA FO<br>HSPB1 DUS<br>MAPKAPK<br>CSF1 TGF<br>TAOK1<br>MAP2K4<br>TAOK2<br>PLA2G4B<br>TAB1 BRA<br>ARRB1 FLN<br>NR4A1 TP5

						ENSG00000157764<br>ENSG00000137486<br>ENSG00000128591<br>ENSG00000123358<br>ENSG00000141510	
hsa05032	Morphine addiction	14/1188	59/5573	0.373729379188082	0.807182039659756	ENSG00000113448<br>ENSG00000172572<br>ENSG00000112541<br>ENSG00000171408<br>ENSG00000173020<br>ENSG00000174233<br>ENSG00000073417<br>ENSG00000065989<br>ENSG00000172354<br>ENSG00000162104<br>ENSG00000121281<br>ENSG00000115252<br>ENSG00000102287<br>ENSG00000137486	PDE4D PDE<br>PDE10A<br>PDE7B GRK<br>ADCY6 PDE<br>PDE4A GNE<br>ADCY9 ADC<br>PDE1A GAB<br>ARRB1
hsa04931	Insulin resistance	21/1188	91/5573	0.379799238072944	0.807182039659756	ENSG00000136244<br>ENSG00000082701<br>ENSG00000167114<br>ENSG00000101255<br>ENSG00000109320<br>ENSG00000182158<br>ENSG00000142949<br>ENSG00000100906<br>ENSG00000171132<br>ENSG00000110090<br>ENSG00000184557<br>ENSG00000072310<br>ENSG00000171105<br>ENSG00000136111<br>ENSG00000076555<br>ENSG00000117394<br>ENSG00000171862<br>ENSG00000121879<br>ENSG00000119938<br>ENSG00000198380<br>ENSG00000067606	IL6 GSK3E<br>SLC27A4<br>TRIB3 NFKE<br>CREB3L2<br>PTPRF<br>NFKBIA<br>PRKCE CPT<br>SOCS3<br>SREBF1 INS<br>TBC1D4<br>ACACB<br>SLC2A1 PTE<br>PIK3CA<br>PPP1R3C<br>GFPT1 PRK
hsa00340	Histidine metabolism	5/1188	19/5573	0.381242331435389	0.807182039659756	ENSG00000006534<br>ENSG00000133313<br>ENSG00000164904<br>ENSG00000069535<br>ENSG00000108602	ALDH3B1<br>CNDP2<br>ALDH7A1<br>MAOB<br>ALDH3A1
hsa00532	Glycosaminoglycan biosynthesis - chondroitin sulfate / dermatan sulfate	5/1188	19/5573	0.381242331435389	0.807182039659756	ENSG00000122863<br>ENSG00000015532<br>ENSG00000182022<br>ENSG00000111962<br>ENSG00000123989	CHST3 XYL<br>CHST15 US<br>CHPF
hsa00983	Drug metabolism - other enzymes	11/1188	46/5573	0.388747668040592	0.80861151516974	ENSG00000158825<br>ENSG00000178035<br>ENSG00000183696<br>ENSG00000106348<br>ENSG00000143198<br>ENSG00000243678<br>ENSG00000085871<br>ENSG00000103024<br>ENSG00000025708<br>ENSG00000134184<br>ENSG00000137364	CDA IMPDH<br>UPP1 IMPD<br>MGST3 NME<br>MGST2 NME<br>TYMP GSTM<br>TPMT
hsa05152	Tuberculosis	28/1188	124/5573	0.39913917514802	0.80861151516974	ENSG00000125730<br>ENSG00000136244<br>ENSG00000176170<br>ENSG00000134070<br>ENSG00000172216<br>ENSG00000109320<br>ENSG00000117410<br>ENSG00000144381<br>ENSG00000162434<br>ENSG00000153879<br>ENSG00000168811<br>ENSG00000027697<br>ENSG00000005339<br>ENSG00000132155<br>ENSG00000184216<br>ENSG00000153246<br>ENSG00000168040<br>ENSG00000111540<br>ENSG00000185386	C3 IL6 SPH<br>IRAK2 CEBF<br>NFKB1<br>ATP6V0B<br>HSPD1 JAK<br>CEBPG IL12<br>IFNGR1<br>CREBBP RA<br>IRAK1<br>PLA2R1 FAD<br>RAB5B<br>MAPK11 SR<br>MAPK3<br>MAPK12 RF<br>STAT1<br>TCIRG1<br>ARHGEF12<br>LAMP2 CAS

						ENSG00000197122 ENSG00000102882 ENSG00000188130 ENSG00000143390 ENSG00000115415 ENSG00000110719 ENSG00000196914 ENSG00000005893 ENSG00000064012	
hsa00040	Pentose and glucuronate interconversions	4/1188	15/5573	0.401529075325353	0.80861151516974	ENSG00000085662 ENSG00000109814 ENSG00000165475 ENSG00000140263	AKR1B1 UGDH CRYl SORD
hsa00730	Thiamine metabolism	4/1188	15/5573	0.401529075325353	0.80861151516974	ENSG00000163283 ENSG00000163286 ENSG00000163295 ENSG00000140057	ALPP ALP(ALPI AK7
hsa04072	Phospholipase D signaling pathway	26/1188	115/5573	0.402609986520786	0.80861151516974	ENSG00000176170 ENSG00000169429 ENSG00000149091 ENSG00000149782 ENSG00000132155 ENSG00000144118 ENSG00000174775 ENSG00000100485 ENSG00000174233 ENSG00000197959 ENSG00000067113 ENSG00000171105 ENSG00000141934 ENSG00000102882 ENSG00000162104 ENSG00000120899 ENSG00000197943 ENSG00000008256 ENSG00000079337 ENSG00000143398 ENSG00000121879 ENSG00000143761 ENSG00000121281 ENSG00000169692 ENSG00000243708 ENSG00000165527	SPHK1 CXCl DGKZ PLCE RAF1 RALE HRAS SOS: ADCY6 DNM PLPP1 INSI PLPP2 MAPI ADCY9 PTK: PLCG2 CYTI RAPGEF3 PIP5K1A PIK3CA ARI ADCY7 AGPAT2 PLA2G4B ARF6
hsa05130	Pathogenic Escherichia coli infection	12/1188	51/5573	0.402680616222708	0.80861151516974	ENSG00000188229 ENSG00000163347 ENSG00000134318 ENSG00000127824 ENSG00000184009 ENSG00000115053 ENSG00000137267 ENSG00000176014 ENSG00000123416 ENSG00000136950 ENSG00000187554 ENSG00000167553	TUBB4B CLDN1 ROCK2 TUBA4A ACTG1 NC TUBB2A TUBB6 TUBA1B ARPC5L TLI TUBA1C
hsa04623	Cytosolic DNA-sensing pathway	10/1188	42/5573	0.404853672496411	0.80861151516974	ENSG00000136244 ENSG00000113356 ENSG00000109320 ENSG00000161980 ENSG00000100906 ENSG00000058600 ENSG00000099817 ENSG00000104825 ENSG00000263528 ENSG00000184584	IL6 POLR3(NFKB1 POLR3K NFKBIA POLR3E POLR2E NFKBIB IKBI TMEM173
hsa03410	Base excision repair	8/1188	33/5573	0.40630328222387	0.80861151516974	ENSG00000115350 ENSG00000005156 ENSG00000148229 ENSG00000106628 ENSG00000189403 ENSG00000041880 ENSG00000062822 ENSG00000114026	POLE4 LIG POLE3 POLI HMGB1 PARP3 POLI OGG1
hsa04660	T cell receptor signaling pathway	17/1188	74/5573	0.408034084441562	0.80861151516974	ENSG00000082701 ENSG00000134215 ENSG00000109320 ENSG00000177606 ENSG00000146232 ENSG00000100906 ENSG00000132155	GSK3B VAV NFKB1 JUI NFKBIE NFKBIA RAI CDK4 HRA SOS2 MAPK DLG1 MAPK

						ENSG00000135446<br>ENSG00000174775<br>ENSG00000100485<br>ENSG00000185386<br>ENSG00000075711<br>ENSG00000102882<br>ENSG00000188130<br>ENSG00000170345<br>ENSG00000121879<br>ENSG00000104825	MAPK12 FC<br>PIK3CA<br>NFKBIB
hsa05203	Viral carcinogenesis	38/1188	171/5573	0.414885477928796	0.80861151516974	ENSG00000125730<br>ENSG00000077150<br>ENSG00000128272<br>ENSG00000117399<br>ENSG00000108773<br>ENSG00000109320<br>ENSG00000182158<br>ENSG00000105810<br>ENSG00000162434<br>ENSG00000270276<br>ENSG00000110092<br>ENSG00000112576<br>ENSG00000177606<br>ENSG00000148180<br>ENSG00000100906<br>ENSG00000005339<br>ENSG00000131323<br>ENSG00000135446<br>ENSG00000174775<br>ENSG00000030110<br>ENSG00000147889<br>ENSG00000111642<br>ENSG00000184678<br>ENSG00000075711<br>ENSG00000197122<br>ENSG00000102882<br>ENSG00000141682<br>ENSG00000128245<br>ENSG00000061273<br>ENSG00000006747<br>ENSG00000121879<br>ENSG00000147099<br>ENSG00000158373<br>ENSG00000103479<br>ENSG00000126561<br>ENSG00000183474<br>ENSG00000064012<br>ENSG00000141510	C3 NFKB2<br>ATF4 CDC2<br>KAT2A NFKI<br>CREB3L2<br>CDK6 JAK<br>HIST2H4B<br>CCND1<br>CCND3 JUI<br>GSN NFKBI<br>CREBBP<br>TRAF3 CDK<br>HRAS BAK<br>CDKN2A<br>CHD4<br>HIST2H2BI<br>DLG1 SRC<br>MAPK3<br>PMAIP1<br>YWHAH<br>HDAC7 SCI<br>PIK3CA<br>HDAC8<br>HIST1H2BI<br>RBL2 STAT5<br>GTF2H2C<br>CASP8 TP5
hsa00590	Arachidonic acid metabolism	9/1188	38/5573	0.422539904699296	0.80861151516974	ENSG00000196139<br>ENSG00000095303<br>ENSG00000148334<br>ENSG00000073756<br>ENSG00000159228<br>ENSG00000148344<br>ENSG00000233276<br>ENSG00000110958<br>ENSG00000243708	AKR1C3<br>PTGS1<br>PTGES2<br>PTGS2 CBR<br>PTGES GPX<br>PTGES3<br>PLA2G4B
hsa05204	Chemical carcinogenesis	9/1188	38/5573	0.422539904699296	0.80861151516974	ENSG00000143819<br>ENSG00000073756<br>ENSG00000006534<br>ENSG00000143198<br>ENSG00000159228<br>ENSG00000085871<br>ENSG00000197448<br>ENSG00000134184<br>ENSG00000108602	EPHX1 PTG<br>ALDH3B1<br>MGST3 CBR<br>MGST2 GST<br>GSTM1<br>ALDH3A1
hsa00062	Fatty acid elongation	7/1188	29/5573	0.425846763936206	0.80861151516974	ENSG00000107362<br>ENSG00000129968<br>ENSG00000066322<br>ENSG00000012660<br>ENSG00000084754<br>ENSG00000097021<br>ENSG00000184227	ABHD17B<br>ABHD17A<br>ELOVL1<br>ELOVL5<br>HADHA<br>ACOT7<br>ACOT1
hsa00982	Drug metabolism - cytochrome P450	7/1188	29/5573	0.425846763936206	0.80861151516974	ENSG00000006534<br>ENSG00000143198<br>ENSG00000085871<br>ENSG00000197448<br>ENSG00000069535<br>ENSG00000134184<br>ENSG00000108602	ALDH3B1<br>MGST3<br>MGST2 GST<br>MAOB GSTM<br>ALDH3A1

hsa04940	Type I diabetes mellitus	3/1188	11/5573	0.426311858525028	0.80861151516974	ENSG00000144381<br>ENSG00000168811<br>ENSG00000109472	HSPD1 IL12<br>CPE
hsa04928	Parathyroid hormone synthesis, secretion and action	19/1188	84/5573	0.427173545646047	0.80861151516974	ENSG00000113448<br>ENSG00000170776<br>ENSG00000150995<br>ENSG00000128272<br>ENSG00000182158<br>ENSG00000149782<br>ENSG00000204103<br>ENSG00000132155<br>ENSG00000174233<br>ENSG00000125966<br>ENSG00000162337<br>ENSG00000065989<br>ENSG00000096433<br>ENSG00000102882<br>ENSG00000162104<br>ENSG00000170345<br>ENSG00000121281<br>ENSG00000157764<br>ENSG00000137486	PDE4D<br>AKAP13<br>ITPR1 ATF<br>CREB3L2<br>PLCB3 MAF<br>RAF1 ADCY<br>MMP24 LRF<br>PDE4A ITPF<br>MAPK3<br>ADCY9 FO<br>ADCY7 BRA<br>ARRB1
hsa00601	Glycosphingolipid biosynthesis - lacto and neolacto series	5/1188	20/5573	0.428121691392198	0.80861151516974	ENSG00000086062<br>ENSG00000117411<br>ENSG00000196371<br>ENSG00000158850<br>ENSG00000110080	B4GALT1<br>B4GALT2<br>FUT4<br>B4GALT3<br>ST3GAL4
hsa04066	HIF-1 signaling pathway	20/1188	89/5573	0.435896552553974	0.80861151516974	ENSG00000136244<br>ENSG00000152256<br>ENSG00000109320<br>ENSG00000102144<br>ENSG00000111640<br>ENSG00000027697<br>ENSG00000005339<br>ENSG00000099875<br>ENSG00000108515<br>ENSG00000135766<br>ENSG00000171105<br>ENSG00000102882<br>ENSG00000112715<br>ENSG00000100644<br>ENSG00000156510<br>ENSG00000197943<br>ENSG00000117394<br>ENSG00000121879<br>ENSG00000100387<br>ENSG00000187840	IL6 PDK1<br>NFKB1 PGK<br>GAPDH<br>IFNGR1<br>CREBBP<br>MKNK2 ENC<br>EGLN1 INS<br>MAPK3<br>VEGFA HIF1<br>HKDC1<br>PLCG2<br>SLC2A1<br>PIK3CA RBX<br>EIF4EBP1
hsa00600	Sphingolipid metabolism	10/1188	43/5573	0.436636515706143	0.80861151516974	ENSG00000176170<br>ENSG00000198964<br>ENSG00000143753<br>ENSG00000103056<br>ENSG00000067113<br>ENSG00000141934<br>ENSG00000164023<br>ENSG00000143418<br>ENSG00000148154<br>ENSG00000162139	SPHK1 SGM<br>DEGS1 SMPI<br>PLPP1 PLPF<br>SGMS2 CERS<br>UGCG NEU
hsa00562	Inositol phosphate metabolism	15/1188	66/5573	0.437229679212302	0.80861151516974	ENSG00000133056<br>ENSG00000149782<br>ENSG00000148384<br>ENSG00000103502<br>ENSG00000197943<br>ENSG00000143398<br>ENSG00000171862<br>ENSG00000121879<br>ENSG00000078269<br>ENSG00000086544<br>ENSG00000151151<br>ENSG00000108389<br>ENSG00000119711<br>ENSG00000241973<br>ENSG00000163719	PIK3C2B<br>PLCB3<br>INPP5E CDII<br>PLCG2<br>PIP5K1A<br>PTEN PIK3C<br>SYNJ2 ITPK<br>IPMK MTMF<br>ALDH6A1<br>PI4KA<br>MTMR14
hsa05410	Hypertrophic cardiomyopathy (HCM)	15/1188	66/5573	0.437229679212302	0.80861151516974	ENSG00000136244<br>ENSG00000105855<br>ENSG00000082781<br>ENSG00000184009<br>ENSG00000213949<br>ENSG00000155657<br>ENSG00000157445<br>ENSG00000173402	IL6 ITGB8<br>ITGB5 ACTC<br>ITGA1 TTN<br>CACNA2D<br>DAG1 DES<br>DMD RYR2<br>ITGA2 LMN<br>EMD ITGA

						ENSG00000175084 ENSG00000198947 ENSG00000198626 ENSG00000164171 ENSG00000160789 ENSG00000102119 ENSG00000005884	
hsa04015	Rap1 signaling pathway	37/1188	168/5573	0.441038786288409	0.810771900542166	ENSG00000108984 ENSG00000137801 ENSG00000169242 ENSG00000105976 ENSG00000149782 ENSG00000184009 ENSG00000197555 ENSG00000198561 ENSG00000105287 ENSG00000160867 ENSG00000132155 ENSG00000142627 ENSG00000158987 ENSG00000144118 ENSG00000174775 ENSG00000109756 ENSG00000068078 ENSG00000174233 ENSG00000050820 ENSG00000185386 ENSG00000171105 ENSG00000197122 ENSG00000102882 ENSG00000188130 ENSG00000173511 ENSG00000162104 ENSG00000151276 ENSG00000112715 ENSG00000128512 ENSG00000184371 ENSG00000079337 ENSG00000121879 ENSG00000121281 ENSG00000067606 ENSG00000157764 ENSG00000070087 ENSG00000125968	MAP2K6 THBS1 EFN[illegible] MET PLCB[illegible] ACTG1 SIPA1L1 CTNND1 PRKD2 FGF[illegible] RAF1 EPHA[illegible] RAPGEF6 RALB HRA[illegible] RAPGEF2 FGFR3 ADC[illegible] BCAR1 MAPK11 IN[illegible] SRC MAPK[illegible] MAPK12 VEGFB ADC[illegible] MAGI1 VEG[illegible] DOCK4 CSF[illegible] RAPGEF3 PIK3CA ADCY7 PRK[illegible] BRAF PFN[illegible] ID1
hsa00450	Selenocompound metabolism	4/1188	16/5573	0.454508804627462	0.828002275810507	ENSG00000247626 ENSG00000166986 ENSG00000116984 ENSG00000198431	MARS2 MA[illegible] MTR TXNR[illegible]
hsa04530	Tight junction	31/1188	141/5573	0.455805813068325	0.828002275810507	ENSG00000114019 ENSG00000163347 ENSG00000049759 ENSG00000171992 ENSG00000134318 ENSG00000110092 ENSG00000177606 ENSG00000127824 ENSG00000184009 ENSG00000159216 ENSG00000171132 ENSG00000158987 ENSG00000135446 ENSG00000133026 ENSG00000109756 ENSG00000060138 ENSG00000133627 ENSG00000123416 ENSG00000075711 ENSG00000197122 ENSG00000151276 ENSG00000132849 ENSG00000181885 ENSG00000105289 ENSG00000125755 ENSG00000167553 ENSG00000154721 ENSG00000147065 ENSG00000101608 ENSG00000067606 ENSG00000137710	AMOTL2 CLDN1 NEDD4L SYNPO ROCK2 CCND1 JU[illegible] TUBA4A ACTG1 RUNX1 PRK[illegible] RAPGEF6 CDK4 MYH[illegible] RAPGEF2 YBX3 ACTR[illegible] TUBA1B DL[illegible] SRC MAGI[illegible] PATJ CLDN[illegible] TJP3 SYMP[illegible] TUBA1C JA[illegible] MSN MYL12 PRKCZ RD[illegible]
hsa00280	Valine, leucine and isoleucine degradation	10/1188	44/5573	0.468269927826643	0.845640399075173	ENSG00000083123 ENSG00000106049	BCKDHB HIBADH

						ENSG00000164904 ENSG00000146085 ENSG00000128928 ENSG00000120437 ENSG00000196177 ENSG00000084754 ENSG00000117054 ENSG00000119711	ALDH7A1 MUT IVD ACAT2 ACADSB HADHA ACADM ALDH6A1
hsa00603	Glycosphingolipid biosynthesis - globo and isoglobo series	3/1188	12/5573	0.487964463480914	0.861463660685045	ENSG00000198951 ENSG00000008513 ENSG00000157350	NAGA ST3GAL1 ST3GAL2
hsa00010	Glycolysis / Gluconeogenesis	12/1188	54/5573	0.488846457933062	0.861463660685045	ENSG00000109107 ENSG00000102144 ENSG00000111640 ENSG00000006534 ENSG00000079739 ENSG00000171989 ENSG00000111716 ENSG00000108515 ENSG00000164904 ENSG00000156510 ENSG00000143891 ENSG00000108602	ALDOC PGK GAPDH ALDH3B1 PGM1 LDHAL6B LDHB ENO ALDH7A1 HKDC1 GAL ALDH3A1
hsa03050	Proteasome	9/1188	40/5573	0.489323703058082	0.861463660685045	ENSG00000092010 ENSG00000101182 ENSG00000143106 ENSG00000108344 ENSG00000100519 ENSG00000175166 ENSG00000013275 ENSG00000100902 ENSG00000159377	PSME1 PSM PSMA5 PSM PSMC6 PSM PSMC4 PSM PSMB4
hsa00052	Galactose metabolism	6/1188	26/5573	0.489490425191813	0.861463660685045	ENSG00000086062 ENSG00000085662 ENSG00000117411 ENSG00000079739 ENSG00000156510 ENSG00000143891	B4GALT1 AKR1B1 B4GALT2 PGM1 HKD GALM
hsa04260	Cardiac muscle contraction	13/1188	59/5573	0.497740615279073	0.861463660685045	ENSG00000143153 ENSG00000198804 ENSG00000198712 ENSG00000179091 ENSG00000131174 ENSG00000198938 ENSG00000184076 ENSG00000157445 ENSG00000127540 ENSG00000127184 ENSG00000198626 ENSG00000233954 ENSG00000140740	ATP1B1 MT CO1 MT-CC CYC1 COX7 MT-CO3 UQCR10 CACNA2D UQCR11 COX7C RYR UQCRHL UQCRC2
hsa04012	ErbB signaling pathway	17/1188	78/5573	0.50371557809381	0.861463660685045	ENSG00000082701 ENSG00000177606 ENSG00000132155 ENSG00000174775 ENSG00000143322 ENSG00000100485 ENSG00000124882 ENSG00000197122 ENSG00000102882 ENSG00000197943 ENSG00000163235 ENSG00000169752 ENSG00000121879 ENSG00000065559 ENSG00000126561 ENSG00000157764 ENSG00000187840	GSK3B JUN RAF1 HRA ABL2 SOS EREG SRC MAPK3 PLC TGFA NRG PIK3CA MAP2K4 STAT5A BR EIF4EBP1
hsa00220	Arginine biosynthesis	4/1188	17/5573	0.505821572997362	0.861463660685045	ENSG00000021826 ENSG00000081181 ENSG00000166123 ENSG00000130707	CPS1 ARG GPT2 ASS
hsa01210	2-Oxocarboxylic acid metabolism	4/1188	17/5573	0.505821572997362	0.861463660685045	ENSG00000182054 ENSG00000138413 ENSG00000166411 ENSG00000166123	IDH2 IDH1 IDH3A GPT
hsa04977	Vitamin digestion and absorption	4/1188	17/5573	0.505821572997362	0.861463660685045	ENSG00000185339 ENSG00000173638	TCN2 SLC19A1

						ENSG00000138074 ENSG00000117479	SLC5A6 SLC19A2
hsa04664	Fc epsilon RI signaling pathway	12/1188	55/5573	0.517081008394347	0.861463660685045	ENSG00000108984 ENSG00000134215 ENSG00000132155 ENSG00000174775 ENSG00000100485 ENSG00000185386 ENSG00000102882 ENSG00000188130 ENSG00000197943 ENSG00000121879 ENSG00000065559 ENSG00000243708	MAP2K6 VA[illegible] RAF1 HRA[illegible] SOS2 MAPK[illegible] MAPK3 MAPK12 PLCG2 PIK3CA MAP2K4 PLA2G4B
hsa04966	Collecting duct acid secretion	5/1188	22/5573	0.519064936810359	0.861463660685045	ENSG00000184908 ENSG00000110719 ENSG00000116039 ENSG00000105675 ENSG00000114573	CLCNKB TCIRG1 ATP6V1B1 ATP4A ATP6V1A
hsa05340	Primary immunodeficiency	5/1188	22/5573	0.519064936810359	0.861463660685045	ENSG00000276045 ENSG00000143390 ENSG00000105369 ENSG00000160224 ENSG00000147168	ORAI1 RFX[illegible] CD79A AIR[illegible] IL2RG
hsa01200	Carbon metabolism	23/1188	107/5573	0.520608704066263	0.861463660685045	ENSG00000173599 ENSG00000135069 ENSG00000109107 ENSG00000102144 ENSG00000182054 ENSG00000146733 ENSG00000021826 ENSG00000111640 ENSG00000138413 ENSG00000166411 ENSG00000166123 ENSG00000108515 ENSG00000146085 ENSG00000117118 ENSG00000184207 ENSG00000153574 ENSG00000120437 ENSG00000156510 ENSG00000084754 ENSG00000160211 ENSG00000121691 ENSG00000117054 ENSG00000119711	PC PSAT1 ALDOC PG[illegible] IDH2 PSP[illegible] CPS1 GAPD[illegible] IDH1 IDH3[illegible] GPT2 ENO[illegible] MUT SDH[illegible] PGP RPIA ACAT2 HKDC1 HADHA G6[illegible] CAT ACAD[illegible] ALDH6A1
hsa04923	Regulation of lipolysis in adipocytes	9/1188	41/5573	0.522005009205686	0.861463660685045	ENSG00000095303 ENSG00000073756 ENSG00000174233 ENSG00000043591 ENSG00000171105 ENSG00000162104 ENSG00000121879 ENSG00000121281 ENSG00000166819	PTGS1 PTG[illegible] ADCY6 ADR[illegible] INSR ADCY[illegible] PIK3CA ADCY7 PLI[illegible]
hsa00350	Tyrosine metabolism	6/1188	27/5573	0.529764272323754	0.861463660685045	ENSG00000158104 ENSG00000006534 ENSG00000131471 ENSG00000069535 ENSG00000180176 ENSG00000108602	HPD ALDH3[illegible] AOC3 MAO[illegible] TH ALDH3[illegible]
hsa00760	Nicotinate and nicotinamide metabolism	6/1188	27/5573	0.529764272323754	0.861463660685045	ENSG00000166741 ENSG00000135318 ENSG00000112992 ENSG00000076685 ENSG00000103485 ENSG00000198805	NNMT NT5[illegible] NNT NT5C[illegible] QPRT PN[illegible]
hsa01523	Antifolate resistance	6/1188	27/5573	0.529764272323754	0.861463660685045	ENSG00000136244 ENSG00000173638 ENSG00000109320 ENSG00000228716 ENSG00000110195 ENSG00000108846	IL6 SLC19A[illegible] NFKB1 DHF[illegible] FOLR1 ABC[illegible]
hsa04976	Bile secretion	10/1188	46/5573	0.530347335079718	0.861463660685045	ENSG00000130164 ENSG00000143153 ENSG00000143819 ENSG00000164889 ENSG00000174233	LDLR ATP1[illegible] EPHX1 SLC4A2 ADCY6 NCEH1

						ENSG00000144959 ENSG00000162104 ENSG00000108846 ENSG00000117394 ENSG00000121281	ADCY9 ABC[illegible] SLC2A1 ADCY7
hsa05110	Vibrio cholerae infection	10/1188	46/5573	0.530347335079718	0.861463660685045	ENSG00000117410 ENSG00000184009 ENSG00000155660 ENSG00000132432 ENSG00000162104 ENSG00000197943 ENSG00000110719 ENSG00000116039 ENSG00000143761 ENSG00000114573	ATP6V0B ACTG1 PDI[illegible] SEC61G ADCY9 PLC[illegible] TCIRG1 ATP6V1B1 ARF1 ATP6V1A
hsa04140	Autophagy - animal	26/1188	122/5573	0.536996904150428	0.865320142248214	ENSG00000176171 ENSG00000150995 ENSG00000168209 ENSG00000196730 ENSG00000078804 ENSG00000062716 ENSG00000164733 ENSG00000177169 ENSG00000139112 ENSG00000132155 ENSG00000174775 ENSG00000070540 ENSG00000175224 ENSG00000102882 ENSG00000123395 ENSG00000003402 ENSG00000100644 ENSG00000135047 ENSG00000189403 ENSG00000005893 ENSG00000171862 ENSG00000121879 ENSG00000110931 ENSG00000108389 ENSG00000130734 ENSG00000163719	BNIP3 ITPR[illegible] DDIT4 DAP[illegible] TP53INP2 VMP1 CTS[illegible] ULK1 GABARAPL[illegible] RAF1 HRA[illegible] WIPI1 ATG[illegible] MAPK3 ATG101 CFLAR HIF1[illegible] CTSL HMG[illegible] LAMP2 PTE[illegible] PIK3CA CAMKK2 MTMR4 ATG4D MTMR14
hsa04270	Vascular smooth muscle contraction	19/1188	89/5573	0.539446971885232	0.865320142248214	ENSG00000150995 ENSG00000122786 ENSG00000134318 ENSG00000149782 ENSG00000171132 ENSG00000132155 ENSG00000058272 ENSG00000174233 ENSG00000140795 ENSG00000096433 ENSG00000102882 ENSG00000162104 ENSG00000132329 ENSG00000196914 ENSG00000121281 ENSG00000135643 ENSG00000243708 ENSG00000157764 ENSG00000159899	ITPR1 CAL[illegible] ROCK2 PLC[illegible] PRKCE RAF[illegible] PPP1R12[illegible] ADCY6 MYL[illegible] ITPR3 MAP[illegible] ADCY9 RAMP1 ARHGEF1[illegible] ADCY7 KCNMB4 PLA2G4B BRAF NPR[illegible]
hsa04911	Insulin secretion	12/1188	56/5573	0.544852487667386	0.865320142248214	ENSG00000128272 ENSG00000143153 ENSG00000182158 ENSG00000149782 ENSG00000174233 ENSG00000104783 ENSG00000096433 ENSG00000162104 ENSG00000198626 ENSG00000117394 ENSG00000121281 ENSG00000135643	ATF4 ATP1[illegible] CREB3L2 PLCB3 ADC[illegible] KCNN4 ITP[illegible] ADCY9 RY[illegible] SLC2A1 ADCY7 KCNMB4
hsa04144	Endocytosis	46/1188	217/5573	0.545135271118504	0.865320142248214	ENSG00000153071 ENSG00000130164 ENSG00000166949 ENSG00000049759 ENSG00000110195 ENSG00000173020 ENSG00000108262 ENSG00000070371 ENSG00000175387	DAB2 LDL[illegible] SMAD3 NEDD4L FOLR1 GR[illegible] GIT1 CLTC[illegible] SMAD2 CHMP5 FGFR4 ASA[illegible] CAPZB HSP[illegible]

						ENSG00000086065<br>ENSG00000160867<br>ENSG00000088280<br>ENSG00000077549<br>ENSG00000173110<br>ENSG00000174775<br>ENSG00000197081<br>ENSG00000111540<br>ENSG00000068078<br>ENSG00000124356<br>ENSG00000197959<br>ENSG00000136950<br>ENSG00000134287<br>ENSG00000175416<br>ENSG00000197122<br>ENSG00000151502<br>ENSG00000107862<br>ENSG00000161203<br>ENSG00000100385<br>ENSG00000124209<br>ENSG00000008256<br>ENSG00000123124<br>ENSG00000130724<br>ENSG00000164961<br>ENSG00000143398<br>ENSG00000143761<br>ENSG00000078747<br>ENSG00000089006<br>ENSG00000153317<br>ENSG00000176783<br>ENSG00000067606<br>ENSG00000137486<br>ENSG00000165527<br>ENSG00000147168<br>ENSG00000198742<br>ENSG00000024422<br>ENSG00000157985	HRAS IGF2<br>RAB5B FGFI<br>STAMBP<br>DNM3<br>ARPC5L ARI<br>CLTB SRC<br>VPS26B GBI<br>AP2M1 IL2F<br>RAB22A<br>CYTH3 WWI<br>CHMP2A<br>WASHC5<br>PIP5K1A<br>ARF1 ITCH<br>SNX5 ASAP<br>RUFY1 PRK(<br>ARRB1 ARF<br>IL2RG<br>SMURF1 EHI<br>AGAP1
[hsa04930]()	Type II diabetes mellitus	8/1188	37/5573	0.546814682723627	0.865320142248214	ENSG00000171132<br>ENSG00000184557<br>ENSG00000171105<br>ENSG00000102882<br>ENSG00000120833<br>ENSG00000156510<br>ENSG00000121879<br>ENSG00000067606	PRKCE SOC<br>INSR MAPK<br>SOCS2 HKD<br>PIK3CA<br>PRKCZ
[hsa03060]()	Protein export	5/1188	23/5573	0.562178110013211	0.877235573417917	ENSG00000144867<br>ENSG00000167881<br>ENSG00000140319<br>ENSG00000132432<br>ENSG00000184903	SRPRB SRP<br>SRP14<br>SEC61G<br>IMMP2L
[hsa05020]()	Prion diseases	5/1188	23/5573	0.562178110013211	0.877235573417917	ENSG00000136244<br>ENSG00000148400<br>ENSG00000168439<br>ENSG00000102882<br>ENSG00000135862	IL6 NOTCH<br>STIP1 MAPK<br>LAMC1
[hsa03018]()	RNA degradation	15/1188	71/5573	0.562916638317035	0.877235573417917	ENSG00000090621<br>ENSG00000144381<br>ENSG00000183864<br>ENSG00000254535<br>ENSG00000159388<br>ENSG00000141232<br>ENSG00000130520<br>ENSG00000178896<br>ENSG00000108515<br>ENSG00000075914<br>ENSG00000077348<br>ENSG00000113300<br>ENSG00000111596<br>ENSG00000080802<br>ENSG00000151846	PABPC4<br>HSPD1 TOE<br>PABPC4L<br>BTG2 TOB<br>LSM4 EXOS<br>ENO3 EXOS<br>EXOSC5<br>CNOT6<br>CNOT2<br>CNOT4<br>PABPC3
[hsa04213]()	Longevity regulating pathway - multiple species	10/1188	48/5573	0.589766566271235	0.912857011232122	ENSG00000112096<br>ENSG00000173110<br>ENSG00000174775<br>ENSG00000174233<br>ENSG00000171105<br>ENSG00000162104<br>ENSG00000109846<br>ENSG00000121879<br>ENSG00000121281<br>ENSG00000121691	SOD2 HSPA<br>HRAS ADCY<br>INSR ADCY<br>CRYAB<br>PIK3CA<br>ADCY7 CAT

hsa04921	Oxytocin signaling pathway	26/1188	125/5573	0.592492943914967	0.912857011232122	ENSG00000150995<br>ENSG00000134318<br>ENSG00000110092<br>ENSG00000149782<br>ENSG00000177606<br>ENSG00000184009<br>ENSG00000073756<br>ENSG00000132155<br>ENSG00000058272<br>ENSG00000174775<br>ENSG00000174233<br>ENSG00000142185<br>ENSG00000157445<br>ENSG00000197122<br>ENSG00000140795<br>ENSG00000096433<br>ENSG00000102882<br>ENSG00000162104<br>ENSG00000170345<br>ENSG00000103319<br>ENSG00000198626<br>ENSG00000121281<br>ENSG00000110931<br>ENSG00000243708<br>ENSG00000198838<br>ENSG00000159899	ITPR1 ROCK<br>CCND1 PLC<br>JUN ACTG<br>PTGS2 RAF<br>PPP1R12A<br>HRAS ADCY<br>TRPM2<br>CACNA2D<br>SRC MYLK<br>ITPR3 MAPK<br>ADCY9 FO<br>EEF2K RYR<br>ADCY7<br>CAMKK2<br>PLA2G4B<br>RYR3 NPR
hsa05150	Staphylococcus aureus infection	4/1188	19/5573	0.601147707448998	0.912857011232122	ENSG00000125730<br>ENSG00000090339<br>ENSG00000182326<br>ENSG00000159403	C3 ICAM1 C<br>C1R
hsa04975	Fat digestion and absorption	5/1188	24/5573	0.603243356382749	0.912857011232122	ENSG00000167114<br>ENSG00000067113<br>ENSG00000141934<br>ENSG00000120437<br>ENSG00000169692	SLC27A4<br>PLPP1 PLPP<br>ACAT2<br>AGPAT2
hsa04670	Leukocyte transendothelial migration	19/1188	92/5573	0.60372558482152	0.912857011232122	ENSG00000134215<br>ENSG00000163347<br>ENSG00000134318<br>ENSG00000184009<br>ENSG00000198561<br>ENSG00000090339<br>ENSG00000050820<br>ENSG00000185386<br>ENSG00000188130<br>ENSG00000035403<br>ENSG00000181885<br>ENSG00000120899<br>ENSG00000116701<br>ENSG00000197943<br>ENSG00000154721<br>ENSG00000079337<br>ENSG00000147065<br>ENSG00000121879<br>ENSG00000101608	VAV3 CLDN<br>ROCK2<br>ACTG1<br>CTNND1<br>ICAM1 BCAR<br>MAPK11<br>MAPK12 VC<br>CLDN7 PTK<br>NCF2 PLCG<br>JAM2<br>RAPGEF3 M<br>PIK3CA<br>MYL12A
hsa04392	Hippo signaling pathway - multiple species	6/1188	29/5573	0.60591192035848	0.912857011232122	ENSG00000187079<br>ENSG00000150457<br>ENSG00000068028<br>ENSG00000129474<br>ENSG00000018408<br>ENSG00000139926	TEAD1 LATS<br>RASSF1<br>AJUBA<br>WWTR1<br>FRMD6
hsa05100	Bacterial invasion of epithelial cells	14/1188	68/5573	0.606589023750335	0.912857011232122	ENSG00000105976<br>ENSG00000184009<br>ENSG00000070371<br>ENSG00000115414<br>ENSG00000197959<br>ENSG00000050820<br>ENSG00000136950<br>ENSG00000125354<br>ENSG00000175416<br>ENSG00000197122<br>ENSG00000035403<br>ENSG00000116670<br>ENSG00000121879<br>ENSG00000102890	MET ACTG<br>CLTCL1 FN<br>DNM3 BCAR<br>ARPC5L<br>SEPT6 CLT<br>SRC VCL<br>MAD2L2<br>PIK3CA<br>ELMO3
hsa04070	Phosphatidylinositol signaling system	18/1188	88/5573	0.620975648816695	0.929948898471831	ENSG00000150995<br>ENSG00000133056<br>ENSG00000149091<br>ENSG00000149782<br>ENSG00000148384<br>ENSG00000101290	ITPR1<br>PIK3C2B<br>DGKZ PLCB<br>INPP5E CDS<br>ITPR3 CDIP<br>PLCG2

						ENSG00000096433 ENSG00000103502 ENSG00000197943 ENSG00000143398 ENSG00000171862 ENSG00000121879 ENSG00000078269 ENSG00000086544 ENSG00000151151 ENSG00000108389 ENSG00000241973 ENSG00000163719	PIP5K1A PTEN PIK3C SYNJ2 ITPK IPMK MTMR PI4KA MTMR14
hsa03440	Homologous recombination	8/1188	40/5573	0.642565793663757	0.955710593812568	ENSG00000136492 ENSG00000158019 ENSG00000172732 ENSG00000149311 ENSG00000106628 ENSG00000087206 ENSG00000085999 ENSG00000062822	BRIP1 BABAM2 MUS81 ATI POLD2 UIMC RAD54L POLD1
hsa04672	Intestinal immune network for IgA production	4/1188	20/5573	0.644404211463198	0.955710593812568	ENSG00000136244 ENSG00000151882 ENSG00000164136 ENSG00000184451	IL6 CCL28 IL15 CCR1
hsa04217	Necroptosis	27/1188	133/5573	0.648010166908013	0.956438082888269	ENSG00000162909 ENSG00000118503 ENSG00000023445 ENSG00000080824 ENSG00000083799 ENSG00000162434 ENSG00000110330 ENSG00000164032 ENSG00000142166 ENSG00000168404 ENSG00000027697 ENSG00000086065 ENSG00000101966 ENSG00000168040 ENSG00000096384 ENSG00000180573 ENSG00000003402 ENSG00000115415 ENSG00000189403 ENSG00000171497 ENSG00000130724 ENSG00000247077 ENSG00000041880 ENSG00000243708 ENSG00000126561 ENSG00000143258 ENSG00000064012	CAPN2 TNFAIP3 BIRC3 HSP90AA1 CYLD JAK BIRC2 H2AF IFNAR1 MLK IFNGR1 CHMP5 XIA FADD HSP90AB1 HIST1H2A CFLAR STA HMGB1 PPI CHMP2A PGAM5 PARP3 PLA2G4B STAT5A USP21 CASI
hsa03015	mRNA surveillance pathway	16/1188	80/5573	0.657465970413876	0.960653364629427	ENSG00000165494 ENSG00000176102 ENSG00000090621 ENSG00000100941 ENSG00000071626 ENSG00000254535 ENSG00000265241 ENSG00000153944 ENSG00000162231 ENSG00000198952 ENSG00000172409 ENSG00000114503 ENSG00000141543 ENSG00000125755 ENSG00000005007 ENSG00000151846	PCF11 CSTI PABPC4 PN DAZAP1 PABPC4L RBM8A MSI NXF1 SMG CLP1 NCBP EIF4A3 SYMPK UPF PABPC3
hsa01212	Fatty acid metabolism	9/1188	46/5573	0.671515285920597	0.960653364629427	ENSG00000100294 ENSG00000110090 ENSG00000099194 ENSG00000120437 ENSG00000196177 ENSG00000012660 ENSG00000115425 ENSG00000084754 ENSG00000117054	MCAT CPT1 SCD ACAT ACADSB ELOVL5 PEC HADHA ACADM
hsa00970	Aminoacyl-tRNA biosynthesis	8/1188	41/5573	0.671792650502076	0.960653364629427	ENSG00000247626 ENSG00000179115 ENSG00000130348 ENSG00000134684	MARS2 FAR QRSL1 YAR KARS MAR PARS2 TAR

						ENSG00000065427<br>ENSG00000166986<br>ENSG00000162396<br>ENSG00000113407	
hsa05014	Amyotrophic lateral sclerosis (ALS)	8/1188	41/5573	0.671792650502076	0.960653364629427	ENSG00000108984<br>ENSG00000100285<br>ENSG00000130204<br>ENSG00000185386<br>ENSG00000188130<br>ENSG00000233276<br>ENSG00000121691<br>ENSG00000141510	MAP2K6<br>NEFH<br>TOMM40<br>MAPK11<br>MAPK12 GP<br>CAT TP53
hsa00310	Lysine degradation	11/1188	56/5573	0.672316577639226	0.960653364629427	ENSG00000152952<br>ENSG00000130309<br>ENSG00000272333<br>ENSG00000106397<br>ENSG00000116539<br>ENSG00000164904<br>ENSG00000120437<br>ENSG00000084754<br>ENSG00000104885<br>ENSG00000108799<br>ENSG00000106462	PLOD2<br>COLGALT<br>KMT2B<br>PLOD3 ASH<br>ALDH7A1<br>ACAT2<br>HADHA<br>DOT1L EZH<br>EZH2
hsa00260	Glycine, serine and threonine metabolism	7/1188	36/5573	0.672770271646016	0.960653364629427	ENSG00000135069<br>ENSG00000146733<br>ENSG00000131471<br>ENSG00000164904<br>ENSG00000069535<br>ENSG00000016391<br>ENSG00000123453	PSAT1 PSP<br>AOC3<br>ALDH7A1<br>MAOB CHD<br>SARDH
hsa04978	Mineral absorption	7/1188	36/5573	0.672770271646016	0.960653364629427	ENSG00000071967<br>ENSG00000143153<br>ENSG00000170385<br>ENSG00000110911<br>ENSG00000070961<br>ENSG00000103415<br>ENSG00000165240	CYBRD1<br>ATP1B1<br>SLC30A1<br>SLC11A2<br>ATP2B1<br>HMOX2<br>ATP7A
hsa04973	Carbohydrate digestion and absorption	5/1188	26/5573	0.678331532580551	0.964110094917728	ENSG00000143153<br>ENSG00000156510<br>ENSG00000169962<br>ENSG00000121879<br>ENSG00000142583	ATP1B1<br>HKDC1<br>TAS1R3<br>PIK3CA<br>SLC2A5
hsa04022	cGMP-PKG signaling pathway	24/1188	121/5573	0.691176476962942	0.977839531924531	ENSG00000172572<br>ENSG00000150995<br>ENSG00000128272<br>ENSG00000143153<br>ENSG00000134318<br>ENSG00000182158<br>ENSG00000149782<br>ENSG00000171132<br>ENSG00000132155<br>ENSG00000058272<br>ENSG00000174233<br>ENSG00000043591<br>ENSG00000171105<br>ENSG00000058668<br>ENSG00000070961<br>ENSG00000140795<br>ENSG00000096433<br>ENSG00000102882<br>ENSG00000162104<br>ENSG00000070729<br>ENSG00000121281<br>ENSG00000135643<br>ENSG00000159899<br>ENSG00000263001	PDE3A ITP<br>ATF4 ATP1<br>ROCK2<br>CREB3L2<br>PLCB3 PRK<br>RAF1<br>PPP1R12A<br>ADCY6 ADR<br>INSR ATP2<br>ATP2B1<br>MYLK3 ITP<br>MAPK3<br>ADCY9<br>CNGB1<br>ADCY7<br>KCNMB4<br>NPR2 GTF
hsa04972	Pancreatic secretion	11/1188	57/5573	0.69629438400554	0.979197979679505	ENSG00000150995<br>ENSG00000143153<br>ENSG00000149782<br>ENSG00000164889<br>ENSG00000174233<br>ENSG00000058668<br>ENSG00000070961<br>ENSG00000096433<br>ENSG00000162104<br>ENSG00000198626<br>ENSG00000121281	ITPR1<br>ATP1B1<br>PLCB3<br>SLC4A2<br>ADCY6<br>ATP2B4<br>ATP2B1<br>ITPR3 ADC<br>RYR2 ADC
hsa04922	Glucagon signaling pathway	17/1188	87/5573	0.699088045694299	0.979197979679505	ENSG00000150995<br>ENSG00000128272<br>ENSG00000182158	ITPR1 ATF<br>CREB3L2<br>PLCB3

						ENSG00000149782<br>ENSG00000005339<br>ENSG00000170145<br>ENSG00000110090<br>ENSG00000171989<br>ENSG00000275993<br>ENSG00000126457<br>ENSG00000111716<br>ENSG00000096433<br>ENSG00000142178<br>ENSG00000076555<br>ENSG00000117394<br>ENSG00000149923<br>ENSG00000067177	CREBBP SIK<br>CPT1A<br>LDHAL6B<br>SIK1B PRMT<br>LDHB ITPR<br>SIK1 ACAC<br>SLC2A1<br>PPP4C PHK
hsa00030	Pentose phosphate pathway	5/1188	27/5573	0.712096819002551	0.979197979679505	ENSG00000109107<br>ENSG00000079739<br>ENSG00000153574<br>ENSG00000160211<br>ENSG00000171174	ALDOC PGM<br>RPIA G6PD<br>RBKS
hsa00061	Fatty acid biosynthesis	2/1188	11/5573	0.715444901328267	0.979197979679505	ENSG00000100294<br>ENSG00000076555	MCAT ACAC
hsa00130	Ubiquinone and other terpenoid-quinone biosynthesis	2/1188	11/5573	0.715444901328267	0.979197979679505	ENSG00000158104<br>ENSG00000167397	HPD VKORC
hsa05231	Choline metabolism in cancer	17/1188	88/5573	0.717791013960768	0.979197979679505	ENSG00000110721<br>ENSG00000149091<br>ENSG00000177606<br>ENSG00000125772<br>ENSG00000132155<br>ENSG00000174775<br>ENSG00000100485<br>ENSG00000067113<br>ENSG00000141934<br>ENSG00000102882<br>ENSG00000111666<br>ENSG00000170345<br>ENSG00000100644<br>ENSG00000143398<br>ENSG00000121879<br>ENSG00000243708<br>ENSG00000187840	CHKA DGK<br>JUN GPCPD<br>RAF1 HRAS<br>SOS2 PLPP<br>PLPP2 MAPI<br>CHPT1 FO<br>HIF1A<br>PIP5K1A<br>PIK3CA<br>PLA2G4B<br>EIF4EBP1
hsa00520	Amino sugar and nucleotide sugar metabolism	8/1188	43/5573	0.725692486377153	0.979197979679505	ENSG00000113552<br>ENSG00000079739<br>ENSG00000159921<br>ENSG00000109814<br>ENSG00000197355<br>ENSG00000156510<br>ENSG00000215883<br>ENSG00000198380	GNPDA1<br>PGM1 GNE<br>UGDH<br>UAP1L1<br>HKDC1<br>CYB5RL<br>GFPT1
hsa03022	Basal transcription factors	7/1188	38/5573	0.729754650955365	0.979197979679505	ENSG00000165632<br>ENSG00000166337<br>ENSG00000104884<br>ENSG00000120656<br>ENSG00000134480<br>ENSG00000183474<br>ENSG00000263001	TAF3 TAF1<br>ERCC2 TAF<br>CCNH<br>GTF2H2C<br>GTF2I
hsa04962	Vasopressin-regulated water reabsorption	7/1188	38/5573	0.729754650955365	0.979197979679505	ENSG00000088986<br>ENSG00000182158<br>ENSG00000165272<br>ENSG00000111540<br>ENSG00000174233<br>ENSG00000141522<br>ENSG00000162104	DYNLL1<br>CREB3L2<br>AQP3 RAB5<br>ADCY6<br>ARHGDIA<br>ADCY9
hsa00620	Pyruvate metabolism	6/1188	33/5573	0.735380697237882	0.979197979679505	ENSG00000173599<br>ENSG00000171989<br>ENSG00000111716<br>ENSG00000164904<br>ENSG00000120437<br>ENSG00000076555	PC LDHAL6<br>LDHB<br>ALDH7A1<br>ACAT2 ACA
hsa00531	Glycosaminoglycan degradation	3/1188	17/5573	0.735705249689614	0.979197979679505	ENSG00000165102<br>ENSG00000068001<br>ENSG00000186792	HGSNAT<br>HYAL2 HYA
hsa00020	Citrate cycle (TCA cycle)	5/1188	28/5573	0.74327937521853	0.979197979679505	ENSG00000173599<br>ENSG00000182054<br>ENSG00000138413<br>ENSG00000166411<br>ENSG00000117118	PC IDH2 IDH<br>IDH3A SDH
hsa00561	Glycerolipid metabolism	9/1188	49/5573	0.746289250380816	0.979197979679505	ENSG00000085662<br>ENSG00000149091	AKR1B1 DGI<br>MBOAT2

						ENSG00000143797<br>ENSG00000164904<br>ENSG00000067113<br>ENSG00000141934<br>ENSG00000169692<br>ENSG00000101577<br>ENSG00000100344	ALDH7A1<br>PLPP1 PLPI<br>AGPAT2<br>LPIN2<br>PNPLA3
hsa05030	Cocaine addiction	7/1188	39/5573	0.755531554951902	0.979197979679505	ENSG00000128272<br>ENSG00000109320<br>ENSG00000176749<br>ENSG00000182158<br>ENSG00000177606<br>ENSG00000069535<br>ENSG00000180176	ATF4 NFKB<br>CDK5R1<br>CREB3L2 JL<br>MAOB TH
hsa04024	cAMP signaling pathway	29/1188	150/5573	0.755823514863949	0.979197979679505	ENSG00000113448<br>ENSG00000172572<br>ENSG00000134215<br>ENSG00000143153<br>ENSG00000109320<br>ENSG00000134318<br>ENSG00000182158<br>ENSG00000177606<br>ENSG00000100906<br>ENSG00000005339<br>ENSG00000132155<br>ENSG00000276045<br>ENSG00000058272<br>ENSG00000139874<br>ENSG00000174233<br>ENSG00000043591<br>ENSG00000065989<br>ENSG00000058668<br>ENSG00000070961<br>ENSG00000102882<br>ENSG00000162104<br>ENSG00000070729<br>ENSG00000170345<br>ENSG00000099822<br>ENSG00000198626<br>ENSG00000079337<br>ENSG00000121879<br>ENSG00000121281<br>ENSG00000157764	PDE4D PDE<br>VAV3 ATP1I<br>NFKB1 ROC<br>CREB3L2 JL<br>NFKBIA<br>CREBBP RAI<br>ORAI1<br>PPP1R12A<br>SSTR1 ADC<br>ADRB1 PDE<br>ATP2B4<br>ATP2B1<br>MAPK3<br>ADCY9<br>CNGB1 FO<br>HCN2 RYR<br>RAPGEF3<br>PIK3CA<br>ADCY7 BRA
hsa04060	Cytokine-cytokine receptor interaction	32/1188	165/5573	0.758020795986072	0.979197979679505	ENSG00000136244<br>ENSG00000006327<br>ENSG00000169429<br>ENSG00000115009<br>ENSG00000164761<br>ENSG00000130513<br>ENSG00000139269<br>ENSG00000174564<br>ENSG00000142166<br>ENSG00000168811<br>ENSG00000027697<br>ENSG00000095752<br>ENSG00000104998<br>ENSG00000163739<br>ENSG00000125845<br>ENSG00000116985<br>ENSG00000177663<br>ENSG00000151882<br>ENSG00000049249<br>ENSG00000173530<br>ENSG00000128342<br>ENSG00000008517<br>ENSG00000100385<br>ENSG00000213218<br>ENSG00000184371<br>ENSG00000107779<br>ENSG00000164136<br>ENSG00000242689<br>ENSG00000184451<br>ENSG00000147168<br>ENSG00000169248<br>ENSG00000135503	IL6<br>TNFRSF12<br>CXCL8 CCL<br>TNFRSF11<br>GDF15 INH<br>IL20RB<br>IFNAR1 IL1<br>IFNGR1 IL1<br>IL27RA CXC<br>BMP2 BMP<br>IL17RA CCL<br>TNFRSF9<br>TNFRSF10<br>LIF IL32<br>IL2RB CSH<br>CSF1 BMPR<br>IL15 CNTF<br>CCR10 IL2R<br>CXCL11<br>ACVR1B
hsa00910	Nitrogen metabolism	2/1188	12/5573	0.760940988897737	0.979197979679505	ENSG00000107159<br>ENSG00000021826	CA9 CPS1
hsa02010	ABC transporters	6/1188	34/5573	0.762493923698735	0.979197979679505	ENSG00000154265<br>ENSG00000160179<br>ENSG00000064687<br>ENSG00000135776	ABCA5 ABC<br>ABCA7<br>ABCB10<br>ABCA4 ABC

						ENSG00000198691 ENSG00000108846	
hsa04742	Taste transduction	6/1188	34/5573	0.762493923698735	0.979197979679505	ENSG00000174233 ENSG00000111319 ENSG00000096433 ENSG00000169962 ENSG00000115252 ENSG00000164626	ADCY6 SCNN1A ITPR3 TAS1 PDE1A KCNI
hsa04971	Gastric acid secretion	10/1188	55/5573	0.764560326960954	0.979197979679505	ENSG00000150995 ENSG00000143153 ENSG00000149782 ENSG00000164889 ENSG00000174233 ENSG00000140795 ENSG00000096433 ENSG00000162104 ENSG00000105675 ENSG00000121281	ITPR1 ATP1B1 PLCB3 SLC4A2 ADCY6 ITPR3 ADCY ATP4A ADC
hsa04371	Apelin signaling pathway	22/1188	116/5573	0.767222038492732	0.979197979679505	ENSG00000176170 ENSG00000150995 ENSG00000118523 ENSG00000166949 ENSG00000110092 ENSG00000149782 ENSG00000175387 ENSG00000171132 ENSG00000139112 ENSG00000132155 ENSG00000174775 ENSG00000174233 ENSG00000140795 ENSG00000096433 ENSG00000172354 ENSG00000102882 ENSG00000162104 ENSG00000198626 ENSG00000121281 ENSG00000198838 ENSG00000166819 ENSG00000108064	SPHK1 ITPF CTGF SMAD CCND1 PLCI SMAD2 PRK GABARAPL RAF1 HRAS ADCY6 MYL ITPR3 GNB MAPK3 ADCY9 RYR ADCY7 RYR PLIN1 TFA
hsa04721	Synaptic vesicle cycle	9/1188	50/5573	0.768414802898943	0.979197979679505	ENSG00000117410 ENSG00000168993 ENSG00000070371 ENSG00000197959 ENSG00000175416 ENSG00000161203 ENSG00000110719 ENSG00000116039 ENSG00000114573	ATP6V0B CPLX1 CLTCL1 DN CLTB AP2M TCIRG1 ATP6V1B1 ATP6V1A
hsa04924	Renin secretion	9/1188	50/5573	0.768414802898943	0.979197979679505	ENSG00000172572 ENSG00000150995 ENSG00000149782 ENSG00000164733 ENSG00000276045 ENSG00000174233 ENSG00000043591 ENSG00000096433 ENSG00000115252	PDE3A ITPF PLCB3 CTS ORAI1 ADC ADRB1 ITPF PDE1A
hsa04726	Serotonergic synapse	15/1188	81/5573	0.771841011915381	0.979197979679505	ENSG00000150995 ENSG00000095303 ENSG00000149782 ENSG00000073756 ENSG00000120129 ENSG00000132155 ENSG00000174775 ENSG00000142192 ENSG00000096433 ENSG00000172354 ENSG00000102882 ENSG00000069535 ENSG00000079337 ENSG00000243708 ENSG00000157764	ITPR1 PTGS PLCB3 PTG DUSP1 RAF HRAS APP ITPR3 GNB MAPK3 MAC RAPGEF3 PLA2G4B BRAF
hsa00534	Glycosaminoglycan biosynthesis - heparan sulfate / heparin	3/1188	18/5573	0.771875931864626	0.979197979679505	ENSG00000182197 ENSG00000015532 ENSG00000012232	EXT1 XYLT EXTL3
hsa04360	Axon guidance	29/1188	152/5573	0.78124783453464	0.979600898257564	ENSG00000152256 ENSG00000082701 ENSG00000169242 ENSG00000075223	PDK1 GSK3 EFNA1 SEMA3C WNT5A

						ENSG00000114251<br>ENSG00000092964<br>ENSG00000074527<br>ENSG00000134318<br>ENSG00000105976<br>ENSG00000196411<br>ENSG00000114554<br>ENSG00000169855<br>ENSG00000132155<br>ENSG00000142627<br>ENSG00000174775<br>ENSG00000197122<br>ENSG00000102882<br>ENSG00000084112<br>ENSG00000187764<br>ENSG00000172757<br>ENSG00000001617<br>ENSG00000196935<br>ENSG00000197943<br>ENSG00000168758<br>ENSG00000196914<br>ENSG00000198910<br>ENSG00000106683<br>ENSG00000121879<br>ENSG00000067606	DPYSL2 NT<br>ROCK2 ME<br>EPHB4<br>PLXNA1<br>ROBO1 RAF<br>EPHA2 HRA<br>SRC MAPK<br>SSH1 SEMA<br>CFL1 SEMA<br>SRGAP1<br>PLCG2<br>SEMA4C<br>ARHGEF1<br>L1CAM LIM<br>PIK3CA<br>PRKCZ
hsa04142	Lysosome	22/1188	117/5573	0.781378865553436	0.979600898257564	ENSG00000198951<br>ENSG00000117410<br>ENSG00000070371<br>ENSG00000174080<br>ENSG00000164733<br>ENSG00000197746<br>ENSG00000165102<br>ENSG00000197081<br>ENSG00000110911<br>ENSG00000196743<br>ENSG00000134243<br>ENSG00000175416<br>ENSG00000144455<br>ENSG00000141337<br>ENSG00000132842<br>ENSG00000138760<br>ENSG00000078081<br>ENSG00000110719<br>ENSG00000135047<br>ENSG00000005893<br>ENSG00000081014<br>ENSG00000143387	NAGA<br>ATP6V0B<br>CLTCL1 CT<br>CTSB PSA<br>HGSNAT<br>IGF2R<br>SLC11A2<br>GM2A SOR<br>CLTB SUM<br>ARSG AP3<br>SCARB2<br>LAMP3<br>TCIRG1 CT<br>LAMP2 AP4<br>CTSK
hsa04720	Long-term potentiation	10/1188	56/5573	0.784613315943938	0.979600898257564	ENSG00000150995<br>ENSG00000128272<br>ENSG00000149782<br>ENSG00000005339<br>ENSG00000132155<br>ENSG00000174775<br>ENSG00000096433<br>ENSG00000102882<br>ENSG00000079337<br>ENSG00000157764	ITPR1 ATF<br>PLCB3<br>CREBBP RA<br>HRAS ITPR<br>MAPK3<br>RAPGEF3<br>BRAF
hsa00140	Steroid hormone biosynthesis	4/1188	24/5573	0.784957071567951	0.979600898257564	ENSG00000196139<br>ENSG00000101846<br>ENSG00000187134<br>ENSG00000140459	AKR1C3 ST<br>AKR1C1<br>CYP11A1
hsa00604	Glycosphingolipid biosynthesis - ganglio series	2/1188	13/5573	0.799987998058895	0.990307723403551	ENSG00000008513<br>ENSG00000157350	ST3GAL1<br>ST3GAL2
hsa04950	Maturity onset diabetes of the young	2/1188	13/5573	0.799987998058895	0.990307723403551	ENSG00000152804<br>ENSG00000130675	HHEX MNX
hsa00564	Glycerophospholipid metabolism	15/1188	83/5573	0.803966430782176	0.991235719880032	ENSG00000110721<br>ENSG00000149091<br>ENSG00000143797<br>ENSG00000125772<br>ENSG00000164089<br>ENSG00000067113<br>ENSG00000141934<br>ENSG00000101290<br>ENSG00000111666<br>ENSG00000103502<br>ENSG00000011009<br>ENSG00000169692<br>ENSG00000243708<br>ENSG00000116906<br>ENSG00000101577	CHKA DGK<br>MBOAT2<br>GPCPD1<br>ETNPPL<br>PLPP1 PLP<br>CDS2 CHPT<br>CDIPT<br>LYPLA2<br>AGPAT2<br>PLA2G4B<br>GNPAT LPI

hsa04910	Insulin signaling pathway	22/1188	119/5573	0.807921057305251	0.992127058370848	ENSG00000082701 ENSG00000142949 ENSG00000188191 ENSG00000132155 ENSG00000174775 ENSG00000099875 ENSG00000100485 ENSG00000184557 ENSG00000072310 ENSG00000171105 ENSG00000102882 ENSG00000120833 ENSG00000156510 ENSG00000076555 ENSG00000125733 ENSG00000121879 ENSG00000119938 ENSG00000160999 ENSG00000067606 ENSG00000157764 ENSG00000067177 ENSG00000187840	GSK3B PTPI PRKAR1B RAF1 HRA MKNK2 SOS SOCS3 SREBF1 INS MAPK3 SOC HKDC1 ACACB TRIP10 PIK3CA PPP1R3C SH2B2 PRK BRAF PHKA EIF4EBP1
hsa03040	Spliceosome	23/1188	125/5573	0.819336164021668	0.997564998291051	ENSG00000115875 ENSG00000275895 ENSG00000265241 ENSG00000051596 ENSG00000171960 ENSG00000173110 ENSG00000085872 ENSG00000077312 ENSG00000130520 ENSG00000114503 ENSG00000076924 ENSG00000143977 ENSG00000141543 ENSG00000169564 ENSG00000104852 ENSG00000179950 ENSG00000164610 ENSG00000099783 ENSG00000136527 ENSG00000124562 ENSG00000119707 ENSG00000125835 ENSG00000174243	SRSF7 U2AF1L5 RBM8A THOC3 PPI HSPA6 CHE SNRPA LSM NCBP2 XAB SNRPG EIF4A3 PCB SNRNP70 PUF60 RP HNRNPM TRA2B SNR RBM25 SNR DDX23
hsa00051	Fructose and mannose metabolism	5/1188	31/5573	0.821711251047486	0.997564998291051	ENSG00000085662 ENSG00000109107 ENSG00000156510 ENSG00000140263 ENSG00000138030	AKR1B1 ALDOC HKDC1 SOR KHK
hsa04340	Hedgehog signaling pathway	7/1188	42/5573	0.82209753930826	0.997564998291051	ENSG00000082701 ENSG00000110092 ENSG00000173020 ENSG00000133275 ENSG00000064309 ENSG00000137486 ENSG00000198742	GSK3B CCN GRK2 CSNK1G2 CDON ARR SMURF1
hsa00071	Fatty acid degradation	6/1188	37/5573	0.831402287340321	0.999998654289568	ENSG00000110090 ENSG00000164904 ENSG00000120437 ENSG00000196177 ENSG00000084754 ENSG00000117054	CPT1A ALDH7A1 ACAT2 ACADSB HADHA ACADM
hsa00500	Starch and sucrose metabolism	4/1188	26/5573	0.836520758748482	0.999998654289568	ENSG00000114480 ENSG00000163754 ENSG00000079739 ENSG00000156510	GBE1 GYG PGM1 HKD
hsa00630	Glyoxylate and dicarboxylate metabolism	4/1188	26/5573	0.836520758748482	0.999998654289568	ENSG00000146085 ENSG00000184207 ENSG00000120437 ENSG00000121691	MUT PGP ACAT2 CA
hsa04136	Autophagy - other	5/1188	32/5573	0.843114084071755	0.999998654289568	ENSG00000139112 ENSG00000070540 ENSG00000175224 ENSG00000123395 ENSG00000130734	GABARAPL WIPI1 ATG ATG101 ATG4D
hsa05170	Human immunodeficiency virus 1 infection	32/1188	173/5573	0.845012600377722	0.999998654289568	ENSG00000150995 ENSG00000108984 ENSG00000244509 ENSG00000109320	ITPR1 MAP2K6 APOBEC3 NFKB1 JU

						ENSG00000177606 ENSG00000179218 ENSG00000100906 ENSG00000167004 ENSG00000132155 ENSG00000184216 ENSG00000114125 ENSG00000174775 ENSG00000168040 ENSG00000030110 ENSG00000185386 ENSG00000096433 ENSG00000157456 ENSG00000172354 ENSG00000102882 ENSG00000188130 ENSG00000170345 ENSG00000172757 ENSG00000120899 ENSG00000197943 ENSG00000149311 ENSG00000106683 ENSG00000121879 ENSG00000100387 ENSG00000139842 ENSG00000100324 ENSG00000184584 ENSG00000064012	CALR NFKB PDIA3 RAF IRAK1 RNF HRAS FADI BAK1 MAPK ITPR3 CCNE GNB2 MAPK MAPK12 FC CFL1 PTK2 PLCG2 ATI LIMK1 PIK3(RBX1 CUL4 TAB1 TMEM173 CASP8
[hsa04730]()	Long-term depression	8/1188	49/5573	0.849866836914825	0.999998654289568	ENSG00000150995 ENSG00000149782 ENSG00000132155 ENSG00000174775 ENSG00000096433 ENSG00000102882 ENSG00000243708 ENSG00000157764	ITPR1 PLCE RAF1 HRA: ITPR3 MAPI PLA2G4B BRAF
[hsa04146]()	Peroxisome	13/1188	76/5573	0.852530302305315	0.999998654289568	ENSG00000124587 ENSG00000112096 ENSG00000182054 ENSG00000138413 ENSG00000254858 ENSG00000095321 ENSG00000197448 ENSG00000115425 ENSG00000114757 ENSG00000213965 ENSG00000121691 ENSG00000116906 ENSG00000139197	PEX6 SOD: IDH2 IDH1 MPV17L2 CRAT GSTI PECR PEX5 NUDT19 CA GNPAT PEX
[hsa00900]()	Terpenoid backbone biosynthesis	3/1188	21/5573	0.856629584421715	0.999998654289568	ENSG00000116237 ENSG00000120437 ENSG00000167508	ICMT ACAT MVD
[hsa04713]()	Circadian entrainment	13/1188	77/5573	0.865157289199486	0.999998654289568	ENSG00000150995 ENSG00000149782 ENSG00000174233 ENSG00000100784 ENSG00000096433 ENSG00000172354 ENSG00000102882 ENSG00000162104 ENSG00000170345 ENSG00000198626 ENSG00000198929 ENSG00000121281 ENSG00000198838	ITPR1 PLCE ADCY6 RPS6KA5 ITPR3 GNB MAPK3 ADCY9 FO RYR2 NOS1/ ADCY7 RYF
[hsa04216]()	Ferroptosis	6/1188	39/5573	0.867721158109882	0.999998654289568	ENSG00000168003 ENSG00000110911 ENSG00000169564 ENSG00000104635 ENSG00000100983 ENSG00000141510	SLC3A2 SLC11A2 PCBP1 SLC39A14 GSS TP53
[hsa03430]()	Mismatch repair	3/1188	22/5573	0.878014095222233	0.999998654289568	ENSG00000174371 ENSG00000106628 ENSG00000062822	EXO1 POLD POLD1
[hsa04640]()	Hematopoietic cell lineage	8/1188	51/5573	0.879567477720803	0.999998654289568	ENSG00000136244 ENSG00000085063 ENSG00000213949 ENSG00000095752 ENSG00000272398 ENSG00000164171	IL6 CD59 ITGA1 IL1 CD24 ITGA CSF1 ITGA

						ENSG00000184371<br>ENSG00000005884	
hsa00380	Tryptophan metabolism	5/1188	34/5573	0.879569131133309	0.999998654289568	ENSG00000164904<br>ENSG00000069535<br>ENSG00000120437<br>ENSG00000084754<br>ENSG00000121691	ALDH7A1<br>MAOB ACAT<br>HADHA CA
hsa00511	Other glycan degradation	2/1188	16/5573	0.885217433623028	0.999998654289568	ENSG00000167280<br>ENSG00000162139	ENGASE NEI
hsa04261	Adrenergic signaling in cardiomyocytes	18/1188	106/5573	0.891261275280964	0.999998654289568	ENSG00000128272<br>ENSG00000143153<br>ENSG00000182158<br>ENSG00000149782<br>ENSG00000174233<br>ENSG00000100784<br>ENSG00000043591<br>ENSG00000185386<br>ENSG00000157445<br>ENSG00000058668<br>ENSG00000070961<br>ENSG00000102882<br>ENSG00000188130<br>ENSG00000162104<br>ENSG00000095794<br>ENSG00000198626<br>ENSG00000079337<br>ENSG00000121281	ATF4 ATP1I<br>CREB3L2<br>PLCB3 ADC<br>RPS6KA5<br>ADRB1<br>MAPK11<br>CACNA2D<br>ATP2B4<br>ATP2B1<br>MAPK3<br>MAPK12<br>ADCY9 CRE<br>RYR2<br>RAPGEF3<br>ADCY7
hsa04710	Circadian rhythm	4/1188	29/5573	0.894171839411614	0.999998654289568	ENSG00000133794<br>ENSG00000170485<br>ENSG00000134107<br>ENSG00000100387	ARNTL NPA<br>BHLHE40<br>RBX1
hsa00565	Ether lipid metabolism	5/1188	35/5573	0.894914247688192	0.999998654289568	ENSG00000067113<br>ENSG00000141934<br>ENSG00000111666<br>ENSG00000153982<br>ENSG00000243708	PLPP1 PLPI<br>CHPT1<br>GDPD1<br>PLA2G4B
hsa00512	Mucin type O-glycan biosynthesis	3/1188	23/5573	0.896515491650005	0.999998654289568	ENSG00000008513<br>ENSG00000158470<br>ENSG00000157350	ST3GAL1<br>B4GALT5<br>ST3GAL2
hsa00860	Porphyrin and chlorophyll metabolism	3/1188	23/5573	0.896515491650005	0.999998654289568	ENSG00000090013<br>ENSG00000256269<br>ENSG00000103415	BLVRB HME<br>HMOX2
hsa04920	Adipocytokine signaling pathway	9/1188	58/5573	0.897365823186556	0.999998654289568	ENSG00000109320<br>ENSG00000146232<br>ENSG00000100906<br>ENSG00000110090<br>ENSG00000184557<br>ENSG00000076555<br>ENSG00000117394<br>ENSG00000110931<br>ENSG00000104825	NFKB1<br>NFKBIE<br>NFKBIA<br>CPT1A SOC<br>ACACB<br>SLC2A1<br>CAMKK2<br>NFKBIB
hsa04725	Cholinergic synapse	14/1188	86/5573	0.90350450925187	0.999998654289568	ENSG00000150995<br>ENSG00000128272<br>ENSG00000182158<br>ENSG00000149782<br>ENSG00000174775<br>ENSG00000117013<br>ENSG00000174233<br>ENSG00000096433<br>ENSG00000172354<br>ENSG00000102882<br>ENSG00000162104<br>ENSG00000170345<br>ENSG00000121879<br>ENSG00000121281	ITPR1 ATF<br>CREB3L2<br>PLCB3 HRA<br>KCNQ4<br>ADCY6 ITPI<br>GNB2 MAPI<br>ADCY9 FO<br>PIK3CA<br>ADCY7
hsa04071	Sphingolipid signaling pathway	18/1188	108/5573	0.908073473473029	0.999998654289568	ENSG00000176170<br>ENSG00000109320<br>ENSG00000198964<br>ENSG00000134318<br>ENSG00000149782<br>ENSG00000143753<br>ENSG00000171132<br>ENSG00000132155<br>ENSG00000174775<br>ENSG00000185386<br>ENSG00000102882<br>ENSG00000188130<br>ENSG00000164023<br>ENSG00000143418	SPHK1 NFKI<br>SGMS1<br>ROCK2 PLCI<br>DEGS1 PRK<br>RAF1 HRA<br>MAPK11<br>MAPK3<br>MAPK12<br>SGMS2 CER<br>PTEN PIK3<br>PRKCZ TP5

						ENSG00000171862 ENSG00000121879 ENSG00000067606 ENSG00000141510	
hsa04114	Oocyte meiosis	18/1188	108/5573	0.908073473473029	0.999998654289568	ENSG00000150995 ENSG00000117399 ENSG00000127564 ENSG00000176386 ENSG00000174233 ENSG00000166851 ENSG00000096433 ENSG00000157456 ENSG00000102882 ENSG00000188130 ENSG00000128245 ENSG00000162104 ENSG00000107864 ENSG00000141552 ENSG00000116670 ENSG00000121281 ENSG00000164109 ENSG00000100387	ITPR1 CDC2 PKMYT1 CDC26 ADC PLK1 ITPR CCNB2 MAPK3 MAPK12 YWHAH ADCY9 CPE ANAPC11 MAD2L2 ADCY7 MAD2L1 RB
hsa04650	Natural killer cell mediated cytotoxicity	12/1188	76/5573	0.911552876248557	0.999998654289568	ENSG00000134215 ENSG00000142166 ENSG00000090339 ENSG00000027697 ENSG00000132155 ENSG00000174775 ENSG00000100485 ENSG00000102882 ENSG00000120899 ENSG00000197943 ENSG00000121879 ENSG00000157764	VAV3 IFNAR ICAM1 IFNGR1 RAF HRAS SOS MAPK3 PTK PLCG2 PIK3CA BRA
hsa04514	Cell adhesion molecules (CAMs)	14/1188	87/5573	0.91219605232328	0.999998654289568	ENSG00000124145 ENSG00000105855 ENSG00000163347 ENSG00000090863 ENSG00000142949 ENSG00000146938 ENSG00000090339 ENSG00000108797 ENSG00000181885 ENSG00000154721 ENSG00000198910 ENSG00000073008 ENSG00000162512 ENSG00000197965	SDC4 ITGB CLDN1 GLG PTPRF NLGN4X ICAM1 CNTNAP1 CLDN7 JAM L1CAM PV SDC3 MPZL
hsa00563	Glycosylphosphatidylinositol (GPI)-anchor biosynthesis	3/1188	24/5573	0.912452408804584	0.999998654289568	ENSG00000135845 ENSG00000197858 ENSG00000100564	PIGC GPAA PIGH
hsa00270	Cysteine and methionine metabolism	6/1188	43/5573	0.920998590250469	0.999998654289568	ENSG00000116649 ENSG00000171989 ENSG00000101444 ENSG00000111716 ENSG00000100983 ENSG00000116984	SRM LDHAL AHCY LDH GSS MTR
hsa04610	Complement and coagulation cascades	7/1188	49/5573	0.922435045600561	0.999998654289568	ENSG00000125730 ENSG00000120885 ENSG00000085063 ENSG00000109072 ENSG00000182326 ENSG00000159403 ENSG00000175899	C3 CLU CD VTN C1S C1 A2M
hsa00120	Primary bile acid biosynthesis	1/1188	11/5573	0.928628759004252	0.999998654289568	ENSG00000099377	HSD3B7
hsa00650	Butanoate metabolism	2/1188	19/5573	0.935685416578756	0.999998654289568	ENSG00000120437 ENSG00000084754	ACAT2 HADHA
hsa03320	PPAR signaling pathway	8/1188	58/5573	0.947906628870966	0.999998654289568	ENSG00000173391 ENSG00000167114 ENSG00000164687 ENSG00000110090 ENSG00000099194 ENSG00000147872 ENSG00000117054 ENSG00000166819	OLR1 SLC27A4 FABP5 CPT SCD PLIN ACADM PLI
hsa04727	GABAergic synapse	8/1188	59/5573	0.954138314793976	0.999998654289568	ENSG00000134294 ENSG00000139112 ENSG00000174233	SLC38A2 GABARAPL ADCY6 SR

						ENSG00000197122 ENSG00000172354 ENSG00000162104 ENSG00000121281 ENSG00000102287	GNB2 ADCY ADCY7 GABRE
hsa00770	Pantothenate and CoA biosynthesis	1/1188	13/5573	0.955863527048067	0.999998654289568	ENSG00000127125	PPCS
hsa03030	DNA replication	4/1188	35/5573	0.958639710604445	0.999998654289568	ENSG00000115350 ENSG00000148229 ENSG00000106628 ENSG00000062822	POLE4 POLI POLD2 POLI
hsa00591	Linoleic acid metabolism	1/1188	14/5573	0.965294125944991	0.999998654289568	ENSG00000243708	PLA2G4B
hsa04614	Renin-angiotensin system	1/1188	14/5573	0.965294125944991	0.999998654289568	ENSG00000172009	THOP1
hsa04014	Ras signaling pathway	30/1188	186/5573	0.970895733014369	0.999998654289568	ENSG00000169242 ENSG00000174791 ENSG00000109320 ENSG00000105976 ENSG00000068028 ENSG00000160867 ENSG00000132155 ENSG00000157557 ENSG00000142627 ENSG00000144118 ENSG00000108061 ENSG00000174775 ENSG00000143322 ENSG00000111540 ENSG00000100485 ENSG00000068078 ENSG00000101109 ENSG00000184481 ENSG00000171105 ENSG00000172354 ENSG00000102882 ENSG00000173511 ENSG00000143344 ENSG00000112715 ENSG00000197943 ENSG00000184371 ENSG00000163235 ENSG00000121879 ENSG00000243708 ENSG00000165527	EFNA1 RIN NFKB1 ME RASSF1 FGFR4 RAF ETS2 EPHA RALB SHOC HRAS ABL RAB5B SOS FGFR3 STK FOXO4 INS GNB2 MAPK VEGFB RGL VEGFA PLC CSF1 TGF PIK3CA PLA2G4B ARF6
hsa04979	Cholesterol metabolism	5/1188	44/5573	0.972202905994802	0.999998654289568	ENSG00000123384 ENSG00000130164 ENSG00000134243 ENSG00000144959 ENSG00000021762	LRP1 LDLI SORT1 NCEI OSBPL5
hsa04964	Proximal tubule bicarbonate reclamation	1/1188	15/5573	0.972711031571426	0.999998654289568	ENSG00000143153	ATP1B1
hsa05322	Systemic lupus erythematosus	11/1188	81/5573	0.97307613685546	0.999998654289568	ENSG00000125730 ENSG00000132475 ENSG00000270276 ENSG00000164032 ENSG00000182326 ENSG00000180573 ENSG00000184678 ENSG00000159403 ENSG00000138385 ENSG00000158373 ENSG00000125835	C3 H3F3B HIST2H4B H2AFZ C1 HIST1H2A HIST2H2B C1R SSB HIST1H2B SNRPB
hsa00592	alpha-Linolenic acid metabolism	1/1188	16/5573	0.978543938101319	0.999998654289568	ENSG00000243708	PLA2G4B
hsa03460	Fanconi anemia pathway	6/1188	53/5573	0.981175411529753	0.999998654289568	ENSG00000136492 ENSG00000175279 ENSG00000172732 ENSG00000100726 ENSG00000112039 ENSG00000009413	BRIP1 CENI MUS81 TEL FANCE REV
hsa05033	Nicotine addiction	1/1188	17/5573	0.9831309097651	0.999998654289568	ENSG00000102287	GABRE
hsa04724	Glutamatergic synapse	12/1188	91/5573	0.983639821127479	0.999998654289568	ENSG00000150995 ENSG00000079215 ENSG00000149782 ENSG00000173020 ENSG00000134294 ENSG00000174233 ENSG00000096433 ENSG00000172354	ITPR1 SLC1 PLCB3 GRK SLC38A2 ADCY6 ITPI GNB2 MAPK ADCY9 ADC PLA2G4B

						ENSG00000102882<br>ENSG00000162104<br>ENSG00000121281<br>ENSG00000243708	
hsa05031	Amphetamine addiction	6/1188	54/5573	0.983846716418026	0.999998654289568	ENSG00000128272<br>ENSG00000182158<br>ENSG00000177606<br>ENSG00000069535<br>ENSG00000170345<br>ENSG00000180176	ATF4<br>CREB3L2 JU<br>MAOB FOS T
hsa04728	Dopaminergic synapse	15/1188	109/5573	0.984315401181613	0.999998654289568	ENSG00000150995<br>ENSG00000082701<br>ENSG00000128272<br>ENSG00000182158<br>ENSG00000149782<br>ENSG00000133794<br>ENSG00000105723<br>ENSG00000144285<br>ENSG00000185386<br>ENSG00000096433<br>ENSG00000172354<br>ENSG00000188130<br>ENSG00000069535<br>ENSG00000170345<br>ENSG00000180176	ITPR1 GSK3<br>ATF4<br>CREB3L2<br>PLCB3 ARNT<br>GSK3A SCN<br>MAPK11<br>ITPR3 GNB<br>MAPK12<br>MAOB FOS T
hsa00100	Steroid biosynthesis	1/1188	18/5573	0.986737907461116	0.999998654289568	ENSG00000172893	DHCR7
hsa04744	Phototransduction	1/1188	19/5573	0.98957415695458	0.999998654289568	ENSG00000070729	CNGB1
hsa04020	Calcium signaling pathway	19/1188	136/5573	0.989789395203686	0.999998654289568	ENSG00000176170<br>ENSG00000150995<br>ENSG00000149782<br>ENSG00000276045<br>ENSG00000043591<br>ENSG00000058668<br>ENSG00000070961<br>ENSG00000140795<br>ENSG00000096433<br>ENSG00000162104<br>ENSG00000120899<br>ENSG00000197943<br>ENSG00000198626<br>ENSG00000121281<br>ENSG00000115252<br>ENSG00000086544<br>ENSG00000083454<br>ENSG00000198838<br>ENSG00000067177	SPHK1 ITPR<br>PLCB3 ORA<br>ADRB1<br>ATP2B4<br>ATP2B1<br>MYLK3 ITPR<br>ADCY9 PTK<br>PLCG2 RYR<br>ADCY7 PDE<br>ITPKC P2R<br>RYR3 PHKA
hsa00830	Retinol metabolism	2/1188	30/5573	0.993237233830193	0.999998654289568	ENSG00000167600<br>ENSG00000162496	CYP2S1<br>DHRS3
hsa05034	Alcoholism	20/1188	149/5573	0.995421944604445	0.999998654289568	ENSG00000128272<br>ENSG00000132475<br>ENSG00000182158<br>ENSG00000270276<br>ENSG00000004660<br>ENSG00000164032<br>ENSG00000132155<br>ENSG00000174775<br>ENSG00000100485<br>ENSG00000180573<br>ENSG00000184678<br>ENSG00000172354<br>ENSG00000102882<br>ENSG00000069535<br>ENSG00000061273<br>ENSG00000180176<br>ENSG00000147099<br>ENSG00000110931<br>ENSG00000158373<br>ENSG00000157764	ATF4 H3F3<br>CREB3L2<br>HIST2H4B<br>CAMKK1<br>H2AFZ RAF<br>HRAS SOS<br>HIST1H2A<br>HIST2H2B<br>GNB2 MAPK<br>MAOB HDAC<br>TH HDAC8<br>CAMKK2<br>HIST1H2B<br>BRAF
hsa04152	AMPK signaling pathway	12/1188	108/5573	0.998286594604212	0.999998654289568	ENSG00000182158<br>ENSG00000110092<br>ENSG00000177169<br>ENSG00000110090<br>ENSG00000072310<br>ENSG00000171105<br>ENSG00000099194<br>ENSG00000103319<br>ENSG00000076555<br>ENSG00000121879<br>ENSG00000110931<br>ENSG00000187840	CREB3L2<br>CCND1 ULK<br>CPT1A<br>SREBF1 INS<br>SCD EEF2K<br>ACACB<br>PIK3CA<br>CAMKK2<br>EIF4EBP1

hsa04740	Olfactory transduction	4/1188	61/5573	0.999633900634011	0.999998654289568	ENSG00000173020 ENSG00000070729 ENSG00000115252 ENSG00000137486	GRK2 CNGE[illegible] PDE1A ARR[illegible]
hsa04080	Neuroactive ligand-receptor interaction	12/1188	153/5573	0.999998654289568	0.999998654289568	ENSG00000065325 ENSG00000113580 ENSG00000175591 ENSG00000151090 ENSG00000139874 ENSG00000043591 ENSG00000213903 ENSG00000213218 ENSG00000169006 ENSG00000102287 ENSG00000164251 ENSG00000083454	GLP2R NR3[illegible] P2RY2 THR[illegible] SSTR1 ADR[illegible] LTB4R CSH[illegible] NTSR2 GAB[illegible] F2RL1 P2R[illegible]
